# Identification of candidate sex‐specific genomic regions in male and female Asian arowana genomes

**DOI:** 10.1093/gigascience/giac085

**Published:** 2022-09-15

**Authors:** Xidong Mu, Yi Liu, Chao Liu, Chenxi Zhao, Ruihan Li, Xinxin You, Yexin Yang, Xuejie Wang, Yinchang Hu, Qiong Shi, Chao Bian

**Affiliations:** Key Laboratory of Prevention and Control for Aquatic Invasive Alien Species, Ministry of Agriculture and Rural Affairs, Guangdong Modern Recreational Fisheries Engineering Technology Center, Pearl River Fisheries Research Institute, Chinese Academy of Fishery Sciences, Guangzhou 510380, China; Key Laboratory of Prevention and Control for Aquatic Invasive Alien Species, Ministry of Agriculture and Rural Affairs, Guangdong Modern Recreational Fisheries Engineering Technology Center, Pearl River Fisheries Research Institute, Chinese Academy of Fishery Sciences, Guangzhou 510380, China; Key Laboratory of Prevention and Control for Aquatic Invasive Alien Species, Ministry of Agriculture and Rural Affairs, Guangdong Modern Recreational Fisheries Engineering Technology Center, Pearl River Fisheries Research Institute, Chinese Academy of Fishery Sciences, Guangzhou 510380, China; Shenzhen Key Lab of Marine Genomics, Guangdong Provincial Key Lab of Molecular Breeding in Marine Economic Animals, BGI Academy of Marine Sciences, BGI Marine, BGI, Shenzhen 518083, China; College of Life Sciences, University of Chinese Academy of Sciences, Beijing 100049, China; Shenzhen Key Lab of Marine Genomics, Guangdong Provincial Key Lab of Molecular Breeding in Marine Economic Animals, BGI Academy of Marine Sciences, BGI Marine, BGI, Shenzhen 518083, China; College of Life Sciences, University of Chinese Academy of Sciences, Beijing 100049, China; Shenzhen Key Lab of Marine Genomics, Guangdong Provincial Key Lab of Molecular Breeding in Marine Economic Animals, BGI Academy of Marine Sciences, BGI Marine, BGI, Shenzhen 518083, China; College of Life Sciences, University of Chinese Academy of Sciences, Beijing 100049, China; Key Laboratory of Prevention and Control for Aquatic Invasive Alien Species, Ministry of Agriculture and Rural Affairs, Guangdong Modern Recreational Fisheries Engineering Technology Center, Pearl River Fisheries Research Institute, Chinese Academy of Fishery Sciences, Guangzhou 510380, China; Guangdong Provincial Key Laboratory of Aquatic Animal Immunology and Sustainable Aquaculture, Pearl River Fisheries Research Institute, Chinese Academy of Fishery Sciences, Guangzhou 510380, China; Key Laboratory of Prevention and Control for Aquatic Invasive Alien Species, Ministry of Agriculture and Rural Affairs, Guangdong Modern Recreational Fisheries Engineering Technology Center, Pearl River Fisheries Research Institute, Chinese Academy of Fishery Sciences, Guangzhou 510380, China; Key Laboratory of Prevention and Control for Aquatic Invasive Alien Species, Ministry of Agriculture and Rural Affairs, Guangdong Modern Recreational Fisheries Engineering Technology Center, Pearl River Fisheries Research Institute, Chinese Academy of Fishery Sciences, Guangzhou 510380, China; Shenzhen Key Lab of Marine Genomics, Guangdong Provincial Key Lab of Molecular Breeding in Marine Economic Animals, BGI Academy of Marine Sciences, BGI Marine, BGI, Shenzhen 518083, China; College of Life Sciences, University of Chinese Academy of Sciences, Beijing 100049, China; Shenzhen Key Lab of Marine Genomics, Guangdong Provincial Key Lab of Molecular Breeding in Marine Economic Animals, BGI Academy of Marine Sciences, BGI Marine, BGI, Shenzhen 518083, China; College of Life Sciences, University of Chinese Academy of Sciences, Beijing 100049, China

**Keywords:** Asian arowana, male and female, genome sequencing and resequencing, sex-related genes

## Abstract

**Background:**

Asian arowana, *Scleropages formosus*, is one of the most expensive aquarium fish species worldwide. Its sex, however, cannot be distinguished clearly at any development stage, which impedes captive breeding and species protection for this endangered aquarium fish.

**Results:**

To discover molecular clues to the sex of Asian arowana, we sequenced 26.5 Gb of PacBio HiFi reads and 179.2 Gb of Hi-C reads for 1 male fish and also sequenced 106.5 Gb of Illumina reads, 36.0 Gb of PacBio Sequel reads, and 80.7 Gb of Hi-C reads for 1 female individual. The final male and female genome assemblies were approximately 756.8 Mb and 781.5 Mb in length and contained 25,262 and 25,328 protein-coding genes, respectively. We also resequenced the genomes of 15 male and 15 female individuals with approximately 722.1 Gb of Illumina reads. A genome-wide association study identified several potentially divergent regions between male and female individuals. In these regions, *cd48* and *cfap52* could be candidate genes for sex determination of Asian arowana. We also found some structural variations in few chromosomes between male and female individuals.

**Conclusion:**

We provided an improved reference genome assembly of female arowana and generated the first sequenced genome of 1 male individual. These valuable genetic resources and resequencing data may improve global aquarium fish research.

## Introduction

*Scleropages formosus* (NCBI:txid113540; Fishbase ID: 6357), also known as Asian arowana, belongs to the genus *Scleropages* of the family Osteoglossidae, order Osteoglossiformes. This monophyletic fish order represents an ancient teleost group with a geographic distribution restricted to freshwater river basins. *Scleropages* is a primary group of ancient origin, and their distribution is tied to land/continental evolution [1]. Asian arowana include 3 major varieties (golden, red, and green) in nature. They are widely distributed throughout Southeast Asia, including Cambodia, Indonesia, Laos, the Malay Archipelago, the Philippines, Vietnam, and Thailand [2]. The Asian arowana are also named bonytongue due to their primitive characteristic of large tooth plates on their tongues [3]. A previous study showed that the Sundaland–Indochina species were the sister group of the 2 Australian species within *Scleropages*, and the estimated divergence time of crown-group *Scleropages* ranged from 79.9 to 101.4 Million years ago (Ma) [4].

Asian arowana skin is covered by large and bright conspicuous color scales. Because of the high demand for this species and its high price, overfishing has led to the drastic population decline of Asian arowana. It has been listed as an endangered species by the Convention on International Trade in Endangered Species of Wild Fauna and Flora Appendix I [5].

On the other hand, the sex of Asian arowana is not distinguishable morphologically at any stage of development, even after sexual maturity. Additionally, the mechanism of sex determination is also largely unknown [6]. The lack of a genetic sex identification method critically hinders its further development of captive breeding for aquaculture and species protection for this endangered fish. In previous reports, genetic and genomic methods have been used for sex identification. Sequence-tagged site markers have been identified; however, these markers can only be applied in certain stains, and the accuracy of detection is not high [7]. Shen et al. [8] identified and mapped potentially sex-related genes (such as *dmrt2, dmrt4*, and *sox9*) by transcriptome data and linkage map, while no mutations were found within these sex-related candidate genes.

Regarding the increasing popularity of high-throughput sequencing methodologies, it may be possible to identify sex-determining genes using linkage mapping or a genome-wide association study (GWAS) [9]. The complete genome of Asian arowana was first sequenced in 2015, which was a draft assembly with an N50 scaffold length of 59.0 kb [10]. A chromosome-level genome of a female golden-variety arowana was reported by using a combination of deep shotgun sequencing and high-resolution linkage mapping [11]. In addition, 2 draft genome assemblies for the red and green varieties were also generated. The N50 scaffold sizes of the 3 varieties of genomes were 6.0, 1.6, and 1.9 Mb, respectively, but the N50 contig sizes were very short (30.7, 60.2, and 62.8 kb, respectively). Given that there are still many gaps in the draft genomes of Asian arowana, their inclusion in studies to investigate some biological issues has still been limited. To enhance assembly quality, the wide use of long genomic reads (<100 kb in length) produced by third-generation sequencing technologies can cover long repeat regions and substantially reduce fragmentation [12]. Third-generation sequencing technology can also refine the published draft assemblies to a nearly complete genome by spanning gaps for further genomic analyses [13].

In this study, we combined PacBio third-generation sequencing technology with Illumina second-generation sequencing and Hi-C technologies to assemble male and female genomes of Asian arowana. Transcriptome sequencing and whole-genome resequencing were also performed from both male and female individuals with a particular attention to sex-specific differences.

## Methods

### Sample collection and sequencing

We extracted genomic DNAs from muscle tissues of 1 female and 1 male golden arowana and sequenced them by using an Illumina HiSeq Xten sequencing platform (San Diego, CA, USA; RRID:SCR_016385). The construction of DNA libraries (short-insert sizes of 170, 500, and 800 bp and large-insert sizes of 2, 5, 10, and 20 kb) and subsequent sequencing were performed according to the manufacturer's standard protocols. In total, approximately 106.5 Gb of female raw data were generated (Supplementary Table S1). After filtering adapter sequences and low-quality reads by SOAPnuke v.1.5.6 with detailed parameters, including filter -l 10 -q 0.1 -n 0.01 (RRID:SCR_015025) [14], we obtained 73.4 Gb of Illumina clean reads. We also sequenced the female individual on a PacBio Sequel sequencing platform (Menlo Park, CA, USA; RRID:SCR_017989). A 20-kb library was constructed, and then 4 single-molecule real-time (SMRT) cells were produced using P6 polymerase/C4 chemistry, generating 36.0 Gb of PacBio long reads. After correcting and trimming the PacBio raw reads by using LoRDEC (RRID:SCR_015814) [15] with Illumina short reads, 27.4 Gb of clean PacBio reads were obtained (Supplementary Table S1). To acquire a chromosome-level genome assembly, genomic DNAs from the female muscle tissue were fixed with formaldehyde, sheared by a restriction enzyme (MboI) to build a Hi-C library, and then sequenced by an Illumina HiSeq Xten sequencing platform. A total of 80.7 Gb of 150-bp paired-end Hi-C data were generated (Supplementary Table S1).

The male sample was also collected for construction of a chromosome-level genome assembly. Genome DNAs from the muscle tissues were sequenced on a Pacbio HiFi platform. PacBio recently updated its platforms to generate HiFi reads using the circular consensus sequencing mode with a base-level resolution of over 99% [16, 17]. In total, 26.5 Gb of HiFi reads and 179.2 Gb of Hi-C reads (Supplementary Table S1) were obtained after sequencing.

Genomic DNAs from muscle tissues of 30 golden arowana (15 female and 15 male) were extracted, and 350-bp insert libraries were constructed. A total of 30 libraries were sequenced on an Illumina HiSeq Xten sequencing platform. A total of 722.1 Gb of raw reads were generated, 654.9 Gb of clean reads were obtained through SOAPnuke v.1.5.6 (parameters: filter -l 10 -q 0.1 -n 0.01, RRID:SCR_015025) filtering, and 617.6 Gb of data were mapped (Supplementary Table S2).

### Genome assembly and chromosome linkage

The male and female genome lengths were predicted by a *k*-mer analysis [18] according to the following formula: G = N * (L – *k*-mer + 1)/K_depth, where *k*-mer length is defined as 17 bp, N is the total number of reads, and K_depth represents the frequency of occurrence more frequently than others. The detailed results are shown in Supplementary Figs. S1 and S2.

A hybrid genome assembly pipeline was employed to obtain a female genome assembly. Short Illumina reads were first assembled by using Platanus version 1.2.1 (RRID:SCR_015531) [19]. DBG2OLC [20] was performed to combine Platanus-generated contigs with PacBio long reads to generate a hybrid contig assembly with default parameters. The error-corrected and consensus assembly was generated by minimap2 v2.17 (RRID:SCR_018550) [21] and Racon v1.2.1 (RRID:SCR_017642) [22] using the raw PacBio data. Pilon v1.225 (RRID:SCR_014731) [23] was subsequently employed to polish the hybrid assembly with Illumina short reads. SSPACE-LongRead version 1.1 (RRID:SCR_005056) [24] was applied to construct scaffolds based on PacBio data, and Illumina data were used to join scaffolds through SSPACE version 3.0 (RRID:SCR_005056) [25]. The detailed female assembly pipeline is summarized in Supplementary Fig. S3. This hybrid assembly pipeline was well performed in many previous studies [20, 26, 27]. We performed quality control of Hi-C raw reads and obtained valid Hi-C connected reads by Juicer version 1.5 (RRID:SCR_017226) [28]. The 3-dimensional *de novo* assembly (3D-DNA, version 180922) pipeline [29] was applied to anchor primary scaffolds into chromosome-level scaffolds (Supplementary Fig. S4).

The male genome assembly was constructed with WTDBG2 (RRID:SCR_017225; parameters: -x ccs -g 789m -t 32; RRID:SCR_017225) [30]. We also used the Hi-C data of the male individual to join the male assembly into chromosomes through the Juicer–3D-DNA pipeline (Supplementary Fig. S5). The detailed male assembly pipeline is provided in Supplementary Fig. S3.

### Gene prediction and annotation

Repetitive elements in the female and male assemblies were predicted through a combination of homolog-based and *de novo* approaches. For the homolog-based method, RepeatMasker v4.0.7 (RRID:SCR_012954) [31] and RepeatProteinMask v.4.0.7 [31] were used to detect repeats by alignment against the Repbase database v21.0 [32]. For the *de novo* method, LTR-Finder v.1.0.7 [33] was applied to predict full long terminal repeat retrotransposons. RepeatModeler v1.0.11 [31] was employed to build transposable element (TE) consensus sequences as a *de novo* TE library, and TRF v.4.09 [34] was used to obtain tandem repetitive sequences. RepeatMasker was then used to discover and identify repetitive sequences with the combined library of the *de novo* TEs.

Protein-coding genes were annotated by the BRAKER2 v2.1.5 pipeline (RRID:SCR_018964) [35] with repeat-masked male and female genomes. We masked the repetitive sequence of both genome assemblies, and HISAT2 v0.1.6 (RRID:SCR_015530) was employed to align the transcriptome data to the assembled genomes. Protein sequences of *Danio rerio, Gasterosteus aculeatus, Takifugu rubripes*, and *Tetraodon nigroviridis* downloaded from Ensembl-release99 and the Asian arowana gene protein sequences [36] were used as homology-based evidence. Finally, BRAKER2 was used to annotate the genomes with Augustus version 3.3.3 (RRID:SCR_008417) and GeneMark-ET (v4.46, http://topaz.gatech.edu/genemark/license_download.cgi).

Gene functional annotation was performed based on the consensus of sequence and domain. The protein sequences were aligned to the NCBI Nonredundant Protein Sequence (NR) databases, KEGG [37], SwissProt, and TrEMBL (UniProt release 2020–06) [38] with BLASTp. The domains were searched and predicted by using InterProScan version 5.11 (RRID: SCR_005829) [39, 40] with publicly available databases, including PANTHER [41], Pfam [42], PRINTS [43], ProDom [44], PROSITE profiles [45], and SMART [46]. Gene Ontology terms [47] for each gene were predicted from the InterPro descriptions.

### Transcriptome analysis of ovary and testis tissues

For transcriptome sequencing, total RNAs were collected from 3 ovary tissues of 3 female individuals and 3 testis tissues of 3 male individuals by using TRIzol reagent (Invitrogen, Carlsbad, CA, USA). The reverse transcription step was then performed on these extracted RNAs. Paired-end reads (150 bp) were produced by a HiSeq XTEN platform. Raw data were cleaned by discarding reads with adaptor sequences, >10% of nonsequenced bases, or >50% of low-quality bases by using SOAPnuke v.1.5.6 (parameters: filter -n 0.01 -l 15 -q 0.4 -G -Q 2, RRID:SCR_015025). These filtered RNA reads were mapped onto the female genome assembly by using HISAT2 v0.1.6 (RRID:SCR_015530) with the parameters “–phred33 –sensitive –no-discordant –no-mixed -I 1 -X 1000” [48]. Cufflinks v2.2.1 (RRID:SCR_014597) with default parameters [49] was used to calculate expression values as fragments per kilobase per million mapped reads from 3 ovary samples and 3 testis samples.

### Resequencing analysis

Quality-controlled reads from 30 samples were then aligned to the female assembly by using Burrows Wheeler Aligner v0.7.17 (RRID:SCR_010910) with default parameters [50]. The depth of each base was stated by Samtools v1.7 (RRID:SCR_002105). The BaseRecalibrator and ApplyBQSR module of Genome Analysis Tool Kit v4.1.2.0 (RRID:SCR_001876) [51] was used to correct the base quality. The HaplotypeCaller module was used for variant calling, and the concordant variants were filtered with “QD < 2.0 || MQ < 40.0 || ReadPosRankSum < –8.0 || FS > 60.0|| MQRankSum < –12.5.”

For GWAS, EMMAX [52] with the mixed linear model (MLM) and case control generated by PLINK v1.07 (RRID:SCR_001757) [53] were employed to detect associations based on male and female populations. The score assignment of phenotypic traits of each group in the GWAS analysis included 1 for female individuals and 2 for male individuals. Significance levels of the genotype–phenotype association (*P*) were calculated by using Fisher's exact test under a recessive model. The kinship of each population was measured by Tassel with default parameters, and the R package “qqman” [54] was applied to make Manhattan plots.

### Analysis of chromosome structural variations

A synteny analysis between the genomes of male and female arowana was performed by MUMmer software v4.0beta1 (RRID:SCR_018171) [55]. The alignment of the 2 genomes was completed by the Nucmer module. The alignment identity (>0.9) and alignment length (<2 kb) were retained. Finally, the chromosome synteny regions and structural variations were visualized using RectChr software (https://github.com/BGI-shenzhen/RectChr). To confirm the structure variations, we used the Minimap2 (RRID:SCR_018550) [56] with default parameters to align PacBio HiFi and Pacbio Sequel reads to the male genome and female genome and then employed the Integrative Genomics Viewer software (RRID:SCR_011793) [57] to examine alignments and to show the detailed read coverage of the critical regions in Supplementary Fig. S7 and Supplementary Fig. S8.

## Results

### Genome sequencing and assembly

We sequenced the genome of a female by using an Illumina HiSeq sequencing platform as well as a PacBio Sequel sequencing platform. After data filtering, we obtained a total of 73.4 Gb of clean Illumina short reads and 27.4 Gb of clean PacBio Sequel long reads (Supplementary Table S2). Employing the hybrid assembly method, we obtained a draft genome of 780.9 Mb with a contig N50 of 2.7 Mb. After scaffolding by SSPACE-LongRead and SSPACE, we generated a genome of 781.1 Mb with a scaffold N50 of 4.2 Mb. A total of 80.7 Gb of Hi-C data were analyzed by Juicer, and contigs in the draft assembly were subsequently anchored into chromosomes by a 3D-DNA pipeline, resulting in a polished genome assembly of 781.5 Mb, with an improved scaffold N50 of 29.8 Mb (Table 1). The final assembly of the female individual consisted of 25 chromosomes and covered 765.8 Mb, which accounts for 98.0% of the assembled scaffolds. The length of each chromosome ranged from 18.7 to 55.9 Mb. This female genome was about 90 times more contiguous with a contig N50 of 2.7 Mb relative to a contig N50 of 0.03 Mb of the previous assembly [11].

**Table 1:** Statistics of the male and female genome assemblies

	**Female**				**Male**			
	Scaffold		Contig		Scaffold		Contig	
	Length (bp)	Number	Length (bp)	Number	Length (bp)	Number	Length (bp)	Number
**Max length**	55,928,569		15,345,010		52,996,205		19,790,879	
**N50**	29,809,544	11	2,733,495	86	29,536,427	11	7,818,465	31
**N60**	27,447,229	14	2,158,000	119	28,537,785	13	5,896,906	43
**N70**	27,285,240	16	1,744,810	159	26,679,644	16	4,380,229	57
**N80**	25,552,712	19	1,179,287	213	25,622,179	19	3,272,325	77
**N90**	24,145,000	22	638,362	299	23,750,881	22	1,716,882	108
**Total_length**	781,489,634		780,969,649		756,758,225		756,629,725	
**Number ≥0 bp**		749		1,796		705		823
**Number ≥10,000 bp**		428		1,346		683		823
**Number ≥20,000 bp**		228		1,018		683		823
**GC_rate**	0.4		0.4		0.4		0.4	
**BUSCO**	96.5% [S:93.8%, D:2.7%]				96.1% [S:93.7%, D:2.4%]			

For male individuals, WTDBG2 was used to generate a 756.8-Mb assembly with a contig N50 of 7.8 Mb. Hi-C data were anchored to the draft assembly to form 25 chromosomes (ranging from 18.5 to 53.0 Mb in length) and cover 747.2 Mb, which accounts for approximately 98.7% of the assembled contigs of the male individual.

We also confirmed that approximately 96.5% (93.8% single-copy and 2.7% duplicated) and 96.1% of complete reference genes (93.7% single-copy and 2.4% duplicated) of BUSCO results (version 5.22 and Actinopterygii odb10 reference) [58] were detectable in the final female and male genome assemblies. These results confirm that both assemblies are indeed of high quality and completeness.

### Gene prediction and annotation

In total, approximately 27.8% of the female assembly sequences (similar to the 27.3% of previous female arowana assembly) [11] and 33.4% of the male assembly sequences were annotated as repetitive elements. The repetitive sequences include 129.2 Mb (∼16.5%) of long interspersed elements in the female individual and 134.4 Mb (∼17.8%) in the male individual (Supplementary Tables S3 and S4).

Using the repeat-masked genome assemblies, we predicted a total of 25,328 genes from the female individual and 25,262 from the male individual (Table 2). Compared with the gene number (22,016) of the previous female arowana assembly, we predicted about 3,000 more genes in this female assembly with longer continuous contigs [11]. Based on functional annotation, we predicted 22,250 protein-coding genes (∼87.9%) from the female individual and 21,343 (∼84.6%) protein-coding genes from the male individual with at least 1 assignment from the Swiss-Prot, TrEMBL, NR, KEGG, or InterPro databases.

**Table 2:** Predicted protein-coding genes in the assembled male and female genomes

Evidence	Method/species	Female						Male					
		Number	Average gene length (bp)	Average CDS^a^length (bp)	Average exon per gene	Average exon length (bp)	Average intron length (bp)	Number	Average gene length (bp)	Average CDS length (bp)	Average exon per gene	Average exon length (bp)	Average intron length (bp)
*De novo*	AUGUSTUS	53,315	7,324	968	5.0	193	1,579	31,518	11,076	1,270	7.0	180	1,627
Homolog	*Danio rerio*	21,631	11,497	1,600	8.7	185	1,292	22,569	10,628	15,945	8.2	195	1,260
	*Gasterosteus aculeatus*	25,581	9,030	1,264	7.2	177	1,263	26,637	8,306	1,226	6.7	184	1,252
	*Oryzias latipes*	18,480	11,895	1,773	8.9	198	1,275	20,377	10,680	1,778	8.1	221	1,262
	*Takifugu rubripes*	19,711	10,606	1,418	7.8	182	1,357	22,446	9,820	1,400	7.4	190	1,325
	*Tetraodon nigroviridis*	17,565	11,949	1,549	9.1	171	1,288	17,973	11,320	1,543	8.7	176	1,262
	*Scleropages formosu*	31,620	12,486	1,480	8.0	185	1,575	34,334	10,061	1,305	7.0	186	1,463
Total		25,328	12,358.42	1,584	9.4	167.78	1,276	25,262	11,197	1,546	10.0	154	1,357

^a^Coding regions (CDS).

### Male and female resequencing data

Genome resequencing of 15 males and 15 females generated approximately 722.1 Gb of raw data. The mapping ratio for each sample ranged from 81.4% to 87.9%, and the mean mapped depth was approximately 30-fold. A total of 8.9 million (M) high-confidence single-nucleotide polymorphisms (SNPs) were identified, and they were then annotated based on their positions in the chromosomes. Most of the SNPs (5.4 M, 60.7%) were localized in intergenic regions. Approximately 3.2 M of the SNPs (36.0%) fell in intron regions, and only 0.3 M of the SNPs (3.4%) distributed in coding regions. Among these SNPs within coding regions, 142,646 synonymous SNPs and 122,373 nonsynonymous SNPs were identified (Supplementary Table S5).

### Candidate sex-related loci between male and female individuals

**Figure 1:**
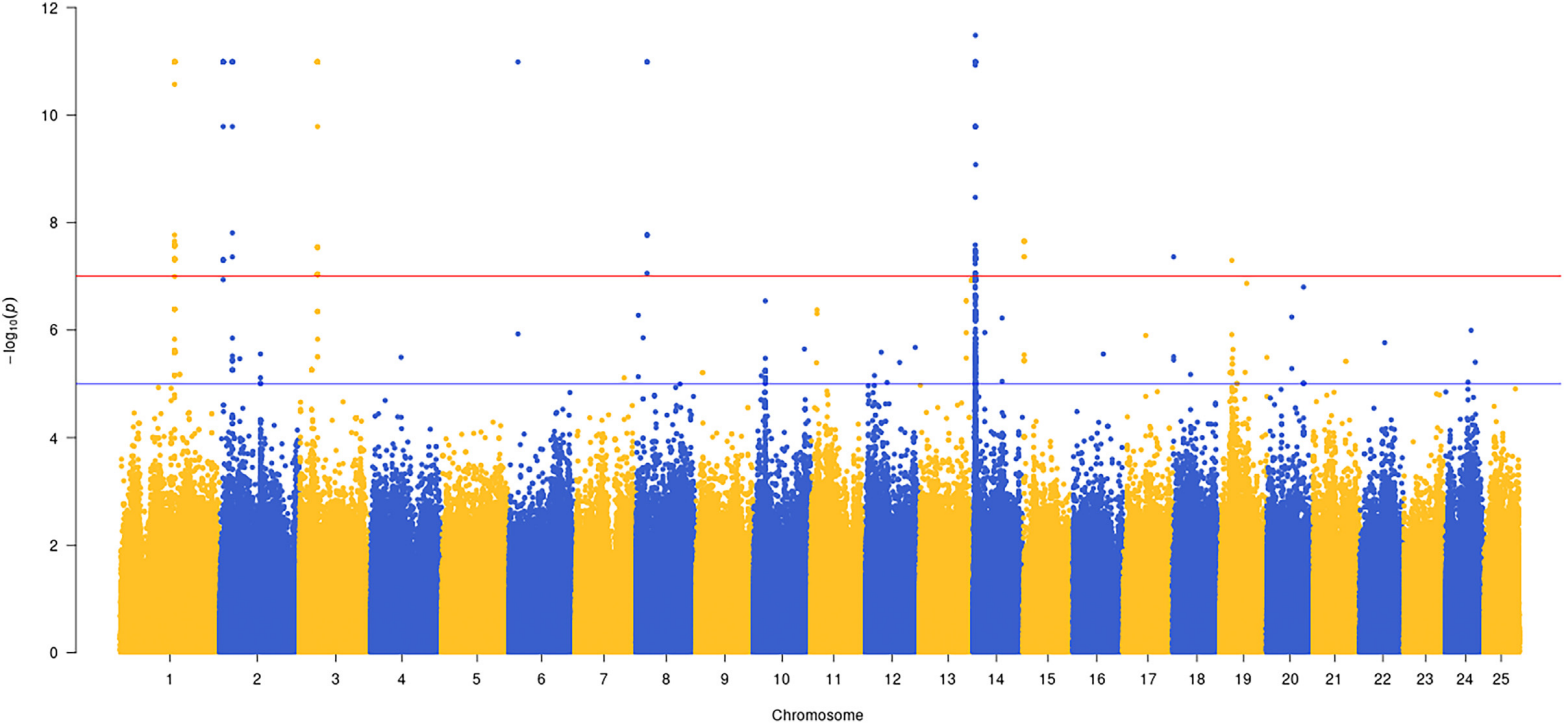
**Manhattan figure demonstrating GWAS results between male and female individuals**. The x- and y-axes represent SNP localizations in chromosomes and corresponding *P* values, respectively.

A GWAS study among the sequenced 15 male and 15 female individuals revealed the most significant peak in Chr14 (Fig. 1). The detailed significant region (*P* = 3.3e-12) in Chr14 ranged from 982,221 to 1,276,785 bp. This contains a *cd48* gene encoding CD48 antigen (Supplementary Table S6). On the other hand, after combining the transcriptome data, we found that a *cfap52* gene (encoding cilia- and flagella-associated protein 52 isoform X1) located in a potential sex divergence region of Chr19 predicted by the GWAS method was more highly expressed in testis than in ovary. It is worth noting that *cfap52* deficiency can result *in situ* inversus totalis and even lead to male infertility [59]. Therefore, we suggest that the *cd48* gene from GWAS results and the *cfap52* from both GWAS and transcriptome results could be candidate sex-related genes in Asian arowana.

### Structural variations between male and female individuals

**Figure 2:**
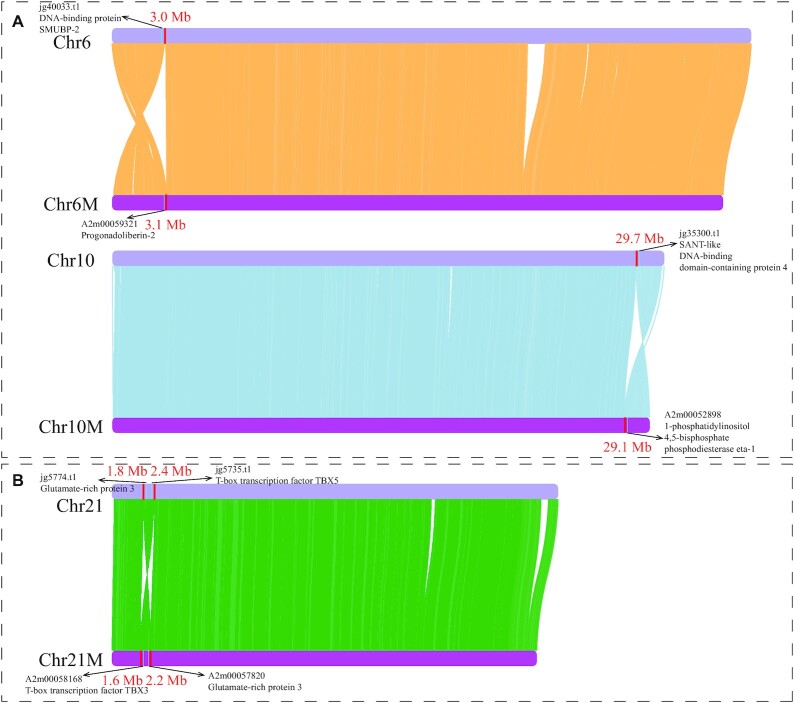
**Chromosomal inversion events between male and female individuals**. Chr6, Chr10, and Chr21 represent chromosomes of the female individual; Chr6M, Chr10M, and Chr21M represent chromosomes of the male individual. (A) Two inversions in the terminal regions of chromosomes. (B) An inversion in the interior regions of Chr21 and Chr21M. Red bars and red numbers represent the boundaries and their sites of chromosome inversions. Gene IDs and functional descriptions with black arrows indicate neighboring genes nearby the boundaries of chromosome inversions.

The female genome was aligned onto the male genome to identify sex differences in chromosome structures (Fig. 2). Aligned regions were over 90% of the total chromosome length of both individuals (Supplementary Table S7). Three potential chromosomal inversions were detected after all-against-all alignments (Fig. 2 and Supplementary Fig. S6). Two chromosome inversions occurred on the terminal regions of Chr6 and Chr10 of the female individual, corresponding to Chr6M (0–3.1 Mb) and Chr10M (29.1–30.5 Mb) of the male individual, respectively (Fig. 2A). Moreover, an inversion occurs in the interior regions of Chr21 of the female, corresponding to Chr21M (1.6–2.2 Mb) of the male (Fig. 2B). These differences in chromosome structure may cause sex divergence between male and female individuals.

## Conclusions

In summary, we generated a high-quality and high-completeness genome assembly of female arowana and sequenced the genome of 1 male individual. GWAS and transcriptome analyses have identified 2 candidate genes that may play sex-determining roles in male and female individuals. Chromosome alignments also showed some potential structural variations between male and female individuals.

These valuable genetic resources including genome and transcriptome data will facilitate the molecular breeding of this economically important fish species.

## Data Availability

The genome sequences of male and female arowana individuals are available in NCBI under accession nos. PRJNA810753 and PRJNA810746. The genome annotation and protein files of male and female individuals are available in the CNGB database [60, 61]. The genome reads, transcriptome reads, and resequencing reads are deposited in the CNSA database [62]. All supporting data and materials are available in the *GigaScience* GigaDB database [63].

## Additional Files

**Supplementary Fig. S1**. 17-*k*-mer analysis for prediction of genome size of the female individual.

**Supplementary Fig. S2**. 17-*k*-mer analysis for prediction of genome size of the male individual.

**Supplementary Fig. S3**. Detailed assembling pipelines of female and male.

**Supplementary Fig. S4**. Heatmap of the Hi-C result of the female individual.

**Supplementary Fig. S5**. Heatmap of the Hi-C result of the male individual.

**Supplementary Fig. S6**. Chromosomal alignments of male and female chromosomes.

**Supplementary Fig. S7**. Integrated Genome Visualization screenshot of inversion boundaries of Chr6M, Chr10M, and Chr21M chromosomes.

**Supplementary Fig. S8**. Integrated Genome Visualization screenshot of inversion boundaries of Chr6, Chr10, and Chr21 chromosomes.

**Supplementary Table S1**. Summary of sequenced reads for male and female genomes.

**Supplementary Table S2**. Summary of map ratio for the 30 male and female samples.

**Supplementary Table S3**. Repetitive elements in the assembled genome of a female individual.

**Supplementary Table S4**. Repetitive elements in the assembled genome of a male individual.

**Supplementary Table S5**. Chromosome location of SNPs.

**Supplementary Table S6**. Genes in potential sex divergence regions in chromosomes predicted by the GWAS and their expression values in ovary and testis tissues.

**Supplementary Table S7**. Statistics of the mapped ratio of chromosomes of male and female individuals.

giac085_GIGA-D-22-00043_Original_Submission

giac085_GIGA-D-22-00043_Revision_1

giac085_GIGA-D-22-00043_Revision_2

giac085_Response_to_Reviewer_Comments_Original_Submission

giac085_Response_to_Reviewer_Comments_Revision_1

giac085_Reviewer_1_Report_Original_Submission Jason R. Miller, MS -- 3/27/2022 Reviewed

giac085_Reviewer_2_Report_Original_Submission Christiaan Henkel -- 4/4/2022 Reviewed

giac085_Reviewer_2_Report_Revision_1 Christiaan Henkel -- 7/7/2022 Reviewed

giac085_Supplemental_File

## Abbreviations

bp: base pairs; BUSCO: Benchmarking Universal Single-Copy Orthologs; Gb: gigabase; GWAS: genome-wide association study; KEGG: Kyoto Encyclopedia of Genes and Genomes; Mb: megabase; NCBI: National Center for Biotechnology Information; NR: Nonredundant Protein Sequence; SNP: single-nucleotide polymorphism; TE: transposable element; 3D-DNA: 3-dimensional *de novo* assembly.

## Competing Interests

The authors declare that they have no competing interests.

## Funding

This study was supported by the Central Public-Interest Scientific Institution Basal Research Fund, CAFS (no. 2016HY-ZCO402, no. 2019ZD0503, no. 2020TD17), the Guangdong Provincial Special Fund for Modern Agriculture Industry Technology Innovation Team (2022KJ150), China-ASEAN Maritime Cooperation Fund (no. CAMC-2018F), Guangzhou Scientific Planning Program (no. 201904010409), and National Freshwater Genetic Resource Center (FGRC18537).

## Authors’ Contributions

X.M. and C.B. designed the research; Y.L., C.L., and Y.Y. collected samples and conducted experiments; C.L., X.W., and Y.H. performed artificial breeding; C.B., C.Z., R.L., X.Y., Q.S., and X.M. analyzed the data; C.B., Y.L., and X.M. wrote the manuscript; and C.B., Q.S., and X.M. revised the manuscript.
