# Supplementary material for: Identification of candidate sex‐specific genomic regions in male and female Asian arowana genomes
Source: Gigascience. 2022 Sep 15;11:giac085. doi: 10.1093/gigascience/giac085 (PMC9475665; doi:10.1093/gigascience/giac085)
Supplement: giac085_GIGA-D-22-00043_Original_Submission [file giac085_giga-d-22-00043_original_submission.pdf]

GigaScience

Sex-specific genomic region identification in male and female Asian arowana genomes

--Manuscript Draft--

Manuscript Number:	GIGA-D-22-00043
Full Title:	Sex-specific genomic region identification in male and female Asian arowana genomes
Article Type:	Data Note
Funding Information:	Guangdong Provincial Special Fund for Modern Agriculture Industry Technology Innovation Team (2022KJ150) — Professor Xidong Mu
Abstract:	Background<br><br>The Asian arowana, Scleropages formosus, is one of the most famous and expensive aquarium fish species worldwide. The sex of Asian arowana, however, cannot be distinguished clearly at any development stage, which impedes captive breeding and species protection for this endangered aquarium fish.<br><br>Results<br><br>To discover the molecular clues of the sex of Asian arowana, we sequenced the genomes of male and female individuals de novo with 367.8 Gb of raw Illumina reads, 27.4 Gb of PacBio long reads and 80.7 Gb of HiC reads. The final male and female assemblies were approximately 789.1 Mb and 781.5 Mb in size and contained 25,244 and 25,328 protein-coding genes. We also resequenced the genomes of 15 male and 15 female individuals with approximately 709.2 Gb Illumina reads. Genome-wide association study (GWAS) has identified several remarkably divergent regions between male individuals and female individuals. In these regions, the cd48 and cfap52 genes could be candidate genes for the determination of Asian arowana sex. We also found some structural variations in chromosomes between male and female individuals.<br><br>Conclusion<br><br>We provided an improved reference genome assembly of female arowana and generated the first sequenced genome of male individuals. These large and valuable genome assemblies and resequencing data will improve global aquarium fish research.
Corresponding Author:	Chao Bian<br><br>CHINA
Corresponding Author Secondary Information:	
Corresponding Author's Institution:	
Corresponding Author's Secondary Institution:	
First Author:	Chao Bian
First Author Secondary Information:	
Order of Authors:	Chao Bian
	Xidong Mu
	Yi Liu
	Chao Liu
	Chenxi Zhao
	Ruihan Li

Powered by Editorial Manager® and ProduXion Manager® from Aries Systems Corporation

	Xinxin You
	Yexin Yang
	Xuejie Wang
	Yinchang Hu
	Qiong Shi
**Order of Authors Secondary Information:**	
**Additional Information:**	
**Question**	**Response**
Are you submitting this manuscript to a special series or article collection?	No
**Experimental design and statistics**<br><br>Full details of the experimental design and statistical methods used should be given in the Methods section, as detailed in our Minimum Standards Reporting Checklist. Information essential to interpreting the data presented should be made available in the figure legends.<br><br>Have you included all the information requested in your manuscript?	Yes
**Resources**<br><br>A description of all resources used, including antibodies, cell lines, animals and software tools, with enough information to allow them to be uniquely identified, should be included in the Methods section. Authors are strongly encouraged to cite Research Resource Identifiers (RRIDs) for antibodies, model organisms and tools, where possible.<br><br>Have you included the information requested as detailed in our Minimum Standards Reporting Checklist?	Yes
**Availability of data and materials**<br><br>All datasets and code on which the conclusions of the paper rely must be	Yes

Powered by Editorial Manager® and ProduXion Manager® from Aries Systems Corporation

either included in your submission or deposited in publicly available repositories (where available and ethically appropriate), referencing such data using a unique identifier in the references and in the “Availability of Data and Materials” section of your manuscript.<br><br>Have you have met the above requirement as detailed in our Minimum Standards Reporting Checklist?	

Manuscript
Click here to access/download;Manuscript;manuscript 20220224 aje.docx

Sex-specific genomic region identification in male and female Asian arowana genomes

Xidong Mu[1†]*, Yi Liu [1†], Chao Liu[1], Chenxi Zhao[2, 3], Ruihan Li[2, 3], Xinxin You[2, 3], Yexin Yang[1,4], Xuejie Wang[1], Yinchang Hu[1], Qiong Shi[2, 3], Chao Bian[2, 3]*

[1.]Key Laboratory of Prevention and Control for Aquatic Invasive Alien Species, Ministry of Agriculture and Rural Affairs, Guangdong Modern. Recreational Fisheries Engineering Technology Center, Pearl River Fisheries Research Institute, Chinese Academy of Fishery Sciences, Guangzhou, 510380, China

[2]Shenzhen Key Lab of Marine Genomics, Guangdong Provincial Key Lab of Molecular Breeding in Marine Economic Animals, BGI Academy of Marine Sciences, BGI Marine, BGI, Shenzhen, Guangdong 518083, China

[3]College of Life Sciences, University of Chinese Academy of Sciences, Beijing, China

[4]Key Laboratory of Aquatic Animal Immune Technology of Guangdong Province, Guangzhou, 510380, China

† These authors contributed equally to this study

*Correspondence and requests for materials should be addressed to Xidong Mu (email: muxd@prfri.ac.cn) and Chao Bian (email: bianchao@genomics.cn)

Abstract

Background: The Asian arowana, *Scleropages formosus*, is one of the most famous and expensive aquarium fish species worldwide. The sex of Asian arowana, however, cannot be distinguished clearly at any development stage, which impedes captive breeding and species protection for this endangered aquarium fish.

Results: To discover the molecular clues of the sex of Asian arowana, we sequenced the genomes of male and female individuals *de novo* with 367.8 Gb of raw Illumina reads, 27.4 Gb of PacBio long reads and 80.7 Gb of HiC reads. The final male and

female assemblies were approximately 789.1 Mb and 781.5 Mb in size and contained 25,244 and 25,328 protein-coding genes. We also resequenced the genomes of 15 male and 15 female individuals with approximately 709.2 Gb Illumina reads. Genome-wide association study (GWAS) has identified several remarkably divergent regions between male individuals and female individuals. In these regions, the *cd48* and *cfap52* genes could be candidate genes for the determination of Asian arowana sex. We also found some structural variations in chromosomes between male and female individuals.

Conclusion: We provided an improved reference genome assembly of female arowana and generated the first sequenced genome of male individuals. These large and valuable genome assemblies and resequencing data will improve global aquarium fish research.

Key words: Asian arowana; male and female; genome sequencing and resequencing; sex-related genes

Introduction

Scleropages formosus, also known as Asian arowana, belongs to the genus *Scleropages* of family Osteoglossidae, order Osteoglossiformes. This monophyletic fish order represents an ancient teleost group with a geographic distribution restricted to freshwater river basins. *Scleropages* are a primary group of ancient origin, and their distribution is tied to land/continental evolution [1]. Asian arowana include three major varieties (the golden, red and green varieties) in nature. They are widely distributed throughout Southeast Asia, including Cambodia, Indonesia, Laos, the Malay Archipelago, the Philippines, Vietnam and Thailand [2]. The Asian arowana are also named bonytongue due to their primitive characteristic of large tooth plates on their tongues [3]. A previous study showed that the Sundaland–Indochina species were the sister group of the two Australian species within *Scleropages,* and the

estimated divergence time of crown-group *Scleropages* ranged from 79.9 Ma to 101.4 Ma [4].

Asian arowana skin is covered by large and bright conspicuous color scales. In addition to this beautiful appearance, the Asian arowana is considered a symbol of lucky, making it a highly valued aquarium fish in the world, particularly in Asian countries [4]. Because of the high demand for this species and its high price, overfishing has led to the drastic population decline of Asian arowana. The Asian arowana has been listed as an endangered species by the Convention on International Trade in Endangered Species of Wild Fauna and Flora (CITES) Appendix I [5].

On the other hand, the sex of Asian arowana is not distinguishable externally at any stage of development, even after sexual maturity. Additionally, the mechanism of sex determination is also largely unknown [6]. The lack of a genetic sex identification method critically hinders its further development of captive breeding for aquaculture and species protection for this endangered fish. In previous reports, genetic and genomic methods have been used for sex identification. Sequence-tagged site (STS) markers have been identified; however, these markers can only be used in a certain stain, and the accuracy of detection is not high [7]. Shen et al. (2014) identified and mapped potentially sex-related genes (dmrt2, dmrt4 and sox9, etc.) by transcriptome data and linkage map, and no mutations were found in the potential sex candidate genes [8].

Regarding the increasing popularity of high-throughput sequencing methodologies, it may be possible to identify sex-determining genes using linkage mapping or genome-wide association study (GWAS)[9]. The complete genome of Asian arowana was first sequenced in 2015, which was a draft assembly with an N50 scaffold length of 59.0 kb [10]. A chromosome-level genome of a female golden-variety arowana was reported by using a combination of deep shotgun sequencing and high-resolution linkage mapping [11]. In addition, two draft genome assemblies for the red and green varieties were also generated. The N50 scaffold sizes of the three

varieties genomes were 6.0, 1.6 and 1.9 Mb, respectively. Given that there are still many gaps in the draft genomes of Asian arowana, their inclusion in studies investigating some biological questions has been limited. To enhance assembly quality, the wide use of long genomic reads (<100 kb in length) produced by third-generation sequencing technologies can cover long repeat regions and substantially reduce fragmentation [12]. Third-generation sequencing technology can also refine the published draft assemblies to a nearly complete genome by spanning gaps for further genomic analyses [13].

In this study, we combined third-generation sequencing technology with second-generation sequencing and Hi-C technologies to assemble male and female genomes of Asian arowana. Transcriptome sequencing and whole genome resequencing were also performed from both males and females with particular attention to sex-specific differences.

Methods

Sample collection and sequencing

We extracted genomic DNA from muscle tissues of female golden arowana and male golden arowana and sequenced them by using the Illumina HiSeq Xten sequencing platform (San Diego, CA, USA). The construction of DNA libraries (short-insert sizes of 500 and 800 bp, large-insert sizes of 2 kb, 5 kb, 10 kb, 20 kb and 40 kb) and subsequent sequencing were performed according to standard protocols. In total, approximately 226.2 Gb of female raw data was generated (Supplementary Table 1). After filtering by SOAPnuke v.1.5.6 (RRID:SCR_015025) [14], we obtained 148.1 Gb of Illumina clean reads. The raw Illumina reads of the male sample were 571.2 Gb, and the filtered reads were 414.9 Gb (Supplementary Table 1). We also sequenced female individuals on the PacBio Sequel sequencing platform (Menlo Park, CA, USA). A 20 kb library was constructed for PacBio, and then 4 SMRT cells were produced using P6 polymerase/C4 chemistry, producing 36.0 Gb of PacBio long

reads. After correcting and trimming the PacBio reads by using LoRDEC (RRID:SCR_015814) [15] with Illumina short reads, 27.4 Gb clean PacBio reads were obtained (Supplementary Table 1).

To acquire a chromosome-level genome assembly, genomic DNA from female muscle tissue was fixed with formaldehyde, sheared by a restriction enzyme (MboI) to build a Hi-C library, and then sequenced by the Illumina HiSeq Xten sequencing platform. A total of 80.7 Gb of 150 paired-end Hi-C data were generated (Supplementary Table 1).

Genomic DNA from muscle tissues of 30 golden arowana (15 female and 15 male) were extracted, and 350 bp insert libraries were constructed. A total of 30 libraries were sequenced on the Illumina HiSeq Xten sequencing platform (San Diego, CA, USA). A total of 722.0 Gb raw reads were generated, and 638.5 Gb clean reads were obtained through Soapnuke v.1.5.6 (RRID: SCR_015025) filtering (Supplementary Table 2).

Genome assembly and chromosome linkage

The male and female genomes were predicted by a k-mer analysis [16] according to the following formula: $G = N*(L–17 + 1)/K\_depth$, where N is the total number of reads and K_depth represents the frequency of occurrence more frequently than others.

A hybrid genome assembly pipeline was employed to obtain a female individual genome assembly. Short Illumina reads were first assembled by using Platanus version 1.2.1 (RRID:SCR_015531) [17], and DBG2OLC (Ye et al., 2016) was performed to combine Platanus-generated contigs with PacBio reads to generate a hybrid contig assembly with default parameters. The error-corrected and consensus assembly was generated by minimap2 v2.17 (RRID:SCR_018550) [18] and Racon v1.2.1 (RRID:SCR_017642) [19] using the raw PacBio data. Pilon v1.225 (RRID:SCR_014731) [20] was subsequently employed to polish the hybrid assembly

with Illumina short reads. SSPACE-LongRead version 1.1 (RRID:SCR_005056) [21] was applied to construct scaffolds based on PacBio data, and Illumina data were used to join scaffolds through SSPACE version 3.0 (RRID:SCR_005056) [22].We performed quality control of Hi-C raw reads and obtained valid Hi-C connected reads by Juicer version 1.5 (RRID:SCR_017226) [23]. The 3D *de novo* assembly (3D-DNA, version 180922) pipeline [24] was applied to anchor primary contigs into chromosome-level scaffolds (Supplementary Figures 3 and 4).

The filtered Illumina sequencing data of the male individual were assembled by Soapdenovo2 version 2.04.4 (RRID:SCR_014986) [25]. SSPACE was performed to connect the scaffold, and GapCloser from the Soapdenovo2 package was used to fill the gaps. We also used the Hi-C data of females to join the assembly of males into chromosomes through the Juicer-3D-DNA pipeline.

Gene prediction and annotation

Repetitive elements in the female and male assemblies were identified through a combination of homolog-based and *de novo* approaches. For the homolog-based method, RepeatMasker v4.0.7 (RRID:SCR 012954) [26] and RepeatProteinMask v.4.0.7 [26] were used to detect repeats by alignment against the Repbase database v21.0 [27]. For the *de novo* method, LTR-Finder v.1.0.7 [28] was applied to predict full long terminal repeat (LTR) retrotransposons. RepeatModeler v1.0.11 [26] was employed to build transposable element (TE) consensus sequences as a *de novo* TE library, and TRF v.4.09 [29] was used to obtain tandem repetitive sequences. RepeatMasker was then used to discover and identify repetitive sequences with the combined library of the *de novo* TEs.

Protein-coding genes were annotated by the BRAKER2 v2.1.5 pipeline (RRID:SCR_018964) [30] with repeat-masked male and female genomes. We masked the repetitive sequence of both genomes, and HISAT2 v0.1.6 (RRID:SCR_015530) was employed to align the transcriptome data to the genome. Protein sequences of

Danio rerio, Gasterosteus aculeatus, Takifugu rubripes and *Tetraodon nigroviridis* downloaded from Ensembl-release99 and the Asian arowana gene protein sequences [31] were used as homology-based evidence. Finally, BRAKER2 was used to annotate the genome with Augustus version 3.3.3 (RRID:SCR 008417) and GeneMark-ET (v4.46, topaz.gatech.edu/license download.cgi).

Gene functional annotation was performed based on the consensus of sequence and domain. The protein sequences were aligned to the NCBI Non-Redundant Protein Sequence (NR) databases, Kyoto Encyclopedia of Genes and Genomes (KEGG)[32], SwissProt and TrEMBL (UniProt release 2020-06) [33] with BLASTp. The domains were searched and predicted by using InterProScan version 5.11 (RRID: SCR_005829) [34, 35] with publicly available databases, including PANTHER [36], Pfam [37], PRINTS [38], ProDom [39], PROSITE profiles [40], and SMART [41]. Gene ontology (GO) terms [42] for each gene were predicted from the InterPro descriptions.

Transcriptome analysis of ovary and testis tissues

For transcriptome sequencing, RNA was collected from three ovary tissues of three female individuals and three testis tissues of three male individuals by using TRIzol reagent (Invitrogen, Carlsbad, CA, USA). The reverse transcription step was then performed on the extracted RNA. Paired-end reads (150 bp) were produced by the HiSeq XTEN platform. Raw data were cleaned by discarding reads with adaptors, with >10% of N bases or with >50% of low-quality bases. These filtered RNA reads were mapped onto the female genome assembly by using HISAT2 v0.1.6 (RRID:SCR_015530) with the parameters “--phred33 --sensitive --no-discordant --no-mixed -I 1 -X 1000” [43]. Cufflinks v2.2.1 (RRID:SCR_014597) with default parameters [44] was used to calculate expression values from three ovary samples and three testis samples. EdgeR software (RRID:SCR_012802) [45] was employed to

identify the differentially expressed genes (DEGs, P value <0.05 and folds >2) and draw the heatmap (Supplementary Figure 5).

Resequencing analysis

Quality-controlled reads from 30 samples were then aligned to the female assembly by using Burrows Wheeler Aligner v0.7.17 (BWA; RRID:SCR_010910) with default parameters [46]. The depth of each base was stated by Samtools v1.7 (RRID:SCR_002105). The BaseRecalibrator and ApplyBQSR module of Genome Analysis Tool Kit v4.1.2.0 (GATK; RRID:SCR_001876) [47] was used to correct the base quality. The HaplotypeCaller module was used for variant calling, and the concordant variants were filtered with "QD < 2.0 || MQ < 40.0 || ReadPosRankSum < -8.0 || FS > 60.0|| MQRankSum < -12.5".

For genome-wide association studies (GWAS), EMMAX [48] with the MLM and case control generated by PLINK v1.07 (RRID:SCR_001757)[49] were employed to detect association analysis based on male and female populations. The score assignment of phenotypic traits of each group in the GWAS analysis included 1 for female individuals and 2 for male individuals. Significance levels of genotype-phenotype association (*p*) were calculated by using Fisher’s exact test under a recessive model. The kinship of each population was measured by Tassel with default parameters, and the R package ‘qqman’ [50] was applied to make Manhattan plots.

Analysis of chromosome structural variations

Synteny analysis between the genomes of male and female arowana was performed by MUMmer software v4.0beta1 (RRID:SCR_018171) [51]. The alignment of the two genomes was completed by the Nucmer module. The alignment identity (>0.9) and alignment length (<2 kb) were retained. Finally, the chromosome synteny regions and structural variations were visualized using RectChr software (https://github.com/BGI-shenzhen/RectChr).

Results

Genome sequencing and assembly

We sequenced the genome of a female by using an Illumina HiSeq sequencing platform as well as a PacBio Sequel sequencing platform. After data filtering, we obtained a total of 98.1 Gb of clean Illumina data and 27.4 Gb of PacBio long reads (Supplementary Table 2). Employing a hybrid assembly method, we obtained a draft genome of 780.9 Mb with a contig N50 of 2.7 Mb. After scaffolding by SSPACE-longer and SSPACE, we generated a genome of 781.1 Mb with a scaffold N50 of 4.2 Mb. A total of 80.7 Gb of Hi-C data were analyzed by Juicer, and contigs in the draft assembly were subsequently anchored into chromosomes by a 3D-DNA pipeline, resulting in a polished genome assembly of 781.5 Mb, with an improved scaffold N50 of 29.8 Mb (Table 1). The final assembly of the female individual consisted of 25 chromosomes and covered 765.8 Mb, which accounts for 98.0% of the whole genome. The length of each chromosome ranged from 18.7 Mb to 55.9 Mb.

For male individuals, Soapdenovo2, SSPACE and GapCloser were used to obtain a 789.1 Mb scaffold assembly with N50 7.3 Mb. Subsequently, Hi-C data were anchored to its draft assembly to 25 chromosomes (ranging from 18.6 Mb to 54.0 Mb in length) and covers 763.5 Mb, which accounts for approximately 96.7% of scaffolds of male individuals.

We also confirmed that approximately 95.4% (92.4% single-copy and 3.0% duplicated) and 96.2% of complete reference genes (93.4% single-copy and 2.8% duplicated) of BUSCO results were detectable in the final female and male assemblies. These results confirm that both genome assemblies are indeed of high quality and completeness.

Gene prediction and annotation

In total, approximately 27.8% of the female assembly sequences and 26.6% of the male assembly sequences were annotated as repetitive elements. The repetitive sequences include 129.2 Mb (~16.5%) of long interspersed elements (LINEs) in female individuals and 104.7 Mb (~13.3%) in male individuals (Table 3 and Supplementary Tables 3 and 4).

Using the repeat-masked genome assembly, we predicted a total of 25,328 genes from female individuals and 25,244 from male individuals (Table 4). Based on functional annotation, we predicted 22,250 protein-coding genes (~87.9%) from female individuals and 21,355 (~84.6%) protein-coding genes from male individuals with at least one assignment from the Swiss-Prot, TrEMBL, Nr, KEGG or InterPro databases.

Male and female resequencing data

Genome resequencing of 15 males and 15 females revealed approximately 709.1 Gb of raw data. The mapping ratio for each sample ranged from 81.4% to 87.9%, and the mean mapped depth was approximately 30-fold (Supplementary Table S2). A total of 8.9 million (M) high-confidence SNPs were identified, and they were then annotated based on their positions in the chromosomes. Most of the SNPs (5.4 M, 60.7%) were located in intergenic regions. Approximately 3.2 M of the SNPs (36.0%) fell in intron regions, and only 0.3 M of the SNPs (3.4%) were distributed in coding regions. Among these SNPs within coding regions, 142,646 synonymous SNPs and 122,373 nonsynonymous SNPs were identified (Supplementary Table S5).

Candidate sex-related loci and DEGs between male and female individuals

A GWAS result among the sequenced 15 male and 15 female individuals showed the most significant peak in Chr14. The detailed significant region (P value= 3.3e-12) in Chr14 ranged from 982,221 bp to 1,276,785 bp. This contains a *cd48* gene encoding CD48 antigen (Supplementary Table 6). On the other hand, after combining the

transcriptome data, we found that the *cfap52* gene (encoding cilia- and flagella-associated protein 52 isoform X1) located in a potential sex divergence region of Chr19 predicted by the GWAS method was remarkably more highly expressed in testis tissue than in ovary tissue. It is worth noting that *cfap52* deficiency can result in situs inversus totalis and even lead to male infertility [52]. Therefore, we suggest that the *cd48* gene from GWAS results and the *cfap52* from both GWAS and transcriptome results could be candidate sex determination genes for Asian arowana.

Structural variations between male and female individuals

The genome of female individuals was aligned onto the male genome to identify sex differences in chromosome structures (Figure 2). Aligned regions were over 90% of the total chromosome length of both individuals (Supplementary Table 7). Five significant chromosomal inversions were detected after all-against-all alignments (Supplementary Figure 6). Four chromosome inversions occurred on the terminal regions of Chr3, Chr6, Chr10 and Chr19 of the female individual, corresponding to Chr4M (29.8-38.2 Mb), Chr6M (0-3.0 Mb), Chr10M (29.6-31.2 Mb) and Chr19M (22.6-25.7 Mb) of the male individual, respectively (Figure 2B). Moreover, an inversion occurs in the interior regions of Chr21 of the female, corresponding to Chr21M (1.6-2.2 Mb) of the male (Figure 2B). These differences in chromosome structure may cause sex divergence between male and female individuals.

Conclusion

In summary, we generated a high-quality and high-completeness genome assembly of female arowana and sequenced the first genome of male individuals. GWASs and transcriptome analyses have identified two core genes that could be sex determination genes in male and female individuals. Chromosome alignments also showed some remarkable structural variations between male and female individuals.

These large amounts of genomic data containing genome and transcriptome data will facilitate the molecular breeding of this economically important fish species.

Data Availability

The genome annotation files of male and female arowana individuals are available in NCBI under accession Nos. PRJNA810753 and PRJNA810746. The genome annotation and protein files of male and female individuals are available in the GigaScience database.

Additional Files

Supplementary Figure 1. 17-kmer analysis for prediction of genome size of the female individual.

Supplementary Figure 2. 17-kmer analysis for prediction of genome size of the male individual.

Supplementary Figure 3. Heatmap of the Hi-C result of female individual.

Supplementary Figure 4. Heatmap of the Hi-C result of male individual.

Supplementary Figure 5. A heatmap of DEGs in the testis and ovary tissues of three male and three female individuals.

Supplementary Figure 6. Chromosomal alignments of male and female chromosomes.

Supplementary Table 1. Summary of sequenced reads for male and female genomes.

Supplementary Table 2. Summary of map ratio for the 30 male and female samples.

Supplementary Table 3. Repetitive elements in the assembled genome of a female individual.

Supplementary Table 4. Repetitive elements in the assembled genome of a male individual.

Supplementary Table 5. Chromosome location of SNPs.

Supplementary Table 6. Genes in potential sex divergence regions in chromosomes predicted by the GWAS and their expression values in ovary and testis tissues.

Supplementary Table 7. Statistics of the mapped ratio of chromosomes of male and female individuals.

Abbreviations

BUSCO: Benchmarking Universal Single-Copy Orthologs; GATK: Genome Analysis Toolkit; Gb: gigabase; Mb: megabase; ML: maximum likelihood; NCBI: National Center for Biotechnology Information; SNP: single-nucleotide polymorphism.

Competing Interests

The authors declare that they have no competing interests.

Funding

This study was supported by the Guangdong Provincial Special Fund for Modern Agriculture Industry Technology Innovation Team (2022KJ150), Grant Plan for Demonstration City Project for Marine Economic Development in Shenzhen (No. 86), China-ASEAN Maritime Cooperation Fund (No. CAMC-2018F), Guangzhou Scientific Planning Programs (No. 201904010409) and National Freshwater Genetic Resource Center (FGRC18537).

Authors' Contributions

XD Mu and C Bian designed the research; Y Liu, C Liu and YX Yang collected the specimens and conducted the experiment; C Liu, XJ Wang and YC Hu were responsible for artificial breeding; C Bian, CX Zhao, RH Li, XX You, Q Shi and XD Mu analyzed the data; C Bian, Y Liu and XD Mu wrote the manuscript; all authors revised the manuscript.

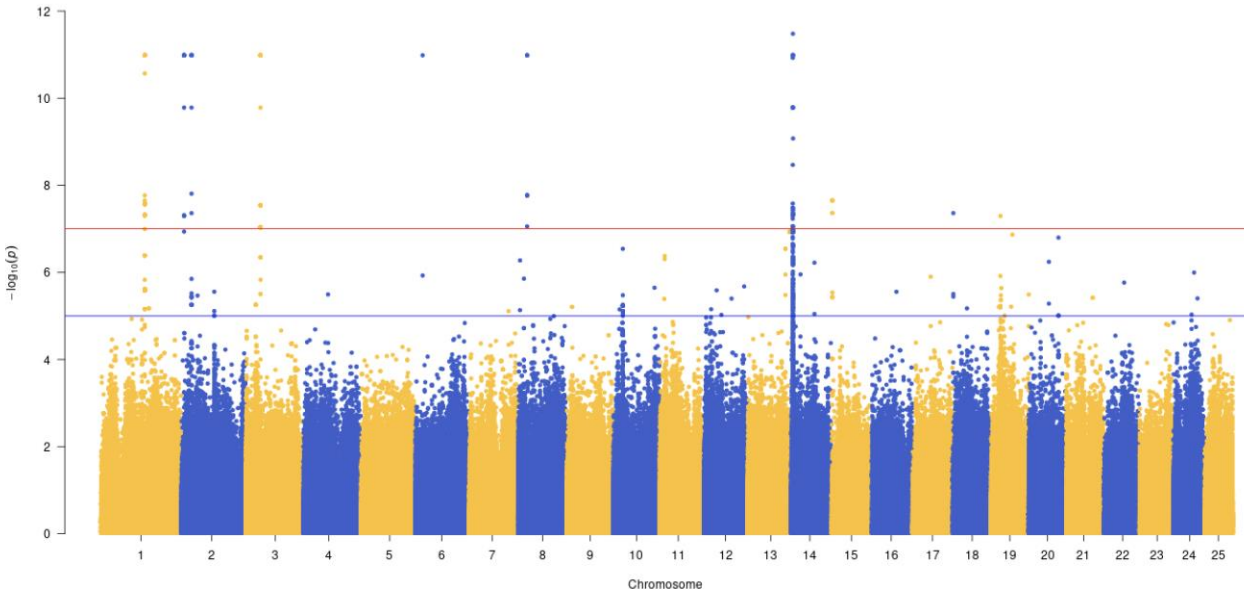

Figure 1. Manhattan figure demonstrating GWAS results between male and female individuals. The x- and y-axes represent localization in chromosomes and *p values*, respectively.

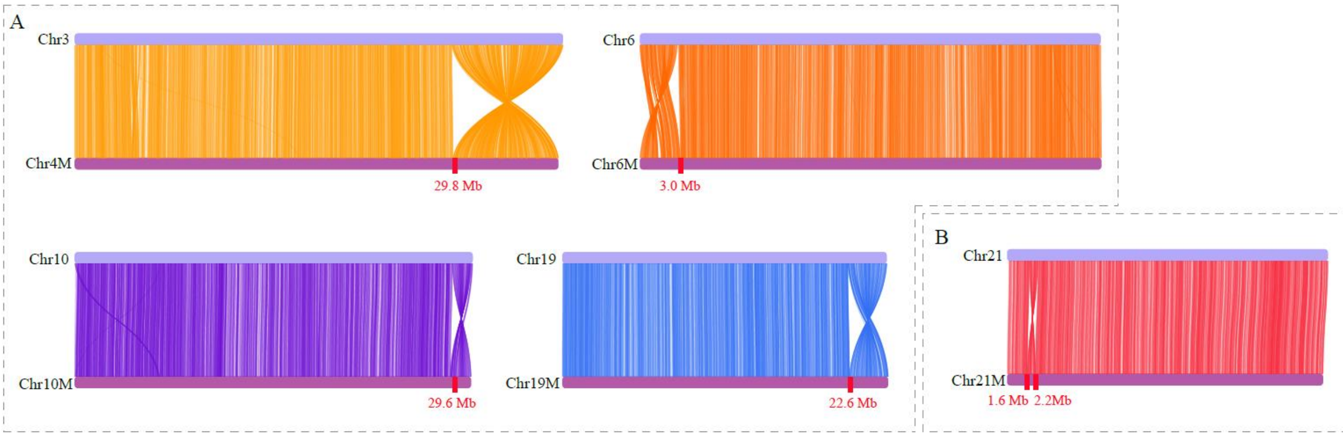

Figure 2. Chromosomal inversion events between male and female individuals. Chr3, Chr6, Chr10, Chr19 and Chr21 represent chromosomes of the female individual; Chr4M, Chr6M, Chr10M, Chr19M and Chr21M represent chromosomes of the male individual. (A) Four inversions in the terminal regions of chromosomes. (B) An inversion in the interior regions of Chr21 and Chr21M.

Table 1. Statistics of male and female genome assemblies.

	Female				Male			
	Scaffold		Contig		Scaffold		Contig	
	Length (bp)	Number	Length (bp)	Number	Length (bp)	Number	Length (bp)	Number
**Max length**	55,928,569		15,345,010		54,016,522		254,097	
**N50**	29,809,544	11	2,733,495	86	30,068,058	11	36,197	6,364
**N60**	27,447,229	14	2,158,000	119	27,527,535	14	28,504	8,785
**N70**	27,285,240	16	1,744,810	159	26,851,541	17	21,527	11,924
**N80**	25,552,712	19	1,179,287	213	25,375,165	20	14,934	16,239
**N90**	24,145,000	22	638,362	299	22,694,494	23	8,018	23,187
**Total_length**	781,489,634		780,969,649		789,196,668		779,505,746	
**Number>=0 bp**		749		1,796		149,334		192,358
**Number>=10000 bp**		428		1,346		48		20,837
**Number>=20000 bp**		228		1,018		25		12,809
**GC_rate**	0.4		0.4		0.4		0.4	
**BUSCO**	95.4% [S:92.4%, D:3.0%]				96.2% [S:93.4%, D:2.8%]			

Table 2. Predicted protein-coding genes in the male and female genomes.

Evidence	Method /Species	Female						Male					
		Numbers	Average gene length (bp)	Average CDS length (bp)	Average exon per gene	Average exon length (bp)	Average intron length (bp)	Numbers	Average gene length (bp)	Average CDS length (bp)	Average exon per gene	Average exon length (bp)	Average intron length (bp)
De novo	AUGUSTUS	53,315	7,324	968	5.0	193	1,579	57,273	6,765	910	4.7	193	1,573
Homolog	*Danio rerio*	21,631	11,497	1,600	8.7	185	1,292	20,444	12,279	1,641	8.8	187	1,370
	Gasterosteus aculeatus	25,581	9,030	1,264	7.2	177	1,263	25,123	9,321	1,271	7.1	179	1,319
	Oryzias latipes	18,480	11,895	1,773	8.9	198	1,275	18,080	12,513	1,704	8.7	195	1,397
	Takifugu rubripes	19,711	10,606	1,418	7.8	182	1,357	20,194	11,583	1,480	8.2	182	1,414
	Tetraodon nigroviridis	17,565	11,949	1,549	9.1	171	1,288	18,243	12,066	1,548	8.9	173	1,324
	Scleropages formosu	31,620	12,486	1,480	8.0	185	1,575	30,623	13,038	1,490	7.9	190	1,684
Total		25,328	12,358.42	1,584.44	9.4	167.78	1,276	25,244	12,775	1,609	9.4	172	1,337

References

1. Lake P, Bănărescu P and Banarescu P. Zoogeography of Fresh Waters. Volume 3. Distribution and Dispersal of Freshwater Animals in Africa, Pacific Areas and South America. Journal of the North American Benthological Society. 1996;15:265. doi:10.2307/1467954.

2. Mu X-d, Song H-m, Wang X-j, Yang Y-x, Luo D, Gu D-e, et al. Genetic variability of the Asian arowana, Scleropages formosus, based on mitochondrial DNA genes. Biochemical Systematics and Ecology. 2012;44:141–8. doi:10.1016/j.bse.2012.04.017.

3. Hilton E and Lavoué S. A review of the systematic biology of fossil and living bony-tongue fishes, Osteoglossomorpha (Actinopterygii: Teleostei). Neotropical Ichthyology. 2018;16 doi:10.1590/1982-0224-20180031.

4. Lavoué S. Testing a time hypothesis in the biogeography of the arowana genus Scleropages (Osteoglossidae). Journal of Biogeography. 2015;42 doi:10.1111/jbi.12585.

5. Greenwood PH, Rosen DE, Weitzman SH, Myers GS, History A and York N. Phyletic studies of teleostean fishes, with a provisional classification of living forms. XF2006174447. 1979;131.

6. Yue GH, Chang A, Alfiko Y and Suwanto A. Current Knowledge on the Biology and Aquaculture of the Endangered Asian Arowana. Reviews in Fisheries Science & Aquaculture. 2019;28:1-18. doi:10.1080/23308249.2019.1697641.

7. Yue GH, Ong D, Wong C, Lim L and Orbán L. A strain-specific and a sex-associated STS marker for Asian arowana (Scleropages formosus, Osteoglossidae). Aquaculture Research. 2003;34:951-7. doi:10.1046/j.1365-2109.2003.00949.x.

8. Shen XY, Kwan HY, Thevasagayam NM, Prakki SR, Kuznetsova IS, Ngoh SY, et al. The first transcriptome and genetic linkage map for Asian arowana. Mol Ecol Resour. 2014;14 3:622-35. doi:10.1111/1755-0998.12212.

9. Yue GH. Current status of genome sequencing and its applications in aquaculture. Aquaculture. 2017;468:337–47. doi:10.1016/j.aquaculture.2016.10.036.

10. Austin CM, Tan MH, Croft LJ, Hammer MP and Gan HM. Whole Genome Sequencing of the Asian Arowana (Scleropages formosus) Provides Insights into the Evolution of Ray-Finned Fishes. Genome Biol Evol. 2015;7 10:2885-95. doi:10.1093/gbe/evv186.

11. Bian C, Hu Y, Ravi V, Kuznetsova IS, Shen X, Mu X, et al. The Asian arowana (Scleropages formosus) genome provides new insights into the evolution of an early lineage of teleosts. Sci Rep. 2016;6:24501. doi:10.1038/srep24501.

12. Goodwin S, McPherson JD and McCombie WR. Coming of age: ten years of next-generation sequencing technologies. Nat Rev Genet. 2016;17 6:333-51. doi:10.1038/nrg.2016.49.

13. Jiao WB and Schneeberger K. The impact of third generation genomic technologies on plant genome assembly. Curr Opin Plant Biol. 2017;36:64-70. doi:10.1016/j.pbi.2017.02.002.

14. Chen Y, Chen Y, Shi C, Huang Z, Zhang Y, Li S, et al. SOAPnuke: a MapReduce acceleration-supported software for integrated quality control and preprocessing of high-throughput sequencing data. Gigascience. 2017;7 1:gix120.

15. Salmela L and Rivals E. LoRDEC: accurate and efficient long read error correction. Bioinformatics. 2014.

16. Song L, Bian C, Luo Y, Wang L, You X, Li J, et al. Draft genome of the Chinese mitten crab, Eriocheir sinensis. GigaScience,5,1(2016-01-28). 2016;5 1:5.

17. Kajitani R, Yoshimura D, Okuno M, Minakuchi Y, Kagoshima H, Fujiyama A, et al. Platanus-allee is a de novo haplotype assembler enabling a comprehensive access to divergent heterozygous regions. Nature communications. 2019;10 1:1-15.

18. Li H. Minimap2: pairwise alignment for nucleotide sequences. Bioinformatics. 2018; 18:18.

19. Vaser R, Sović I, Nagarajan N and Šikić M. Fast and accurate de novo genome assembly from long uncorrected reads. Genome research. 2017;27 5:737-46. doi:10.1101/gr.214270.116.

20. Walker BJ, Abeel T, Shea T, Priest M, Abouelliel A, Sakthikumar S, et al. Pilon: an integrated tool for comprehensive microbial variant detection and genome assembly improvement. PloS one. 2014;9 11:e112963.

21. Boetzer M and Pirovano W. SSPACE-LongRead: scaffolding bacterial draft genomes using long read sequence information. Bmc Bioinformatics. 2014;15.

22. Boetzer M, Henkel CV, Jansen HJ, Butler D and Pirovano W. Scaffolding pre-assembled contigs using SSPACE. Bioinformatics. 2011;27 4:578-9.

23. Durand NC, Shamim MS, Machol I, Rao SS, Huntley MH, Lander ES, et al. Juicer provides a one-click system for analyzing loop-resolution Hi-C experiments. Cell systems. 2016;3 1:95-8.

24. Dudchenko O, Batra SS, Omer AD, Nyquist SK, Hoeger M, Durand NC, et al. De novo assembly of the Aedes aegypti genome using Hi-C yields chromosome-length scaffolds. Science. 2017;356 6333:92-5.

25. Luo R, Liu B, Xie Y, Li Z and Liu Y. SOAPdenovo2: an empirically improved memory-efficient short-read de novo assembler. GigaScience. 2012;1.

26. Smit A, Hubley R and Green P. RepeatMasker Open-4.0. 2013-2015< http://www. repeatmasker. org>. 2019.

27. Bao W, Kojima KK and Kohany O. Repbase Update, a database of repetitive elements in eukaryotic genomes. BioMed Central % Journal Article; 2015.

28. Zhao X and Hao W. LTR_FINDER: an efficient tool for the prediction of full-length LTR retrotransposons. Nucleic Acids Research. 2007;35 Web Server issue:W265-8.

29. Benson G. Tandem repeats finder: a program to analyze DNA sequences. Oxford University Press % Journal Article; 1999.

30. Bruna T, Hoff KJ, Lomsadze A, Stanke M and Borodovsky M. BRAKER2: automatic eukaryotic genome annotation with GeneMark-EP+ and AUGUSTUS supported by a protein database. NAR Genom Bioinform. 2021;3 1:lqaa108. doi:10.1093/nargab/lqaa108.

31. Bian C, Hu Y, Ravi V, Kuznetsova IS, Shen X, Mu X, et al. The Asian arowana (Scleropages formosus) genome provides new insights into the evolution of an early lineage of teleosts. Scientific reports. 2016;6 1:1-17.

32. Kanehisa M, Furumichi M, Tanabe M, Sato Y and Morishima K. KEGG: new perspectives on genomes, pathways, diseases and drugs. Nucleic Acids Res. 2017;45 D1:D353-d61. doi:10.1093/nar/gkw1092.

33. Bairoch A, Apweiler R, Wu CH, Barker WC, Boeckmann B, Ferro S, et al. The universal protein resource (UniProt). Nucleic acids research. 2005;33 suppl_1:D154-D9.

34. Jones P, Binns D, Chang HY, Fraser M, Li W, McAnulla C, et al. InterProScan 5: genome-scale protein function classification. Bioinformatics. 2014;30 9:1236-40. doi:10.1093/bioinformatics/btu031.

35. Zdobnov EM and Apweiler R. InterProScan–an integration platform for the signature-recognition methods in InterPro. Bioinformatics. 2001;17 9:847-8.

36. Thomas PD, Campbell MJ, Kejariwal A, Mi H, Karlak B, Daverman R, et al. PANTHER: a library of protein families and subfamilies indexed by function. Genome Res. 2003;13 9:2129-41. doi:10.1101/gr.772403.

37. Bateman A, Coin L, Durbin R, Finn RD, Hollich V, Griffiths-Jones S, et al. The Pfam protein families database. Nucleic acids research. 2004;32 suppl_1:D138-D41.

38. Attwood TK, Croning MDR, Flower DR, Lewis AP, Mabey JE, Scordis P, et al. PRINTS-S: the database formerly known as PRINTS. Nucleic Acids Research. 2000;28 1:225-7. doi:10.1093/nar/28.1.225 %J Nucleic Acids Research.

39. Servant F, Bru C, Carrère S, Courcelle E, Gouzy J, Peyruc D, et al. ProDom: automated clustering of homologous domains. Brief Bioinform. 2002;3 3:246-51. doi:10.1093/bib/3.3.246.

40. Sigrist CJ, Cerutti L, de Castro E, Langendijk-Genevaux PS, Bulliard V, Bairoch A, et al. PROSITE, a protein domain database for functional characterization and annotation. Nucleic Acids Res. 2010;38 Database issue:D161-6. doi:10.1093/nar/gkp885.

41. Letunic I, Doerks T and Bork P. SMART 7: recent updates to the protein domain annotation resource. Nucleic Acids Res. 2012;40 Database issue:D302-5. doi:10.1093/nar/gkr931.

42. Ashburner M, Ball CA, Blake JA, Botstein D, Butler H, Cherry JM, et al. Gene ontology: tool for the unification of biology. Nature genetics. 2000;25 1:25-9.

43. Kim D, Langmead B and Salzberg SL. HISAT: a fast spliced aligner with low memory requirements. Nature methods. 2015;12 4:357-60. doi:10.1038/nmeth.3317.

44. Trapnell C, Hendrickson DG, Sauvageau M, Goff L, Rinn JL and Pachter L. Differential analysis of gene regulation at transcript resolution with RNA-seq. Nature biotechnology. 2013;31 1:46-53. doi:10.1038/nbt.2450.

45. Robinson MD, McCarthy DJ and Smyth GK. edgeR: a Bioconductor package for differential expression analysis of digital gene expression data. Bioinformatics. 2010;26 1:139-40. doi:10.1093/bioinformatics/btp616.

46. Li H and Durbin R. Fast and accurate short read alignment with Burrows-Wheeler transform. Bioinformatics. 2009;25 14:1754-60. doi:10.1093/bioinformatics/btp324.

47. Mckenna A, Hanna M, Banks E, Sivachenko A, Cibulskis K, Kernytsky A, et al. The Genome Analysis Toolkit: a MapReduce framework for analyzing next-generation DNA sequencing data. Genome Research. 2010;20 9:1297-303.

48. Kang HM, Sul JH, Service SK, Zaitlen NA, Kong S-y, Freimer NB, et al. Variance component model to account for sample structure in genome-wide association studies. Nature genetics. 2010;42 4:348-54.

49. Purcell S, Neale B, Todd-Brown K, Thomas L, Ferreira MA, Bender D, et al. PLINK: a tool set for whole-genome association and population-based linkage analyses. The American journal of human genetics. 2007;81 3:559-75.

50. Turner SD. qqman: an R package for visualizing GWAS results using QQ and manhattan plots. Biorxiv. 2014:005165.

51. Marçais G, Delcher AL, Phillippy AM, Coston R, Salzberg SL and Zimin AJPcb. MUMmer4: A fast and versatile genome alignment system. 2018;14 1:e1005944.

52. Dougherty GW, Mizuno K, Nothe-Menchen T, Ikawa Y, Boldt K, Ta-Shma A, et al. CFAP45 deficiency causes situs abnormalities and asthenospermia by disrupting an axonemal adenine nucleotide homeostasis module. Nat Commun. 2020;11 1:5520. doi:10.1038/s41467-020-19113-0.

Manuscript

Click here to access/download;Manuscript;Supplementary Material 20220224.docx

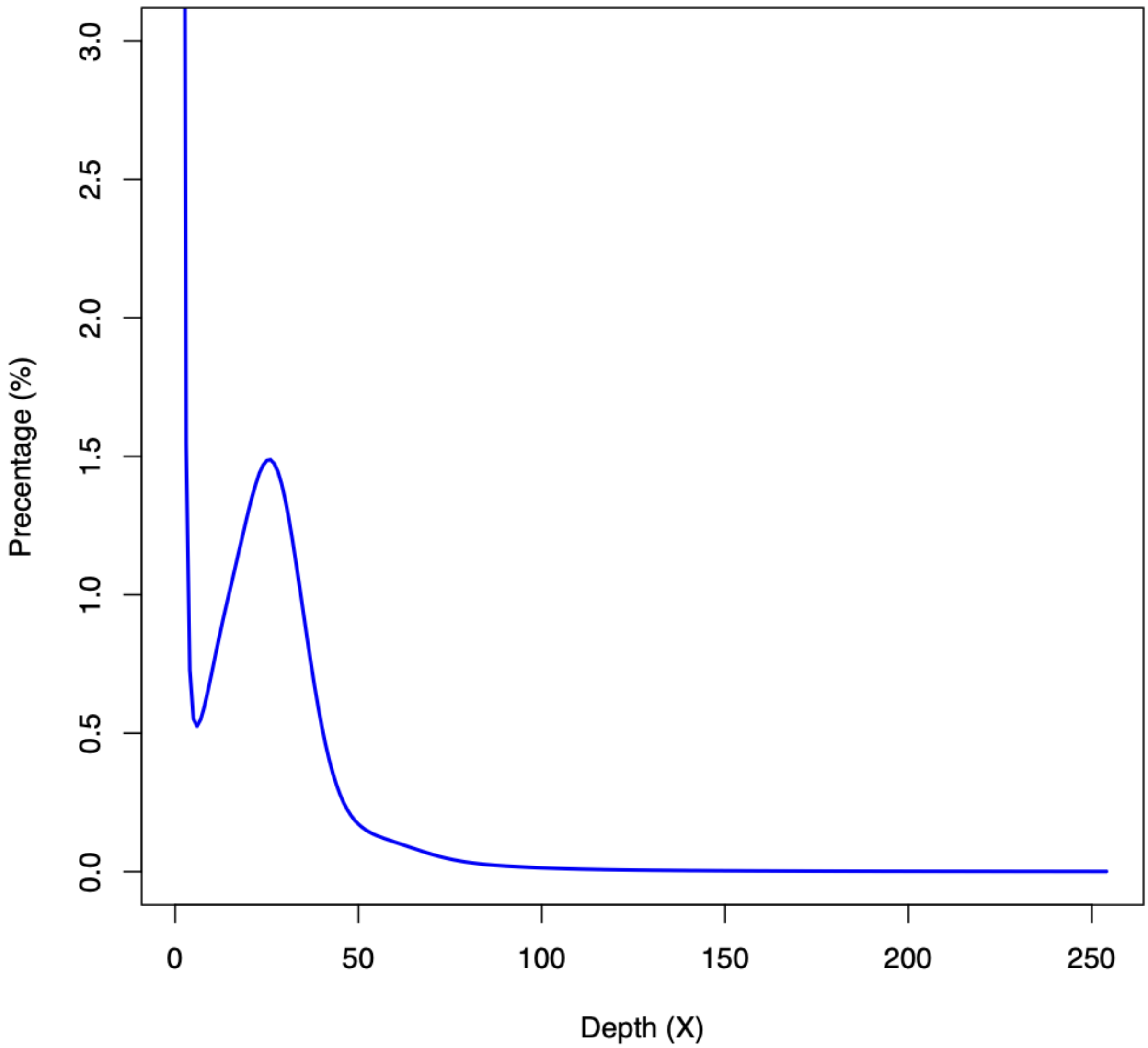

Supplementary Figure 1. 17-kmer analysis for prediction of genome size of the female individual. Its genome size was estimated at 0.82 Gb.

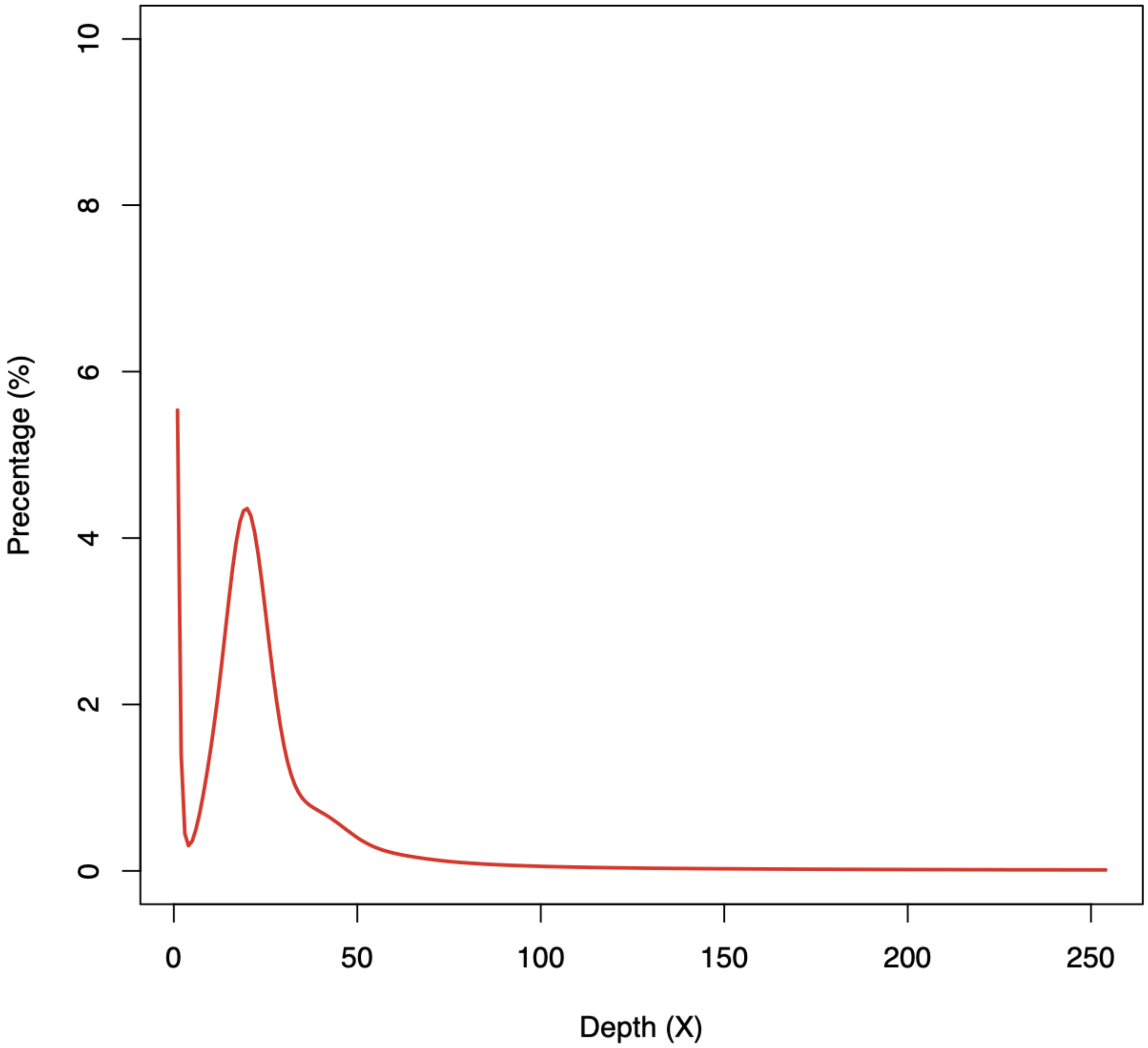

Supplementary Figure 2. 17-kmer analysis for prediction of genome size of the male individual. Its genome size was estimated at 0.85 Gb.

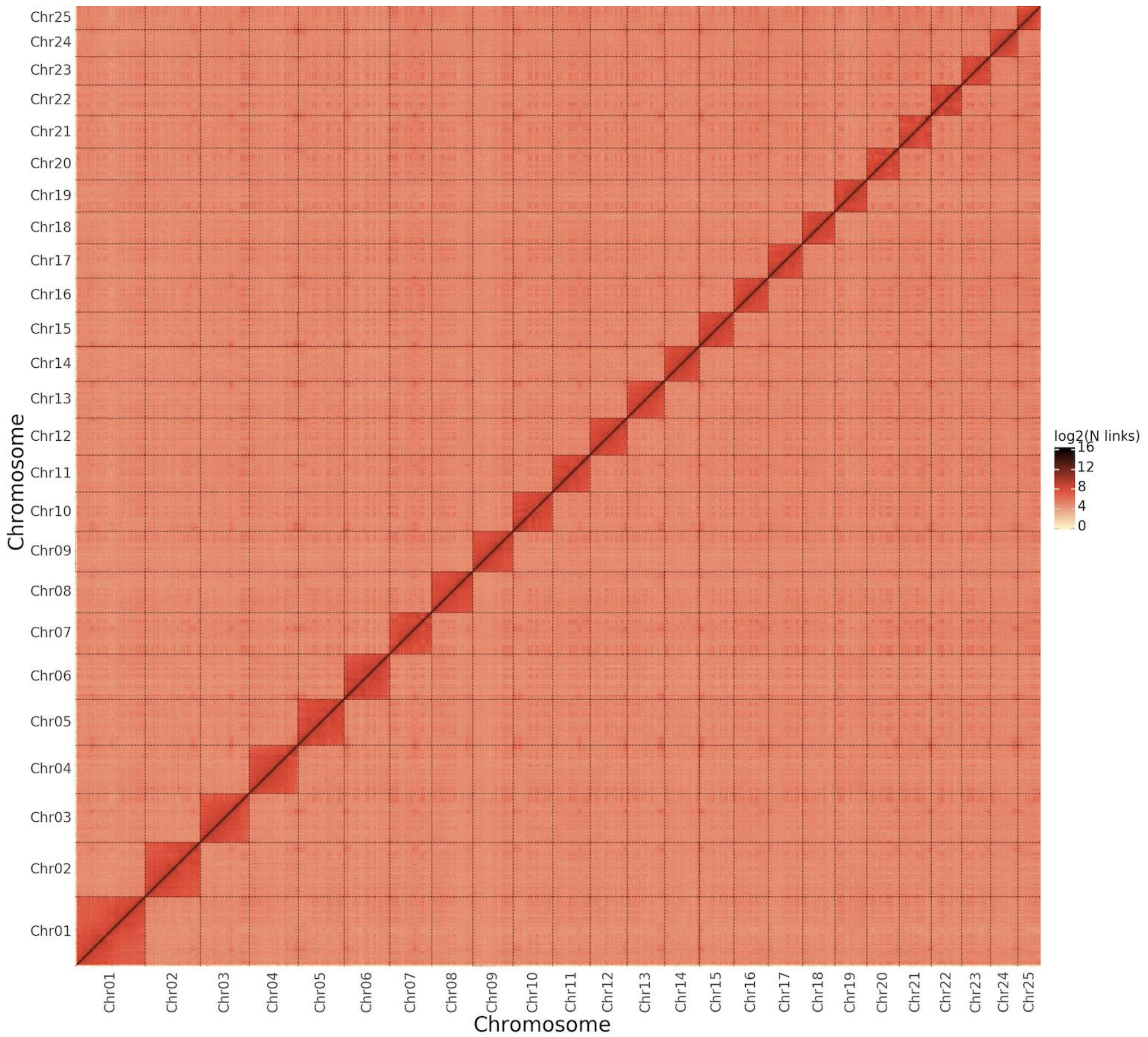

Supplementary Figure 3. Heatmap of the Hi-C result of female individual.

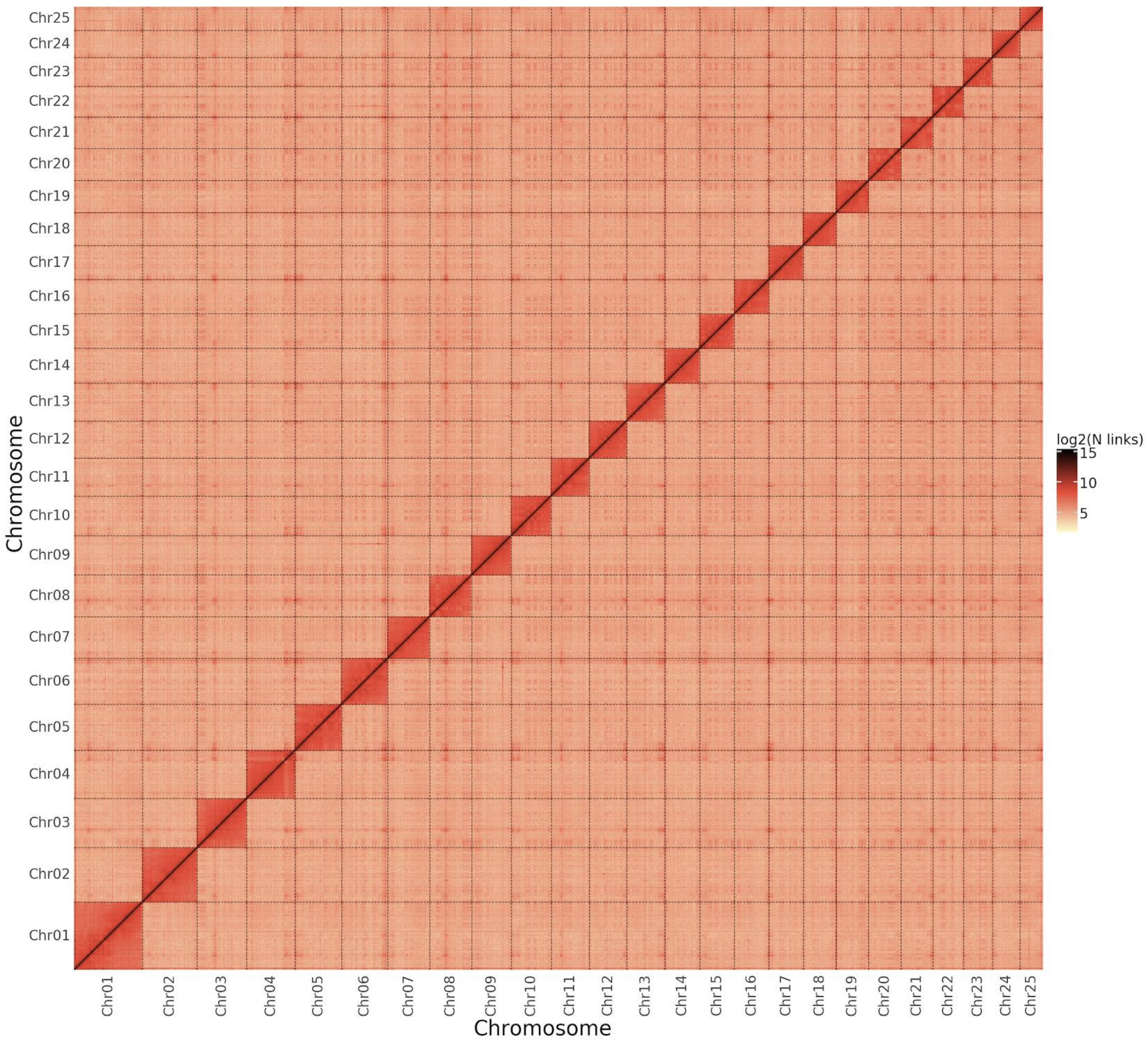

Supplementary Figure 4. Heatmap of the Hi-C result of male individual.

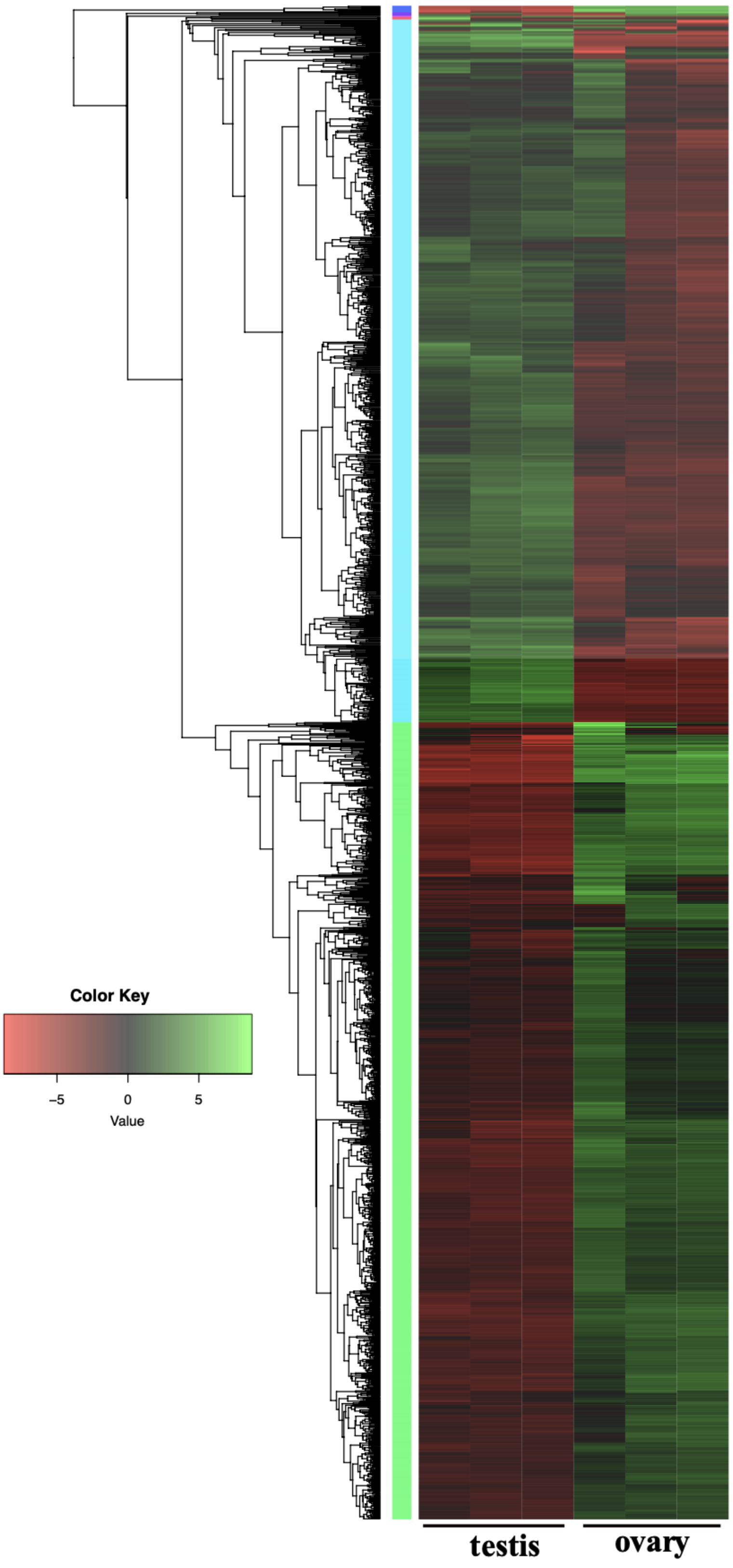

Supplementary Figure 5. A heatmap of DEGs in the testis and ovary tissues of three male and three female individuals. Y-axis refers to clusters of the DEGs. Colour key indicates the intensity associated with normalized transcription values.

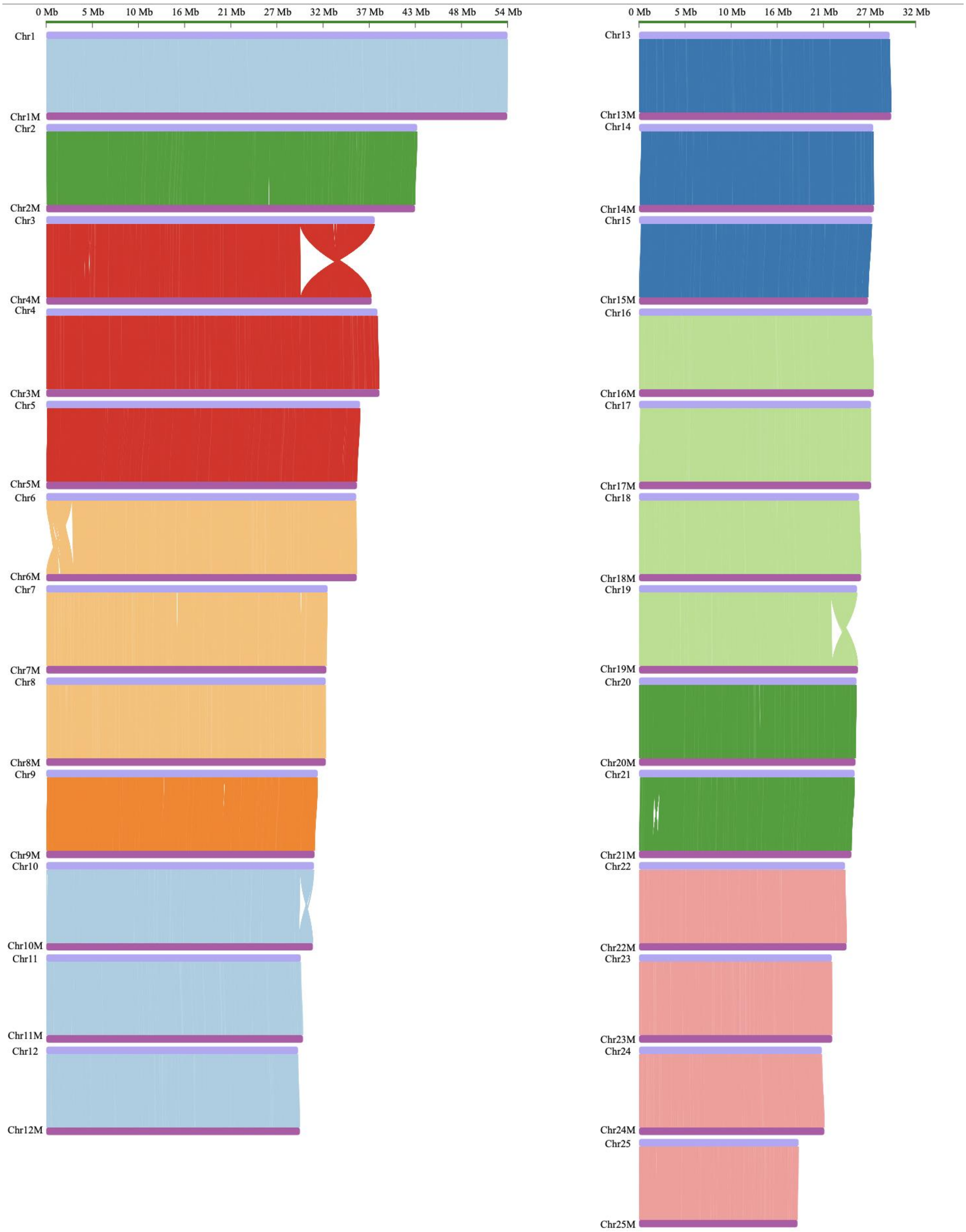

Supplementary Figure 6. Chromosomal alignments of male and female chromosomes.

Supplementary Table 1. Summary of sequenced reads for male and female genomes.

**Sex**	**Sequence platform**	**Insert size**	**Raw bases (Gb)**
Female	Pacbio	20k	27.4
	Illumina	170bp	19.7
		500bp	16.1
		800bp	9.6
		2k	15.5
		5k	6.0
		10k	4.7
		20k	1.8
Female HiC	Illumina	-	80.7
Male	Illumina	170bp	63.1
		500bp	48.9
		800bp	20.1
		2k	43.8
		5k	46.1
		10k	28.6
		20k	43.8

Supplementary Table 2. Summary of map ratio for the 30 male and female samples.

Libraries	Raw bases (Gb)	Raw depth (X)	Mapped bases (Gb)	Mapped ratio (%)	Sex
wHAXPI043368-76	22.9	29.2	19.0	83.2	female
wHAXPI043369-81	18.9	24.2	16.1	85.2	female
wHAXPI043373-75	25.1	32.1	21.4	85.2	female
wHAXPI043374-78	24.4	31.2	20.6	84.4	female
wHAXPI043378-80	22.1	28.2	18.5	84.0	female
wHAXPI043382-85	19.6	25.1	16.7	85.2	female
wHAXPI046607-85	24.4	31.2	20.2	82.9	female
wHAXPI046620-84	26.2	33.5	22.0	83.9	female
wHAXPI047283-16	22.9	29.4	20.2	88.0	female
wHAXPI047286-24	25.9	33.2	22.2	85.5	female
wHAXPI047309-11	21.4	27.4	18.8	87.9	female
wHAXPI047311-30	23.5	30.1	20.3	86.3	female
wHAXPI047315-23	29.6	37.9	25.6	86.4	female
wHAXPI047319-32	23.1	29.6	20.0	86.7	female
wHAXPI046644-83	25.5	32.7	21.5	84.3	female
wHAXPI043365-83	21.1	27.0	18.0	85.5	male
wHAXPI043366-84	20.6	26.3	17.3	84.2	male
wHAXPI043376-79	19.3	24.6	16.3	84.6	male
wHAXPI043381-86	20.4	26.0	17.2	84.4	male
wHAXPI043383-77	23.9	30.5	19.6	82.1	male
wHAXPI043384-87	23.4	30.0	20.4	87.1	male
wHAXPI047290-26	23.5	30.1	20.1	85.7	male
wHAXPI047292-17	24.2	31.0	21.2	87.5	male
wHAXPI047299-18	22.3	28.6	19.5	87.5	male
wHAXPI047304-21	25.5	32.6	22.0	86.3	male
wHAXPI047305-13	28.0	35.9	24.0	85.5	male
wHAXPI047306-8	22.1	28.3	19.2	86.7	male
wHAXPI047307-14	28.2	36.1	24.8	87.9	male
wHAXPI047316-12	25.3	32.3	21.9	86.7	male
wHAXPI047320-9	25.9	33.2	21.1	81.4	male

Supplementary Table 3. Repetitive elements in the assembled genome of female individual.

Type	Repbase TEs		TE proteins		*De novo*		Combined TEs	
	Length (bp)	% in genome	Length (bp)	% in genome	Length (bp)	% in genome	Length (bp)	% in genome
DNA	42,384,960	5.4	599,916	0.1	40,713,941	5.2	69,425,706	8.9
LINE	32,749,014	4.2	25,288,682	3.2	121,407,716	15.5	129,160,932	16.5
SINE	17,543,728	2.2	0	0.0	5,532,654	0.7	22,222,630	2.8
LTR	11,980,146	1.5	9,903,737	1.3	90,664,834	11.6	93,640,806	12.0
Other	11,678	0.0	0	0.0	0	0.0	11,678	0.0
Unknown	0	0.0	0	0.0	1,824,263	0.2	1,824,263	0.2
Total	94,068,254	12.0	35,776,743	4.6	209,111,564	26.8	217,364,929	27.8

Supplementary Table 4. Repetitive elements in the assembled genome of male individual.

Type	Repbase TEs		TE proteins		*De novo*		Combined TEs	
	Length (bp)	% in genome	Length (bp)	% in genome	Length (bp)	% in genome	Length (bp)	% in genome
DNA	39,739,174	5.0	2,191,643	0.3	67,955,759	8.6	90,304,208	11.4
LINE	27,217,668	3.5	21,943,822	2.8	95,249,961	12.1	104,720,326	13.3
SINE	10,675,147	1.4	0	0.0	11,033,130	1.4	20,764,835	2.6
LTR	10,484,227	1.3	8,436,744	1.1	85,877,038	10.9	89,054,204	11.3
Other	15,277	0.0	0	0.0	0	0.0	15,277	0.0
Unknown	0	0.0	0	0.0	1,450,089	0.2	1,450,089	0.2
Total	78,180,628	9.9	32,556,483	4.1	202,768,263	25.7	210,089,397	26.6

Supplementary Table 5. Chromosome location of SNPs.

			CDS			
Chromosome	Total	Intergenic	Total	Synonymous	Non-Synonymous	Intron
Chr1	653,412	398,298	17,312	10,694	6,618	237,802
Chr2	522,180	316,604	12,801	7,770	5,031	192,775
Chr3	472,925	284,509	22,925	6,817	16,108	165,491
Chr4	434,120	252,430	23,863	6,778	17,085	157,827
Chr5	447,562	271,570	9,868	6,133	3,735	166,124
Chr6	461,273	281,617	13,025	7,519	5,506	166,631
Chr7	345,183	201,302	11,042	6,556	4,486	132,839
Chr8	397,110	243,146	11,462	6,672	4,790	142,502
Chr9	383,554	232,414	10,063	5,729	4,334	141,077
Chr10	360,838	225,310	10,641	6,272	4,369	124,887
Chr11	342,505	208,663	8,894	5,273	3,621	124,948
Chr12	349,640	219,690	9,353	5,627	3,726	120,597
Chr13	346,090	233,607	8,784	5,051	3,733	103,699
Chr14	311,621	208,702	7,097	4,348	2,749	95,822
Chr15	296,890	186,683	7,466	4,376	3,090	102,741
Chr16	290,725	169,449	9,257	5,400	3,857	112,019
Chr17	306,448	182,399	7,547	4,586	2,961	116,502
Chr18	302,609	187,776	6,944	4,178	2,766	107,889
Chr19	332,470	195,662	9,211	5,541	3,670	127,597
Chr20	295,685	172,571	9,929	5,719	4,210	113,185
Chr21	277,062	163,698	10,512	5,244	5,268	102,852
Chr22	273,376	162,500	7,023	4,333	2,690	103,853
Chr23	255,368	158,502	7,876	4,672	3,204	88,990
Chr24	242,201	143,980	5,848	3,612	2,236	92,373
Chr25	217,912	130,825	6,276	3,746	2,530	80,811
Total	8,918,759	5,431,907	265,019	142,646	122,373	3,221,833

Supplementary Table 6. Genes in potential sex divergence regions in chromosomes predicted by the GWAS and their expression values in ovary and testis tissues.

Gene ID	Chr	Gene symbol	Ovary1	Ovary2	Ovary3	Testis1	Testis2	Testis3
jg33400.t1	Chr12	*ncam2*	0.03	0.04	0.01	0.14	0.03	0.04
jg33600.t1	Chr14	*cd48*	0	0.02	0	0	0.04	0.11
jg43012.t1	Chr18	*wscd2*	0	0.04	0	0.51	0.43	0.74
jg13369.t1	Chr19	*cfap52*	0.25	0.28	0.51	25.39	25.06	6.78
jg13247.t1	Chr19	*cep95*	5.50	18.40	16.74	2.75	2.81	4.34
jg13148.t1	Chr19	*ca10*	0	0.024	0	0.48	0.32	0.38
jg21466.t1	Chr19	*mrtfb*	0.26	1.46	1.18	2.21	3.32	2.05
jg11085.t1	Chr21	*itga4*	0.12	0.11	0.06	0.52	0.54	0.32

Supplementary Table 7. Statistics of the mapped ratio of male and female chromosomes.

Chromosome of Female	The mapped regions/chromosome length	Chromosome of male	The mapped regions/chromosome length
Chr1	0.96	Chr1M	0.96
Chr2	0.95	Chr2M	0.96
Chr3	0.91	Chr4M	0.95
Chr4	0.98	Chr3M	0.98
Chr5	0.95	Chr5M	0.96
Chr6	0.95	Chr6M	0.95
Chr7	0.97	Chr7M	0.97
Chr8	0.97	Chr8M	0.97
Chr9	0.96	Chr9M	0.97
Chr10	0.96	Chr10M	0.96
Chr11	0.97	Chr11M	0.96
Chr12	0.97	Chr12M	0.97
Chr13	0.96	Chr13M	0.96
Chr14	0.96	Chr14M	0.96
Chr15	0.95	Chr15M	0.96
Chr16	0.98	Chr16M	0.97
Chr17	0.97	Chr17M	0.97
Chr18	0.98	Chr18M	0.97
Chr19	0.97	Chr19M	0.97
Chr20	0.95	Chr20M	0.96
Chr21	0.96	Chr21M	0.97
Chr22	0.98	Chr22M	0.97
Chr23	0.97	Chr23M	0.96
Chr24	0.98	Chr24M	0.97
Chr25	0.96	Chr25M	0.97

Click here to access/download
Supplementary Material
Supplementary Material.docx
