# Supplementary material for: Identification of candidate sex‐specific genomic regions in male and female Asian arowana genomes
Source: Gigascience. 2022 Sep 15;11:giac085. doi: 10.1093/gigascience/giac085 (PMC9475665; doi:10.1093/gigascience/giac085)
Supplement: giac085_GIGA-D-22-00043_Revision_1 [file giac085_giga-d-22-00043_revision_1.pdf]

GigaScience

Identification of candidate sex-specific genomic regions in male and female Asian arowana genomes

--Manuscript Draft--

Manuscript Number:	GIGA-D-22-00043R1
Full Title:	Identification of candidate sex-specific genomic regions in male and female Asian arowana genomes
Article Type:	Data Note
Funding Information:	Guangdong Provincial Special Fund for Modern Agriculture Industry Technology Innovation Team (2022KJ150) — Professor Xidong Mu
Abstract:	Background: Asian arowana, Scleropages formosus, is one of the most expensive aquarium fish species worldwide. Its sex, however, cannot be distinguished clearly at any development stage, which impedes captive breeding and species protection for this endangered aquarium fish.<br>Results: To discover molecular clues to the sex of Asian arowana, we sequenced 26.5 Gb of PacBio HiFi reads and 179.2 Gb of Hi-C reads for one male fish, and also sequenced 106.5 Gb of Illumina reads, 36.0 Gb of PacBio Sequel reads and 80.7 Gb of Hi-C reads for one female individual. The final male and female genome assemblies were approximately 756.8 Mb and 781.5 Mb in length and contained 25,262 and 25,328 protein-coding genes respectively. We also resequenced the genomes of 15 male and 15 female individuals with approximately 722.1 Gb of Illumina reads. A genome-wide association study (GWAS) identified several potentially divergent regions between male and female individuals. In these regions, cd48 and cfap52 could be candidate genes for sex determination of Asian arowana. We also found some structural variations in few chromosomes between male and female individuals.<br>Conclusion: We provided an improved reference genome assembly of female arowana and generated the first sequenced genome of one male individual.These valuable genetic resources and resequencing data may improve global aquarium fish research.<br>Key words: Asian arowana; male and female; genome sequencing and resequencing; sex-related genes
Corresponding Author:	Chao Bian<br><br>CHINA
Corresponding Author Secondary Information:	
Corresponding Author's Institution:	
Corresponding Author's Secondary Institution:	
First Author:	Chao Bian
First Author Secondary Information:	
Order of Authors:	Chao Bian<br>Xidong Mu<br>Yi Liu<br>Chao Liu<br>Chenxi Zhao<br>Ruihan Li<br>Xinxin You<br>Yexin Yang

Powered by Editorial Manager® and ProduXion Manager® from Aries Systems Corporation

	Xuejie Wang
	Yinchang Hu
	Qiong Shi
**Order of Authors Secondary Information:**	
**Response to Reviewers:**	Dear editor,<br>Thanks for your nice advice and the two reviewers' comments. According to your instructive opinions, we sequenced additional PacBio HiFI and Hi-C reads for the male arowana, and then performed a series of new analyses. We also made careful revisions throughout the manuscript. Our revised manuscript (changes are highlighted in yellow) and point-by-point responses are uploaded for your consideration.<br><br><br>Reviewer #1:<br>Title: Sex-specific genomic region identification in male and female Asian arowana genomes<br>SUMMARY<br>This manuscript presents a genomics analysis of a fish species, Scleropages formosus, for which there was only a draft genome previously. The authors present a high-quality female genome assembly and lower quality male genome, plus gene annotation, repeats analysis, BUSCO, transcriptomics, GWAS, and comparative genomics. These data helped identify some candidates for the sex-specific regions which remain elusive.<br>MAJOR ISSUE<br>The paper says the annotation files are available at NCBI. However, the NCBI pages say, "No public data is linked to this project." Of course, the assembly and the annotation must be released prior to publication.<br>Response: Thanks for your instructive comments. Yes, the genome data of both female and male individuals were released from the NCBI. They are accessible at https://www.ncbi.nlm.nih.gov/bioproject/?term=PRJNA810746 and https://www.ncbi.nlm.nih.gov/bioproject/?term=PRJNA810753.<br>Related annotation files were uploaded to the CNSA: https://ftp.cngb.org/pub/CNSA/data2/CNP0002889/CNS0538329/CNA0047397/.<br>Sequenced reads were also deposited at the CNSA for public availability: https://db.cngb.org/search/organism/113540/.<br><br>MINOR MODIFICATIONS<br>The experiment was well-designed and the presentation is complete. However, some aspects of the presentation should be clarified.<br>1. The paper must make clear that the sex-specific region was not found. The current title is ambiguous about this, and it led me to believe the region had been found. Line 296 says the discovered differences may cause sex divergence, but the paper should note that the true sex-determining region may not be among the candidates identified here.<br>Response: You are right. We changed the title to "Identification of candidate sex-specific genomic regions in male and female Asian arowana genomes" (lines 1-2).<br><br>2. The male assembly is a short-read assembly that was made chromosome-length using female Hi-C. Thus, long-range connectivity in the male assembly is partially derived from female fish. This is indicated in the Methods, but the analysis should note this limitation. For example, line 239 should specify, "female Hi-C". The analysis of chromosome rearrangements in Figures 2 and S6 could note that some rearrangements may be missing in the "femalized" male assembly.<br>Response: Thanks for your nice comments. We additionally sequenced the male individual by using PacBio HiFi and Hi-C platforms. Therefore, we obtained a new high-quality male genome assembly. We performed a solid synteny analysis to compare the new chromosome-level male genome assembly to the original female assembly again; interestingly, we identified three same rearrangements as in previous results between male and female individuals. We therefore think that these rearrangements could be truthfully existed with more confidence. More detailed software parameters were added in the revised manuscript, and related read data were uploaded and released for public availability to make our results maximumly reproductive by other researchers.

3. The assembly pipeline needs clarification. The existing description in Methods is technically correct but potentially confusing for some readers. Add sentences or a figure describing the male and female pipelines from sequencing to assembly. Make clear the hybrid assembly process is not experimental i.e. it carefully follows processes that were previously published. For female, make clear it was polished with PacBio and polished again with Illumina. Describe the DBG2OLC hybrid step in its own sentence. State the value of K (K=17) near the formula that uses it. State how the Illumina data was "filtered."
Response: Thanks for your instructive advice and comments. Yes, we added a figure (as Supplementary Figure 3) to summarize the detailed assembling pipelines for female and male assemblies, making the assembly steps for both female and male genomes more clearly.

We added a sentence to introduce some previous studies that employed the similar hybrid assembly approach as follows in lines 147-149:
This hybrid assembly pipeline was well performed in many previous studies [20, 26, 27].

We revised the formula as follows (lines 133-135): G = N*(L–k-mer + 1)/K_depth, where k-mer length is defined as 17 bp, N is the total number of reads, and K_depth represents the frequency of occurrence more frequently than others.

For the Illumina data filtering, we revised this sentence as follows (line 129):
After filtering adapter sequences and low-quality reads by SOAPnuke v.1.5.6 with detailed parameters: filter -l 10 -q 0.1 -n 0.01 (RRID:SCR_015025) [14].

4. The Abstract says "an improved [female] reference" is presented, but the new assembly was not explicitly compared to the old ones. The text only implies (lines 80-84) that the old ones had shorter scaffolds and more gaps. It would improve the paper to include the assembly comparison. Does the new assembly contain more genes, more repeats, etc.? If such additional analysis is impossible, the abstract needs modification. The Introduction's off-hand remark that this species has been omitted from key studies due to poor quality of the previous assemblies (lines 85-86) must be justified or removed.
Response: Thanks for your nice advice. In fact, we compared the contig N50 values, gene numbers and repeat contents between this new female genome assembly and our previous one; the contig N50 of the new one was about 90 times more contiguous than the previous one. We also predicted about 3,000 more genes in this new female genome, although the repeats are similar in both assemblies. Related sentences were added in lines 250-255, 275-277 and 268-269.

5. Some unscientific language should be changed. The fish should not be subjectively called "famous" and "beautiful" in a science journal. (But perhaps a poem about the fish could be quoted?) Lines 59-60 claim that the fish is a "symbol of lucky … particularly in Asian countries"; the reference to a geography study leaves the claim unsupported so I think the whole sentence should be cut. The word "remarkable" is inappropriate to describe structural variation: lines 32 and 304. The abstract should say these assemblies "may" aid research, not "will" aid research (line 39). The statement that the assembly covers 98% of the genome (line 236) should clarify the comparison is to the genome size estimated from read coverage. The word "significant" must be changed or backed by statistics: line 290.
Response: Thanks for your instructive advice. Yes, we removed these words like “beautiful”, “symbol of lucky”, and ‘famous’ (in the abstract).

We changed ‘remarkable’ and ‘remarkably’ to ‘potential’ and ‘potentially’, and revised ‘will’ to ‘may’ in lines 34, 323, and 40 respectively.

For “98% of the genome”, we revised this sentence in lines 250-252 as follows:
The final assembly of the female individual consisted of 25 chromosomes and covered 765.8 Mb, which accounts for 98.0% of the assembled scaffolds.

We also changed the ‘significant’ to ‘potential’ in line 309.

Powered by Editorial Manager® and ProduXion Manager® from Aries Systems Corporation

6. Enhance the figure captions. Why does female chr 3 aligns to male chr 4? Why are alignments grouped by color in Figure S6? What is shown by the middle vertical color bar in Figure S5? What are the areas under the curves in key regions of the coverage plots in Figures S1 and S2? Perhaps insert a zoom-in histogram of the critical region so readers see each integer coverage level.
Response: Thanks for your good advice. Yes, we performed new genome-wide alignment and revised Figure 2 and Supplementary Figure 7, after we sequenced new HiFi and Hi-C data of the male and constructed a new male chromosome-level assembly.

The colors in Figure S6 were randomly selected by the RectChr software. For the Figure S5, color key indicates log2-transformed transcription values; we added this description in the Supplementary Figure 6 (original Figure S5).

We supplemented the peak coverage of the kmer curves in captions of the Figures S1 and S2. For the integer coverage level of each inversion boundary (also known as breakpoint), we first applied the minimap2 with default parameters to align PacBio reads to the male genome assembly, and then used the Integrative Genomics Viewer software to show the detailed read coverage of the critical regions in Supplementary Figure 8.

RECOMMENDATIONS
This manuscript aligns well with the journal's instruction for authors that says, "One of the aims of a Data Note is to incentivize and more rapidly release data before subsequent detailed analysis has been carried out." However, the authors may want to improve this paper by analyzing some additional aspects of their data.
1. Address the male/female assembly quality difference. The paper says "both genome assemblies are indeed of high quality and completeness" based on BUSCO: line 246. However, the paper has indications of a difference in quality: the male had lower resequencing map rate (line 264) and lower repeat content (line 250). Provide the male contig N50, not just scaffold N50 (line 239). How would results have changed if you assembled the female from Illumina-only, as was done for male? What was gained from the additional resources invested in the female assembly?
Response: Thanks for your good questions and comments. In fact, we additionally sequenced PacBio HiFi and Hi-C data of the male individual, and obtained a new chromosome-level genome assembly for the male arowana. In this way, both male and female genomes are of high quality. We also compared the new male genome assembly with the female assembly, which confirms three same rearrangements as in previous results.

2. When female Hi-C was applied to male contigs, were any contig breaks suggested?
Response: We additionally sequenced Hi-C data of the male individual, and assembled a new chromosome-level genome assembly for the male arowana.

3. Of the intersex chromosome rearrangements found, what genes are nearby?
Response: We revised the Figure 2 and added detailed gene ids and functional descriptions near the inversion boundaries.

Reviewer #2:
This paper describes two new genome assemblies, as well as two experimental datasets, all aimed at understanding sex determination in the Asian arowana. In general, the data and methods used are well suited for addressing this issue.
There are a few issues I would like to ask the authors to comment on:
1. The methods for both genome assemblies (female and male) differ considerably, which might affect their respective qualities. The final results and comparative genomics, however, show that both assemblies are very similar, indicating that both approaches are appropriate. However, there are some peculiarities in the comparison that might reflect misassemblies. Specifically, the genome alignments suggest large rearrangements within several male and female chromosomes (figure 2). Should this be interpreted as the males and females being homozygotic for these rearrangements? How would this work, biologically? Are these chromosomes still compatible? Does for example a female receive both female copies of such chromosomes from the mother fish? As an alternative explanation, are female and male fish not from the same interbreeding population?

Powered by Editorial Manager® and ProduXion Manager® from Aries Systems Corporation

Response: Thanks for your instructive questions and comments. We additionally sequenced the male individual by using PacBio Hifi and Hi-C platforms. Therefore, we obtained a new high-quality male genome assembly.

After we performed a new synteny analysis to compare the new chromosome-level male genome assembly to the original female one, we confirmed three same rearrangements as in previous results between male and female individuals. We thus believe that these rearrangements could be truthfully existed.

We also added more detailed software parameters in the revised manuscript, and all read data were uploaded and released for public availability. By the way, both male and female fishes were collected from the same interbreeding population.

For biological interpretations, we consider that this sex-differential variance could be similar to the XY or ZW sex-chromosomes in higher animals. Because arowana is an ancient teleost, the divergence between male and female specific chromosomes were not thoroughly evolutionary and taken shape. Only several preliminary inversions were identified in some chromosomes.

Ideally, I would like to see (by e.g. alignment) how these rearrangement points are supported by the different sequencing data sets.
Response: Thanks for your advice. Yes, we added detailed alignments (by the IGV software) in Supplementary Figure 8 after construction of the new male assembly with additional sequencing reads.

2. Related: It is not entirely clear if/how the HiC data processing affects the large-scale scaffolding. The HiC data are from females only, but also used for the males. As the large-scale structure is different (figure 2, supplementary figure 6), apparently this does not mean the female structure is imposed on the male data. But I am not convinced that comparing large-scale structure based on the same data is a good idea, in general.
Response: You are right. We additionally sequenced PacBio HiFi and Hi-C data of the male individual, and assembled a new chromosome-level genome assembly for the male arowana. We also compared the new male genome assembly with the female one, and identified three same rearrangements as in previous results. These new data and sex-differences are more convinced.

3. A previous publication on the Arowana genome by these authors (Bian 2016) suggests a ZW/ZZ chromosomal sex determination systems, in which females are the heterogametic sex containing a ZW pair. I see no indication of this in the new assemblies, is this correct?
Response: You are right. In fact, after obtaining high-quality chromosome-level genome assemblies of both male and female arowana, we did not confirm the heterogametic sex in the female genome. Therefore, we did not state this in our revised manuscript.

4. There are some ambiguities in the descriptions of the sequencing data. Illumina read lengths are not always mentioned, and sequencing statistics do not always agree between the text and tables:
GWAS data: supplementary table 2 has 709.2 Gbp raw, 605.7 Gbp mapped; l 263 has approximately 709.1 Gbp raw; but l 123 has 722.0 Gbp raw, 638.5 Gbp clean.
Assembly: methods do not list the 170 bp library (l 103); l 105 has 226.2 Gbp raw female, but supplementary table 1 only 73.4, and l 228 lists 98.1 Gbp clean data; l 108 (male) has 571.2 Gbp, but the table 294.4; l 113 27.4 Gbp clean PacBio are raw in supplementary table 1.
Response: Sorry for these mistakes. We carefully checked the details of sequenced reads and corrected these data throughout the revised manuscript.

5. The transcriptomics data and methods are not described in sufficient detail. For example, which normalization and statistical tests were used (using edgeR)? How many genes were tested, and how many are differentially expressed (supplementary figure 5)? Does the p-value threshold (line 195) take into account some form of multiple testing correction? What are the gene expression values in supplementary table 6 (e.g. per million reads, transcripts…)?

Response: Thanks for your advice and questions. Yes, the edgeR software was applied for normalization and statistical tests. A total of 25,328 genes were used as the input gene number, and 1,693 DEGs were identified for drawing the heatmap (Supplementary Figure 6). The p-value and fold changes were considered to identify the DEGs, although multiple testing correction has not been performed for the DEG identification. If we used the q-value, we would obtain too few DEG number. The gene expression values were calculated as FPKM (fragments per kilobase per million mapped reads). See related description in lines 197-207.

6. Since ovaries and testes are very different tissues, their expression profiles will be very different. This, in turn, invalidates scaling normalization procedures such as the edgeR default, which implicitly assume both conditions to be nearly identical apart from a few differentially expressed genes. In other words, it is not clear how to meaningfully compare apples with oranges using standard bulk RNA-seq bioinformatics.
Response: Thanks for your good questions. In fact, it is widely used for sex transcriptome comparison to map onto a male or female reference genome, because the male and female genomes of a same species are usually very similar in a large scale. Therefore, the mapping ratios of transcriptome reads and the calculation of expression values are not impacted by the different sex genomes.

However, it would be more valuable to compare these expression values of male and female individuals for identification of sex-related genes. For the comparison of apples and oranges, it couldn't be much meaningful, because the apple genome has largely different from the orange's, and these calculated expression values will be incorrect.

7. Is there any reason to expect gene expression in mature gonads to be informative of sex determination earlier in development?
Response: No. In fact, it is more serious and meaningful to detect sex-determing genes at embryo stages or sex-differentiation stages by sequencing single cell transcriptome or spatial transcriptome. We are going to fulfil this task once a local government grant is available. On the other hand, for the expectation of gene expression in mature gonads, we can discover some sex-related genes for involvement in secretion of sex hormones.

8. The GWAS involves 15+15 samples, which I think is in general low for such a study? Perhaps this is different for a qualitative trait such as sex? Is the statistical power sufficient?
Response: It is a good question. For our GWAS, because the sex is a more confirmed and significant qualitative trait comparing to body size or weight, 15+15 samples are indeed facilitated to perform the GWAS analysis and discover the significant regions. Similarly, in our previous study, we resequenced only 10 casper zebrafish, 10 roy zebrafish and 10 wild-type zebrafish to correctly identify the divergence regions for skin transparent phenotype (Bian et al., 2020).
Bian, C., Chen, W., Ruan, Z., Hu, Z., Huang, Y., Lv, Y., Xu, T., Li, J., Shi, Q., & Ge, W. (2020). Genome and Transcriptome Sequencing of casper and roy Zebrafish Mutants Provides Novel Genetic Clues for Iridophore Loss. Int J Mol Sci, 21(7):2385.

On the other hand, the Asian arowana is one of the most expensive aquarium fishes, with a market value as much as $300,000 per individual (https://biologydictionary.net/arowana/). Since its sex cannot be distinguished by morphological appearance, we killed and dissected all sampled arowana individuals to check their testis or ovary for confirmation of sexual status. By the way, we tried our best to obtain a maximum number of arowana individuals for the resequencing analysis, although we wish to obtain more samples from other countries in our next project once a local government grant is available.

9. The BUSCO methods are not described. Which version, which reference?
Response: Thanks for your advice. We added more details about the BUSCO version 5.22 and Actinopterygii odb10 dataset with related references in line 261.

10. How were the RNA-seq data filtered (l 188)?
Response: We added detailed software and parameters for the RNA-seq data filtering as follows in lines 197-199:

	Raw data were cleaned by discarding reads with adaptor sequences, >10% of non-sequenced bases, or >50% of low-quality bases by using SOAPnuke v.1.5.6 (parameters: filter -n 0.01 -l 15 -q 0.4 -G -Q 2, RRID: SCR_015025).<br><br>11. l 230: SSPACE-longer -> SSPACE-LongRead<br>Response: We revised this software name in lines 145 and 246.<br><br>12. l 128 genomes were predicted -> genome lengths were predicted<br>Response: Yes, it is corrected in line 133.<br><br>13. l 236/241: What does it mean that the final assembly covers 98.0/96.7% of the genome? Are the rest gaps from unmapped contigs?<br>Response: Yes, the rest gaps are the unmapped scaffolds (female) and contigs (male). We revised related sentences in lines 250-252 and 259 as follows:<br>The final assembly of the female individual consisted of 25 chromosomes and covered 765.8 Mb, which accounts for 98.0% of the assembled scaffolds.<br><br>Hi-C data were anchored to the draft assembly to form 25 chromosomes (ranging from 18.5 Mb to 53.0 Mb in length) and cover 747.2 Mb, which accounts for approximately 98.7% of the assembled contigs of the male individual.
**Additional Information:**	
**Question**	**Response**
Are you submitting this manuscript to a special series or article collection?	No
**Experimental design and statistics**<br><br>Full details of the experimental design and statistical methods used should be given in the Methods section, as detailed in our Minimum Standards Reporting Checklist. Information essential to interpreting the data presented should be made available in the figure legends.<br><br>Have you included all the information requested in your manuscript?	Yes
**Resources**<br><br>A description of all resources used, including antibodies, cell lines, animals and software tools, with enough information to allow them to be uniquely identified, should be included in the Methods section. Authors are strongly encouraged to cite Research Resource Identifiers (RRIDs) for antibodies, model organisms and tools, where possible.<br><br>Have you included the information	Yes

Powered by Editorial Manager® and ProduXion Manager® from Aries Systems Corporation

requested as detailed in our Minimum Standards Reporting Checklist?	
**Availability of data and materials**<br><br>All datasets and code on which the conclusions of the paper rely must be either included in your submission or deposited in publicly available repositories (where available and ethically appropriate), referencing such data using a unique identifier in the references and in the "Availability of Data and Materials" section of your manuscript.<br><br>Have you have met the above requirement as detailed in our Minimum Standards Reporting Checklist?	Yes

Powered by Editorial Manager® and ProduXion Manager® from Aries Systems Corporation

 Click here to access/download;LaTeX - Other (clo, cls, sty etc.);Review response.docx

Dear editor,

Thanks for your nice advice and the two reviewers' comments. According to your instructive opinions, we sequenced additional PacBio HiFI and Hi-C reads for the male arowana, and then performed a series of new analyses. We also made careful revisions throughout the manuscript. Our revised manuscript (changes are highlighted in yellow) and point-by-point responses are uploaded for your consideration.

Reviewer #1:

Title: Sex- specific genomic region identification in male and female Asian arowana genomes

SUMMARY

This manuscript presents a genomics analysis of a fish species, Scleropages formosus, for which there was only a draft genome previously. The authors present a high-quality female genome assembly and lower quality male genome, plus gene annotation, repeats analysis, BUSCO, transcriptomics, GWAS, and comparative genomics. These data helped identify some candidates for the sex-specific regions which remain elusive.

MAJOR ISSUE

The paper says the annotation files are available at NCBI. However, the NCBI pages say, "No public data is linked to this project." Of course, the assembly and the annotation must be released prior to publication.

Response: Thanks for your instructive comments. Yes, the genome data of both female and male individuals were released from the NCBI. They are accessible at https://www.ncbi.nlm.nih.gov/bioproject/?term=PRJNA810746 and https://www.ncbi.nlm.nih.gov/bioproject/?term=PRJNA810753.

Related annotation files were uploaded to the CNSA: https://ftp.cngb.org/pub/CNSA/data2/CNP0002889/CNS0538329/CNA0047397/.

Sequenced reads were also deposited at the CNSA for public availability: https://db.cngb.org/search/organism/113540/.

MINOR MODIFICATIONS

The experiment was well-designed and the presentation is complete. However, some aspects of the presentation should be clarified.

1. The paper must make clear that the sex-specific region was not found. The current title is ambiguous about this, and it led me to believe the region had been found. Line 296 says the discovered differences may cause sex divergence, but the paper should note that the true sex-determining region may not be among the candidates identified here.

Response: You are right. We changed the title to "Identification of candidate sex-specific genomic regions in male and female Asian arowana genomes" (lines 1-2).

2. The male assembly is a short-read assembly that was made chromosome-length using female Hi-C. Thus, long-range connectivity in the male assembly is partially derived from female fish. This is indicated in the Methods, but the analysis should note this limitation. For example, line 239 should specify, "female Hi-C". The analysis of chromosome rearrangements in Figures 2 and S6 could note that some rearrangements may be missing in the "femalized" male assembly.

Response: Thanks for your nice comments. We additionally sequenced the male individual by using PacBio HiFi and Hi-C platforms. Therefore, we obtained a new high-quality male genome assembly. We performed a solid synteny analysis to compare the new chromosome-level male genome assembly to the original female assembly again; interestingly, we identified three same rearrangements as in previous results between male and female individuals. We therefore think that these

rearrangements could be truthfully existed with more confidence. More detailed software parameters were added in the revised manuscript, and related read data were uploaded and released for public availability to make our results maximumly reproductive by other researchers.

3. The assembly pipeline needs clarification. The existing description in Methods is technically correct but potentially confusing for some readers. Add sentences or a figure describing the male and female pipelines from sequencing to assembly. Make clear the hybrid assembly process is not experimental i.e. it carefully follows processes that were previously published. For female, make clear it was polished with PacBio and polished again with Illumina. Describe the DBG2OLC hybrid step in its own sentence. State the value of K (K=17) near the formula that uses it. State how the Illumina data was "filtered."

Response: Thanks for your instructive advice and comments. Yes, we added a figure (as Supplementary Figure 3) to summarize the detailed assembling pipelines for female and male assemblies, making the assembly steps for both female and male genomes more clearly.

We added a sentence to introduce some previous studies that employed the similar hybrid assembly approach as follows in lines 147-149:

This hybrid assembly pipeline was well performed in many previous studies [20, 26, 27].

We revised the formula as follows (lines 133-135): G = N*(L–k-mer + 1)/K_depth, where k-mer length is defined as 17 bp, N is the total number of reads, and K_depth represents the frequency of occurrence more frequently than others.

For the Illumina data filtering, we revised this sentence as follows (line 129):

After filtering adapter sequences and low-quality reads by SOAPnuke v.1.5.6 with detailed parameters: filter -l 10 -q 0.1 -n 0.01 (RRID:SCR_015025) [14].

4. The Abstract says "an improved [female] reference" is presented, but the new assembly was not explicitly compared to the old ones. The text only implies (lines 80-84) that the old ones had shorter scaffolds and more gaps. It would improve the paper to include the assembly comparison. Does the new assembly contain more genes, more repeats, etc.? If such additional analysis is impossible, the abstract needs modification. The Introduction's off-hand remark that this species has been omitted from key studies due to poor quality of the previous assemblies (lines 85-86) must be justified or removed.

Response: Thanks for your nice advice. In fact, we compared the contig N50 values, gene numbers and repeat contents between this new female genome assembly and our previous one; the contig N50 of the new one was about 90 times more contiguous than the previous one. We also predicted about 3,000 more genes in this new female genome, although the repeats are similar in both assemblies. Related sentences were added in lines 250-255, 275-277 and 268-269.

5. Some unscientific language should be changed. The fish should not be subjectively called "famous" and "beautiful" in a science journal. (But perhaps a poem about the fish could be quoted?) Lines 59-60 claim that the fish is a "symbol of lucky … particularly in Asian countries"; the reference to a geography study leaves the claim unsupported so I think the whole sentence should be cut. The word "remarkable" is inappropriate to describe structural variation: lines 32 and 304. The abstract should say these assemblies "may" aid research, not "will" aid research (line 39). The statement that the assembly covers 98% of the genome (line 236) should clarify the comparison is to the genome size estimated from read coverage. The word "significant" must be changed or backed by statistics: line 290.

Response: Thanks for your instructive advice. Yes, we removed these words like “beautiful”, “symbol of lucky”, and ‘famous’ (in the abstract).

We changed ‘remarkable’ and ‘remarkably’ to ‘potential’ and ‘potentially’, and revised ‘will’ to ‘may’ in lines 34, 323, and 40 respectively.

For “98% of the genome”, we revised this sentence in lines 250-252 as follows:
The final assembly of the female individual consisted of 25 chromosomes and covered 765.8 Mb, which accounts for 98.0% of the assembled scaffolds.

We also changed the ‘significant’ to ‘potential’ in line 309.

6. Enhance the figure captions. Why does female chr 3 aligns to male chr 4? Why are alignments grouped by color in Figure S6? What is shown by the middle vertical color bar in Figure S5? What are the areas under the curves in key regions of the coverage plots in Figures S1 and S2? Perhaps insert a zoom-in histogram of the critical region so readers see each integer coverage level.
Response: Thanks for your good advice. Yes, we performed new genome-wide alignment and revised Figure 2 and Supplementary Figure 7, after we sequenced new HiFi and Hi-C data of the male and constructed a new male chromosome-level assembly.

The colors in Figure S6 were randomly selected by the RectChr software. For the Figure S5, color key indicates log2-transformed transcription values; we added this description in the Supplementary Figure 6 (original Figure S5).

We supplemented the peak coverage of the kmer curves in captions of the Figures S1 and S2. For the integer coverage level of each inversion boundary (also known as breakpoint), we first applied the minimap2 with default parameters to align PacBio reads to the male genome assembly, and then used the Integrative Genomics Viewer software to show the detailed read coverage of the critical regions in Supplementary Figure 8.

RECOMMENDATIONS
This manuscript aligns well with the journal's instruction for authors that says, "One of the aims of a Data Note is to incentivize and more rapidly release data before subsequent detailed analysis has been carried out." However, the authors may want to improve this paper by analyzing some additional aspects of their data.
1. Address the male/female assembly quality difference. The paper says "both genome assemblies are indeed of high quality and completeness" based on BUSCO: line 246. However, the paper has indications of a difference in quality: the male had lower resequencing map rate (line 264) and lower repeat content (line 250). Provide the male contig N50, not just scaffold N50 (line 239). How would results have changed if you assembled the female from Illumina-only, as was done for male? What was gained from the additional resources invested in the female assembly?
Response: Thanks for your good questions and comments. In fact, we additionally sequenced PacBio HiFi and Hi-C data of the male individual, and obtained a new chromosome-level genome assembly for the male arowana. In this way, both male and female genomes are of high quality. We also compared the new male genome assembly with the female assembly, which confirms three same rearrangements as in previous results.

2. When female Hi-C was applied to male contigs, were any contig breaks suggested?

Response: We additionally sequenced Hi-C data of the male individual, and assembled a new chromosome-level genome assembly for the male arowana.

3. Of the intersex chromosome rearrangements found, what genes are nearby?
Response: We revised the Figure 2 and added detailed gene ids and functional descriptions near the inversion boundaries.

Reviewer #2:
This paper describes two new genome assemblies, as well as two experimental datasets, all aimed at understanding sex determination in the Asian arowana. In general, the data and methods used are well suited for addressing this issue.
There are a few issues I would like to ask the authors to comment on:
1. The methods for both genome assemblies (female and male) differ considerably, which might affect their respective qualities. The final results and comparative genomics, however, show that both assemblies are very similar, indicating that both approaches are appropriate. However, there are some peculiarities in the comparison that might reflect misassemblies. Specifically, the genome alignments suggest large rearrangements within several male and female chromosomes (figure 2). Should this be interpreted as the males and females being homozygotic for these rearrangements? How would this work, biologically? Are these chromosomes still compatible? Does for example a female receive both female copies of such chromosomes from the mother fish? As an alternative explanation, are female and male fish not from the same interbreeding population?
Response: Thanks for your instructive questions and comments. We additionally sequenced the male individual by using PacBio Hifi and Hi-C platforms. Therefore, we obtained a new high-quality male genome assembly.

After we performed a new synteny analysis to compare the new chromosome-level male genome assembly to the original female one, we confirmed three same rearrangements as in previous results between male and female individuals. We thus believe that these rearrangements could be truthfully existed.

We also added more detailed software parameters in the revised manuscript, and all read data were uploaded and released for public availability. By the way, both male and female fishes were collected from the same interbreeding population.

For biological interpretations, we consider that this sex-differential variance could be similar to the XY or ZW sex-chromosomes in higher animals. Because arowana is an ancient teleost, the divergence between male and female specific chromosomes were not thoroughly evolutionary and taken shape. Only several preliminary inversions were identified in some chromosomes.

Ideally, I would like to see (by e.g. alignment) how these rearrangement points are supported by the different sequencing data sets.
Response: Thanks for your advice. Yes, we added detailed alignments (by the IGV software) in Supplementary Figure 8 after construction of the new male assembly with additional sequencing reads.

2. Related: It is not entirely clear if/how the HiC data processing affects the large-scale scaffolding. The HiC data are from females only, but also used for the males. As the large-scale structure is different (figure 2, supplementary figure 6), apparently this does not mean the female structure is imposed on the male data. But I am not convinced that comparing large-scale structure based on the same data is a good idea, in general.

Response: You are right. We additionally sequenced PacBio HiFi and Hi-C data of the male individual, and assembled a new chromosome-level genome assembly for the male arowana. We also compared the new male genome assembly with the female one, and identified three same rearrangements as in previous results. These new data and sex-differences are more convinced.

3. A previous publication on the Arowana genome by these authors (Bian 2016) suggests a ZW/ZZ chromosomal sex determination systems, in which females are the heterogametic sex containing a ZW pair. I see no indication of this in the new assemblies, is this correct?
Response: You are right. In fact, after obtaining high-quality chromosome-level genome assemblies of both male and female arowana, we did not confirm the heterogametic sex in the female genome. Therefore, we did not state this in our revised manuscript.

4. There are some ambiguities in the descriptions of the sequencing data. Illumina read lengths are not always mentioned, and sequencing statistics do not always agree between the text and tables:
GWAS data: supplementary table 2 has 709.2 Gbp raw, 605.7 Gbp mapped; l 263 has approximately 709.1 Gbp raw; but l 123 has 722.0 Gbp raw, 638.5 Gbp clean.
Assembly: methods do not list the 170 bp library (l 103); l 105 has 226.2 Gbp raw female, but supplementary table 1 only 73.4, and l 228 lists 98.1 Gbp clean data; l 108 (male) has 571.2 Gbp, but the table 294.4; l 113 27.4 Gbp clean PacBio are raw in supplementary table 1.
Response: Sorry for these mistakes. We carefully checked the details of sequenced reads and corrected these data throughout the revised manuscript.

5. The transcriptomics data and methods are not described in sufficient detail. For example, which normalization and statistical tests were used (using edgeR)? How many genes were tested, and how many are differentially expressed (supplementary figure 5)? Does the p-value threshold (line 195) take into account some form of multiple testing correction? What are the gene expression values in supplementary table 6 (e.g. per million reads, transcripts…)?
Response: Thanks for your advice and questions. Yes, the edgeR software was applied for normalization and statistical tests. A total of 25,328 genes were used as the input gene number, and 1,693 DEGs were identified for drawing the heatmap (Supplementary Figure 6). The *p*-value and fold changes were considered to identify the DEGs, although multiple testing correction has not been performed for the DEG identification. If we used the q-value, we would obtain too few DEG number. The gene expression values were calculated as FPKM (fragments per kilobase per million mapped reads). See related description in lines 197-207.

6. Since ovaries and testes are very different tissues, their expression profiles will be very different. This, in turn, invalidates scaling normalization procedures such as the edgeR default, which implicitly assume both conditions to be nearly identical apart from a few differentially expressed genes. In other words, it is not clear how to meaningfully compare apples with oranges using standard bulk RNA-seq bioinformatics.
Response: Thanks for your good questions. In fact, it is widely used for sex transcriptome comparison to map onto a male or female reference genome, because the male and female genomes of a same species are usually very similar in a large scale. Therefore, the mapping ratios of transcriptome reads and the calculation of expression values are not impacted by the different sex genomes.

However, it would be more valuable to compare these expression values of male and female individuals for identification of sex-related genes. For the comparison of

apples and oranges, it couldn't be much meaningful, because the apple genome has largely different from the orange's, and these calculated expression values will be incorrect.

7. Is there any reason to expect gene expression in mature gonads to be informative of sex determination earlier in development?

Response: No. In fact, it is more serious and meaningful to detect sex-determing genes at embryo stages or sex-differentiation stages by sequencing single cell transcriptome or spatial transcriptome. We are going to fulfil this task once a local government grant is available. On the other hand, for the expectation of gene expression in mature gonads, we can discover some sex-related genes for involvement in secretion of sex hormones.

8. The GWAS involves 15+15 samples, which I think is in general low for such a study? Perhaps this is different for a qualitative trait such as sex? Is the statistical power sufficient?

Response: It is a good question. For our GWAS, because the sex is a more confirmed and significant qualitative trait comparing to body size or weight, 15+15 samples are indeed facilitated to perform the GWAS analysis and discover the significant regions. Similarly, in our previous study, we resequenced only 10 *casper* zebrafish, 10 *roy* zebrafish and 10 wild-type zebrafish to correctly identify the divergence regions for skin transparent phenotype (Bian et al., 2020).

Bian, C., Chen, W., Ruan, Z., Hu, Z., Huang, Y., Lv, Y., Xu, T., Li, J., Shi, Q., & Ge, W. (2020). Genome and Transcriptome Sequencing of casper and roy Zebrafish Mutants Provides Novel Genetic Clues for Iridophore Loss. *Int J Mol Sci*, *21*(7):2385.

On the other hand, the Asian arowana is one of the most expensive aquarium fishes, with a market value as much as $300,000 per individual (https://biologydictionary.net/arowana/). Since its sex cannot be distinguished by morphological appearance, we killed and dissected all sampled arowana individuals to check their testis or ovary for confirmation of sexual status. By the way, we tried our best to obtain a maximum number of arowana individuals for the resequencing analysis, although we wish to obtain more samples from other countries in our next project once a local government grant is available.

9. The BUSCO methods are not described. Which version, which reference?

Response: Thanks for your advice. We added more details about the BUSCO version 5.22 and Actinopterygii odb10 dataset with related references in line 261.

10. How were the RNA-seq data filtered (l 188)?

Response: We added detailed software and parameters for the RNA-seq data filtering as follows in lines 197-199:

Raw data were cleaned by discarding reads with adaptor sequences, >10% of non-sequenced bases, or >50% of low-quality bases by using SOAPnuke v.1.5.6 (parameters: filter -n 0.01 -l 15 -q 0.4 -G -Q 2, RRID: SCR_015025).

11. l 230: SSPACE-longer -> SSPACE-LongRead

Response: We revised this software name in lines 145 and 246.

12. l 128 genomes were predicted -> genome lengths were predicted

Response: Yes, it is corrected in line 133.

13. l 236/241: What does it mean that the final assembly covers 98.0/96.7% of the genome? Are the rest gaps from unmapped contigs?

Response: Yes, the rest gaps are the unmapped scaffolds (female) and contigs (male). We revised related sentences in lines 250-252 and 259 as follows:

The final assembly of the female individual consisted of 25 chromosomes and covered 765.8 Mb, which accounts for 98.0% of the assembled scaffolds.

Hi-C data were anchored to the draft assembly to form 25 chromosomes (ranging from 18.5 Mb to 53.0 Mb in length) and cover 747.2 Mb, which accounts for approximately 98.7% of the assembled contigs of the male individual.

Manuscript

Click here to access/download;Manuscript;manuscript 20220626.docx

Identification of candidate sex-specific genomic regions in male and female Asian arowana genomes

Xidong Mu[1†]*, Yi Liu [1†], Chao Liu[1], Chenxi Zhao[2, 3], Ruihan Li[2, 3], Xinxin You[2, 3], Yexin Yang[1,4], Xuejie Wang[1], Yinchang Hu[1], Qiong Shi[2, 3], Chao Bian[2, 3]*

[1.]Key Laboratory of Prevention and Control for Aquatic Invasive Alien Species, Ministry of Agriculture and Rural Affairs, Guangdong Modern Recreational Fisheries Engineering Technology Center, Pearl River Fisheries Research Institute, Chinese Academy of Fishery Sciences, Guangzhou 510380, China

[2]Shenzhen Key Lab of Marine Genomics, Guangdong Provincial Key Lab of Molecular Breeding in Marine Economic Animals, BGI Academy of Marine Sciences, BGI Marine, BGI, Shenzhen 518083, China

[3]College of Life Sciences, University of Chinese Academy of Sciences, Beijing 100049, China

[4]Key Laboratory of Aquatic Animal Immune Technology of Guangdong Province, Guangzhou 510380, China

† These authors contributed equally to this study

*Correspondence and requests for materials should be addressed to Xidong Mu (email: muxd@prfri.ac.cn) and Chao Bian (email: bianchao@genomics.cn)

Abstract

Background: Asian arowana, *Scleropages formosus*, is one of the most expensive aquarium fish species worldwide. Its sex, however, cannot be distinguished clearly at any development stage, which impedes captive breeding and species protection for this endangered aquarium fish.

Results: To discover molecular clues to the sex of Asian arowana, we sequenced 26.5 Gb of PacBio HiFi reads and 179.2 Gb of Hi-C reads for one male fish, and also sequenced 106.5 Gb of Illumina reads, 36.0 Gb of PacBio Sequel reads and 80.7 Gb

of Hi-C reads for one female individual. The final male and female genome assemblies were approximately 756.8 Mb and 781.5 Mb in length and contained 25,262 and 25,328 protein-coding genes respectively. We also resequenced the genomes of 15 male and 15 female individuals with approximately 722.1 Gb of Illumina reads. A genome-wide association study (GWAS) identified several potentially divergent regions between male and female individuals. In these regions, *cd48* and *cfap52* could be candidate genes for sex determination of Asian arowana. We also found some structural variations in few chromosomes between male and female individuals.

Conclusion: We provided an improved reference genome assembly of female arowana and generated the first sequenced genome of one male individual. These valuable genetic resources and resequencing data may improve global aquarium fish research.

Key words: Asian arowana; male and female; genome sequencing and resequencing; sex-related genes

Introduction

Scleropages formosus, also known as Asian arowana, belongs to the genus *Scleropages* of family Osteoglossidae, order Osteoglossiformes. This monophyletic fish order represents an ancient teleost group with a geographic distribution restricted to freshwater river basins. *Scleropages* are a primary group of ancient origin, and their distribution is tied to land/continental evolution [1]. Asian arowana include three major varieties (the golden, red and green varieties) in nature. They are widely distributed throughout Southeast Asia, including Cambodia, Indonesia, Laos, the Malay Archipelago, the Philippines, Vietnam and Thailand [2]. The Asian arowana are also named bonytongue due to their primitive characteristic of large tooth plates on their tongues [3]. A previous study showed that the Sundaland–Indochina species were the sister group of the two Australian species within *Scleropages*, and the

estimated divergence time of crown-group *Scleropages* ranged from 79.9 Ma to 101.4 Ma [4].

Asian arowana skin is covered by large and bright conspicuous color scales. Because of the high demand for this species and its high price, overfishing has led to the drastic population decline of Asian arowana. It has been listed as an endangered species by the Convention on International Trade in Endangered Species of Wild Fauna and Flora (CITES) Appendix I [5].

On the other hand, the sex of Asian arowana is not distinguishable morphologically at any stage of development, even after sexual maturity. Additionally, the mechanism of sex determination is also largely unknown [6]. The lack of a genetic sex identification method critically hinders its further development of captive breeding for aquaculture and species protection for this endangered fish. In previous reports, genetic and genomic methods have been used for sex identification. Sequence-tagged site (STS) markers have been identified; however, these markers can only be applied in certain stains, and the accuracy of detection is not high [7]. Shen et al. (2014) identified and mapped potentially sex-related genes (such as *dmrt2*, *dmrt4* and *sox9*) by transcriptome data and linkage map, while no mutations were found within these sex-related candidate genes [8].

Regarding the increasing popularity of high-throughput sequencing methodologies, it may be possible to identify sex-determining genes using linkage mapping or genome-wide association study (GWAS) [9]. The complete genome of Asian arowana was first sequenced in 2015, which was a draft assembly with an N50 scaffold length of 59.0 kb [10]. A chromosome-level genome of a female golden-variety arowana was reported by using a combination of deep shotgun sequencing and high-resolution linkage mapping [11]. In addition, two draft genome assemblies for the red and green varieties were also generated. The N50 scaffold sizes of the three varieties genomes were 6.0, 1.6 and 1.9 Mb, respectively, but the N50 contig sizes are very short (30.7, 60.2 and 62.8 kb, respectively). Given that there are still many gaps

in the draft genomes of Asian arowana, their inclusion in studies to investigate some biological issues has still been limited. To enhance assembly quality, the wide use of long genomic reads (<100 kb in length) produced by third-generation sequencing technologies can cover long repeat regions and substantially reduce fragmentation [12]. Third-generation sequencing technology can also refine the published draft assemblies to a nearly complete genome by spanning gaps for further genomic analyses [13].

In this study, we combined PacBio third-generation sequencing technology with Illumina second-generation sequencing and Hi-C technologies to assemble male and female genomes of Asian arowana. Transcriptome sequencing and whole genome resequencing were also performed from both male and female individuals with a particular attention to sex-specific differences.

Methods

Sample collection and sequencing

We extracted genomic DNAs from muscle tissues of one female and one male golden arowana and sequenced them by using an Illumina HiSeq Xten sequencing platform (San Diego, CA, USA). The construction of DNA libraries (short-insert sizes of 170, 500 and 800 bp, and large-insert sizes of 2, 5, 10 and 20 kb) and subsequent sequencing were performed according to the manufacturer's standard protocols. In total, approximately 106.5 Gb of female raw data was generated (Supplementary Table 1). After filtering adapter sequences and low-quality reads by SOAPnuke v.1.5.6 with detailed parameters: filter -l 10 -q 0.1 -n 0.01 (RRID:SCR_015025) [14], we obtained 73.4 Gb of Illumina clean reads. We also sequenced the female individual on a PacBio Sequel sequencing platform (Menlo Park, CA, USA). A 20-kb library was constructed, and then 4 SMRT cells were produced using P6 polymerase/C4 chemistry, generating 36.0 Gb of PacBio long reads. After correcting and trimming the PacBio raw reads by using LoRDEC (RRID:SCR_015814) [15]

with Illumina short reads, 27.4 Gb of clean PacBio reads were obtained (Supplementary Table 1). To acquire a chromosome-level genome assembly, genomic DNAs from the female muscle tissue was fixed with formaldehyde, sheared by a restriction enzyme (MboI) to build a Hi-C library, and then sequenced by an Illumina HiSeq Xten sequencing platform. A total of 80.7 Gb of 150-bp paired-end Hi-C data were generated (Supplementary Table 1).

The male sample was also collected for construction of a chromosome-level genome assembly. Genome DNAs from the muscle tissues were sequenced on a Pacbio HiFi platform. PacBio recently updated its platforms to generate HiFi reads using the circular consensus sequencing mode with a base-level resolution of over 99% [16, 17]. In total, 26.5 Gb of HiFi reads and 179.2 Gb of Hi-C reads (Supplementary Table 1) were obtained after sequencing.

Genomic DNAs from muscle tissues of 30 golden arowana (15 female and 15 male) were extracted, and 350-bp insert libraries were constructed. A total of 30 libraries were sequenced on an Illumina HiSeq Xten sequencing platform. A total of 722.1 Gb of raw reads were generated, 654.9 Gb of clean reads were obtained through SOAPnuke v.1.5.6 (parameters: filter -l 10 -q 0.1 -n 0.01, RRID: SCR_015025) filtering, and 617.6 Gb of data were mapped (Supplementary Table 2).

Genome assembly and chromosome linkage

The male and female genome lengths were predicted by a k-mer analysis [18] according to the following formula: G = N*(L–k-mer + 1)/K_depth, where k-mer length is defined as 17 bp, N is the total number of reads, and K_depth represents the frequency of occurrence more frequently than others.

A hybrid genome assembly pipeline was employed to obtain a female genome assembly. Short Illumina reads were first assembled by using Platanus version 1.2.1 (RRID:SCR_015531) [19]. DBG2OLC [20] was performed to combine Platanus-generated contigs with PacBio long reads to generate a hybrid contig assembly with

default parameters. The error-corrected and consensus assembly was generated by minimap2 v2.17 (RRID:SCR_018550) [21] and Racon v1.2.1 (RRID:SCR_017642) [22] using the raw PacBio data. Pilon v1.225 (RRID:SCR_014731) [23] was subsequently employed to polish the hybrid assembly with Illumina short reads. SSPACE-LongRead version 1.1 (RRID:SCR_005056) [24] was applied to construct scaffolds based on PacBio data, and Illumina data were used to join scaffolds through SSPACE version 3.0 (RRID:SCR_005056) [25]. The detailed female assembly pipeline was summarized in Supplementary Figure 3. This hybrid assembly pipeline was well performed in many previous studies [20, 26, 27]. We performed quality control of Hi-C raw reads and obtained valid Hi-C connected reads by Juicer version 1.5 (RRID:SCR_017226) [28]. The 3D *de novo* assembly (3D-DNA, version 180922) pipeline [29] was applied to anchor primary scaffolds into chromosome-level scaffolds (Supplementary Figures 4).

The male genome assembly was constructed with WTDBG2 (RRID:SCR_017225; parameters: -x ccs -g 789m -t 32; RRID:SCR_017225) [30]. We also used the Hi-C data of male individual to join the male assembly into chromosomes through the Juicer-3D-DNA pipeline (Supplementary Figure 5). The detailed male assembly pipeline was provided in the Supplementary Figure 3.

Gene prediction and annotation

Repetitive elements in the female and male assemblies were predicted through a combination of homolog-based and *de novo* approaches. For the homolog-based method, RepeatMasker v4.0.7 (RRID:SCR 012954) [31] and RepeatProteinMask v.4.0.7 [31] were used to detect repeats by alignment against the Repbase database v21.0 [32]. For the *de novo* method, LTR-Finder v.1.0.7 [33] was applied to predict full long terminal repeat (LTR) retrotransposons. RepeatModeler v1.0.11 [31] was employed to build transposable element (TE) consensus sequences as a *de novo* TE library, and TRF v.4.09 [34] was used to obtain tandem repetitive sequences.

RepeatMasker was then used to discover and identify repetitive sequences with the combined library of the *de novo* TEs.

Protein-coding genes were annotated by the BRAKER2 v2.1.5 pipeline (RRID:SCR_018964) [35] with repeat-masked male and female genomes. We masked the repetitive sequence of both genome assemblies, and HISAT2 v0.1.6 (RRID:SCR_015530) was employed to align the transcriptome data to the assembled genomes. Protein sequences of *Danio rerio*, *Gasterosteus aculeatus*, *Takifugu rubripes* and *Tetraodon nigroviridis* downloaded from Ensembl-release99 and the Asian arowana gene protein sequences [36] were used as homology-based evidence. Finally, BRAKER2 was used to annotate the genomes with Augustus version 3.3.3 (RRID:SCR 008417) and GeneMark-ET (v4.46, topaz.gatech.edu/license download.cgi).

Gene functional annotation was performed based on the consensus of sequence and domain. The protein sequences were aligned to the NCBI Non-Redundant Protein Sequence (NR) databases, Kyoto Encyclopedia of Genes and Genomes (KEGG) [37], SwissProt and TrEMBL (UniProt release 2020-06) [38] with BLASTp. The domains were searched and predicted by using InterProScan version 5.11 (RRID: SCR_005829) [39, 40] with publicly available databases, including PANTHER [41], Pfam [42], PRINTS [43], ProDom [44], PROSITE profiles [45], and SMART [46]. Gene ontology (GO) terms [47] for each gene were predicted from the InterPro descriptions.

Transcriptome analysis of ovary and testis tissues

For transcriptome sequencing, total RNAs were collected from three ovary tissues of three female individuals and three testis tissues of three male individuals by using TRIzol reagent (Invitrogen, Carlsbad, CA, USA). The reverse transcription step was then performed on these extracted RNAs. Paired-end reads (150 bp) were produced by a HiSeq XTEN platform. Raw data were cleaned by discarding reads with adaptor

sequences, >10% of non-sequenced bases, or >50% of low-quality bases by using SOAPnuke v.1.5.6 (parameters: filter -n 0.01 -l 15 -q 0.4 -G -Q 2, RRID: SCR_015025). These filtered RNA reads were mapped onto the female genome assembly by using HISAT2 v0.1.6 (RRID:SCR_015530) with the parameters "--phred33 --sensitive --no-discordant --no-mixed -I 1 -X 1000" [48]. Cufflinks v2.2.1 (RRID:SCR_014597) with default parameters [49] was used to calculate expression values as fragments per kilobase per million mapped reads (FPKM) from three ovary samples and three testis samples. EdgeR software (RRID:SCR_012802) [50] was employed to identify the differentially expressed genes (DEGs, p <0.05 and folds >2). A total of 25,328 genes were used as the input, and 1,693 DEGs were identified for drawing a heatmap (Supplementary Figure 6).

Resequencing analysis

Quality-controlled reads from 30 samples were then aligned to the female assembly by using Burrows Wheeler Aligner v0.7.17 (BWA; RRID:SCR_010910) with default parameters [51]. The depth of each base was stated by Samtools v1.7 (RRID:SCR_002105). The BaseRecalibrator and ApplyBQSR module of Genome Analysis Tool Kit v4.1.2.0 (GATK; RRID:SCR_001876) [52] was used to correct the base quality. The HaplotypeCaller module was used for variant calling, and the concordant variants were filtered with "QD < 2.0 || MQ < 40.0 || ReadPosRankSum < -8.0 || FS > 60.0|| MQRankSum < -12.5".

For genome-wide association studies (GWAS), EMMAX [53] with the MLM and case control generated by PLINK v1.07 (RRID:SCR_001757)[54] were employed to detect associations based on male and female populations. The score assignment of phenotypic traits of each group in the GWAS analysis included 1 for female individuals and 2 for male individuals. Significance levels of genotype-phenotype association (p) were calculated by using Fisher's exact test under a recessive model.

The kinship of each population was measured by Tassel with default parameters, and the R package 'qqman' [55] was applied to make Manhattan plots.

Analysis of chromosome structural variations

A synteny analysis between the genomes of male and female arowana was performed by MUMmer software v4.0beta1 (RRID:SCR_018171) [56]. The alignment of the two genomes was completed by the Nucmer module. The alignment identity (>0.9) and alignment length (<2 kb) were retained. Finally, the chromosome synteny regions and structural variations were visualized using RectChr software (https://github.com/BGI-shenzhen/RectChr). To confirm the structure variations, we used the Minimap2 (RRID:SCR_018550)[57] with default parameters to align PacBio HiFi reads to the male genome, and then employed the Integrative Genomics Viewer software (RRID:SCR_011793)[58] to examine alignments and to show the detailed read coverage of the critical regions in Supplementary Figure 8.

Results

Genome sequencing and assembly

We sequenced the genome of a female by using an Illumina HiSeq sequencing platform as well as a PacBio Sequel sequencing platform. After data filtering, we obtained a total of 73.4 Gb of clean Illumina short reads and 27.4 Gb of clean PacBio Sequel long reads (Supplementary Table 2). Employing the hybrid assembly method, we obtained a draft genome of 780.9 Mb with a contig N50 of 2.7 Mb. After scaffolding by SSPACE-LongRead and SSPACE, we generated a genome of 781.1 Mb with a scaffold N50 of 4.2 Mb. A total of 80.7 Gb of Hi-C data were analyzed by Juicer, and contigs in the draft assembly were subsequently anchored into chromosomes by a 3D-DNA pipeline, resulting in a polished genome assembly of 781.5 Mb, with an improved scaffold N50 of 29.8 Mb (Table 1). The final assembly of the female individual consisted of 25 chromosomes and covered 765.8 Mb, which

accounts for 98.0% of the assembled scaffolds. The length of each chromosome ranged from 18.7 Mb to 55.9 Mb. This female genome was about 90 times more contiguous with a contig N50 of 2.7 Mb relative to a contig N50 of 0.03 Mb of the previous assembly [11].

For male individuals, WTDBG2 was used to generate a 756.8 Mb assembly with a contig N50 of 7.8 Mb. Hi-C data were anchored to the draft assembly to form 25 chromosomes (ranging from 18.5 Mb to 53.0 Mb in length) and cover 747.2 Mb, which accounts for approximately 98.7% of the assembled contigs of the male individual.

We also confirmed that approximately 96.5% (93.8% single-copy and 2.7% duplicated) and 96.1% of complete reference genes (93.7% single-copy and 2.4% duplicated) of BUSCO results (version 5.22 and Actinopterygii odb10 reference) [59] were detectable in the final female and male genome assemblies. These results confirm that both assemblies are indeed of high quality and completeness.

Gene prediction and annotation

In total, approximately 27.8% of the female assembly sequences (similar to the 27.3% of previous female arowana assembly) [11] and 33.4% of the male assembly sequences were annotated as repetitive elements. The repetitive sequences include 129.2 Mb (~16.5%) of long interspersed elements (LINEs) in the female individual and 134.4 Mb (~17.8%) in the male individual (Supplementary Tables 3 and 4).

Using the repeat-masked genome assemblies, we predicted a total of 25,328 genes from the female individual and 25,262 from the male individual (Table 2). Compared with the gene number (22,016) of the previous female arowana assembly, we predicted about 3,000 more genes in this female assembly with longer continuous contigs [11]. Based on functional annotation, we predicted 22,250 protein-coding genes (~87.9%) from the female individual and 21,343 (~84.6%) protein-coding

genes from the male individual with at least one assignment from the Swiss-Prot, TrEMBL, Nr, KEGG or InterPro databases.

Male and female resequencing data

Genome resequencing of 15 males and 15 females generated approximately 722.1 Gb of raw data. The mapping ratio for each sample ranged from 81.4% to 87.9%, and the mean mapped depth was approximately 30-fold. A total of 8.9 million (M) high-confidence SNPs were identified, and they were then annotated based on their positions in the chromosomes. Most of the SNPs (5.4 M, 60.7%) were localized in intergenic regions. Approximately 3.2 M of the SNPs (36.0%) fell in intron regions, and only 0.3 M of the SNPs (3.4%) distributed in coding regions. Among these SNPs within coding regions, 142,646 synonymous SNPs and 122,373 nonsynonymous SNPs were identified (Supplementary Table S5).

Candidate sex-related loci and DEGs between male and female individuals

A GWAS study among the sequenced 15 male and 15 female individuals revealed the most significant peak in Chr14. The detailed significant region (P value= 3.3e-12) in Chr14 ranged from 982,221 bp to 1,276,785 bp. This contains a *cd48* gene encoding CD48 antigen (Supplementary Table 6). On the other hand, after combining the transcriptome data, we found that a *cfap52* gene (encoding cilia- and flagella-associated protein 52 isoform X1) located in a potential sex divergence region of Chr19 predicted by the GWAS method was more highly expressed in testis than in ovary. It is worth noting that *cfap52* deficiency can result in situ inversus totalis and even lead to male infertility [60]. Therefore, we suggest that the *cd48* gene from GWAS results and the *cfap52* from both GWAS and transcriptome results could be candidate sex-related genes in Asian arowana.

Structural variations between male and female individuals

The female genome was aligned onto the male genome to identify sex differences in chromosome structures (Figure 2). Aligned regions were over 90% of the total chromosome length of both individuals (Supplementary Table 7). Three potential chromosomal inversions were detected after all-against-all alignments (Figure 2). Two chromosome inversions occurred on the terminal regions of Chr6 and Chr10 of the female individual, corresponding to Chr6M (0-3.1 Mb) and Chr10M (29.1-30.5 Mb) of the male individual, respectively (Figure 2A). Moreover, an inversion occurs in the interior regions of Chr21 of the female, corresponding to Chr21M (1.6-2.2 Mb) of the male (Figure 2B). These differences in chromosome structure may cause sex divergence between male and female individuals.

Conclusions

In summary, we generated a high-quality and high-completeness genome assembly of female arowana and sequenced the genome of one male individual. GWAS and transcriptome analyses have identified two candidate genes that may play sex-determining roles in male and female individuals. Chromosome alignments also showed some potential structural variations between male and female individuals. These valuable genetic resources including genome and transcriptome data will facilitate the molecular breeding of this economically important fish species.

Data Availability

The genome sequences of male and female arowana individuals are available in NCBI under accession nos. PRJNA810753 and PRJNA810746. The genome annotation and protein files of male and female individuals are available in the CNGB database: https://ftp.cngb.org/pub/CNSA/data2/CNP0002832/CNS0567336/CNA0050694/ and https://ftp.cngb.org/pub/CNSA/data2/CNP0002889/CNS0538329/CNA0047397/. The genome reads, transcriptome reads, and resequencing reads are deposited in the CNSA database: https://db.cngb.org/search/organism/113540/.

Additional Files

Supplementary Figure 1. 17-kmer analysis for prediction of genome size of the female individual.

Supplementary Figure 2. 17-kmer analysis for prediction of genome size of the male individual.

Supplementary Figure 3. Detailed assembling pipelines of female and male.

Supplementary Figure 4. Heatmap of the Hi-C result of female individual.

Supplementary Figure 5. Heatmap of the Hi-C result of male individual.

Supplementary Figure 6. A heatmap of DEGs in the testis and ovary tissues of three male and three female individuals.

Supplementary Figure 7. Chromosomal alignments of male and female chromosomes.

Supplementary Figure 8. Integrated Genome Visualisation screenshot of inversion boundaries of Chr6M, Chr10M and Chr21M chromosomes.

Supplementary Table 1. Summary of sequenced reads for male and female genomes.

Supplementary Table 2. Summary of map ratio for the 30 male and female samples.

Supplementary Table 3. Repetitive elements in the assembled genome of a female individual.

Supplementary Table 4. Repetitive elements in the assembled genome of a male individual.

Supplementary Table 5. Chromosome location of SNPs.

Supplementary Table 6. Genes in potential sex divergence regions in chromosomes predicted by the GWAS and their expression values in ovary and testis tissues.

Supplementary Table 7. Statistics of the mapped ratio of chromosomes of male and female individuals.

Abbreviations

BUSCO: Benchmarking Universal Single-Copy Orthologs; GATK: Genome Analysis Toolkit; Gb: gigabase; Mb: megabase; ML: maximum likelihood; NCBI: National Center for Biotechnology Information; SNP: single-nucleotide polymorphism.

Competing Interests

The authors declare that they have no competing interests.

Funding

This study was supported by the Central Public-interest Scientific Institution Basal Research Fund, CAFS (No. 2016HY-ZCO402, No. 2019ZD0503, No. 2020TD17), the Guangdong Provincial Special Fund for Modern Agriculture Industry Technology Innovation Team (2022KJ150), China-ASEAN Maritime Cooperation Fund (No. CAMC-2018F), Guangzhou Scientific Planning Program (No. 201904010409), and National Freshwater Genetic Resource Center (FGRC18537).

Authors’ Contributions

X Mu and C Bian designed the research; Y Liu, C Liu and Y Yang collected samples and conducted experiments; C Liu, X Wang and Y Hu performed artificial breeding; C Bian, C Zhao, R Li, X You, Q Shi and X Mu analyzed the data; C Bian, Y Liu and X Mu wrote the manuscript; C Bian, Q Shi and X Mu revised the manuscript.

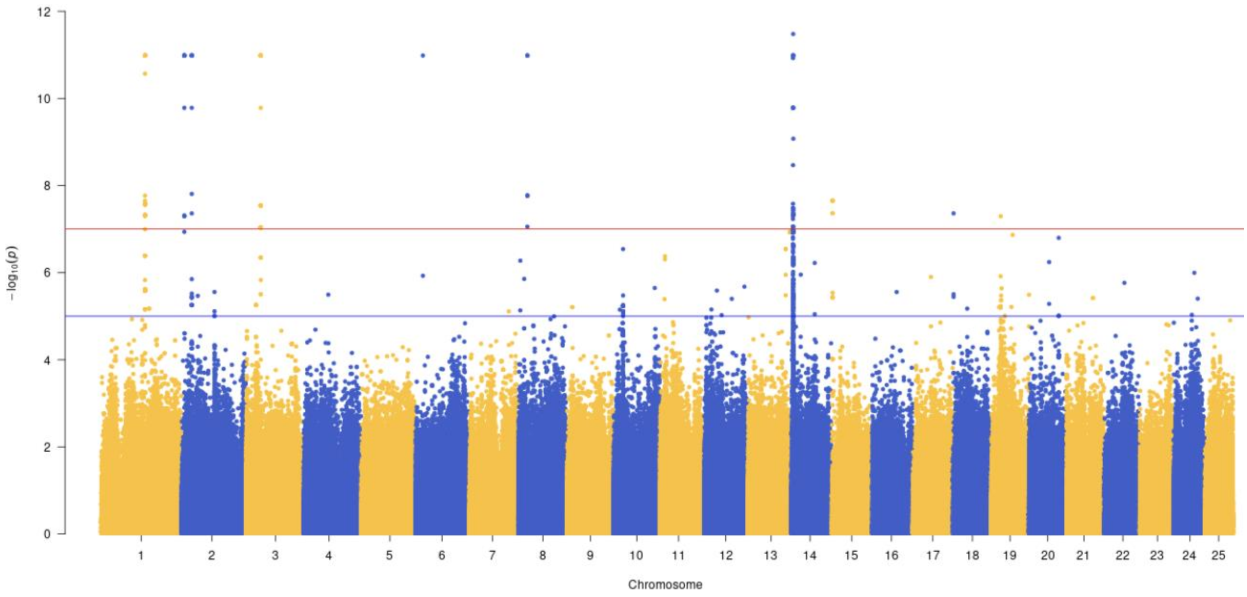

Figure 1. Manhattan figure demonstrating GWAS results between male and female individuals. The x- and y-axes represent SNP localizations in chromosomes and corresponding *p* values, respectively.

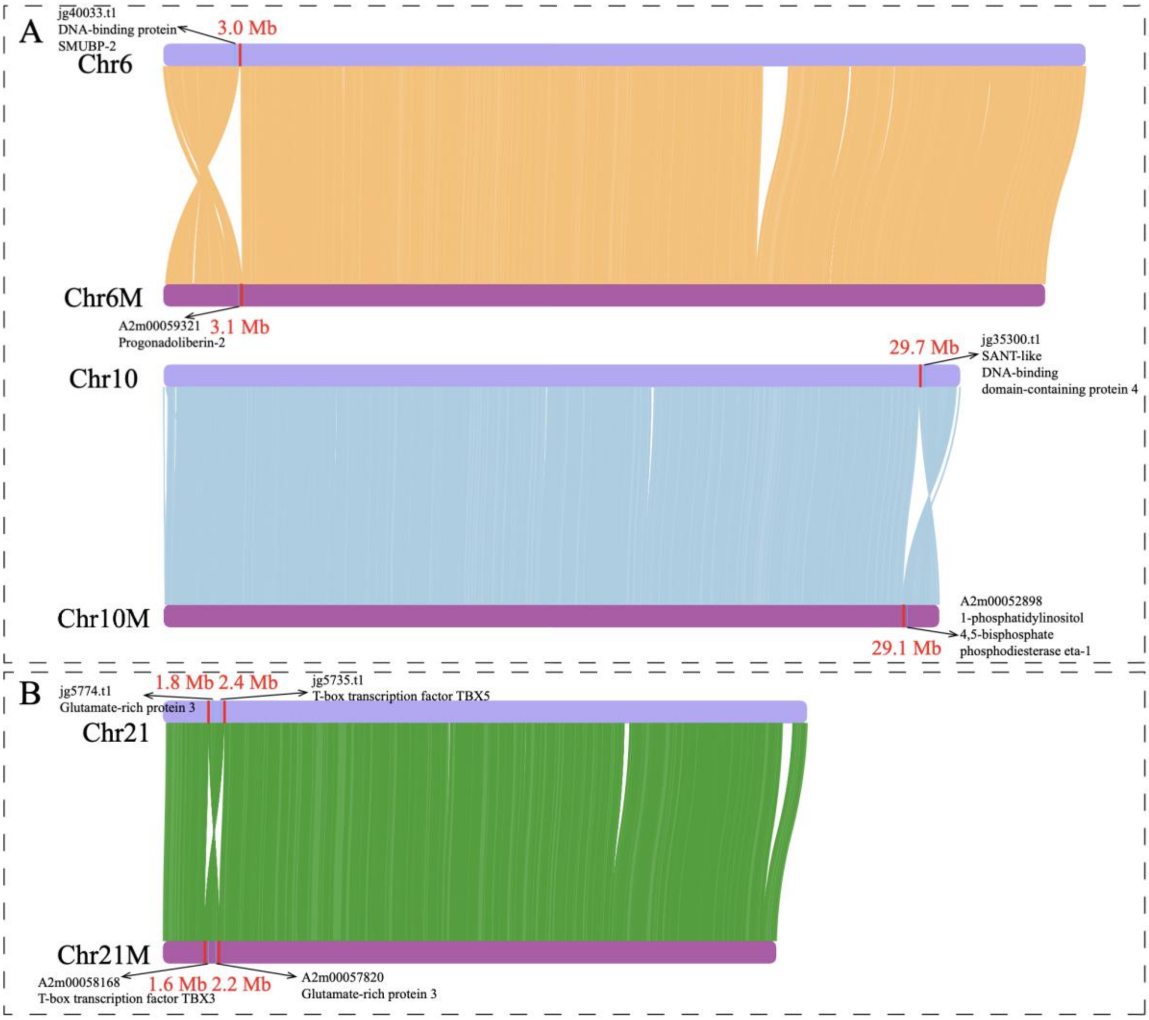

Figure 2. Chromosomal inversion events between male and female individuals. Chr6, Chr10, and Chr21 represent chromosomes of the female individual; Chr6M, Chr10M and Chr21M represent chromosomes of the male individual. (A) Two inversions in the terminal regions of chromosomes. (B) An inversion in the interior regions of Chr21 and Chr21M. Red bars and red numbers represent the boundaries and their sites of chromosome inversions. Gene ids and functional descriptions with black arrows indicate neighboring genes nearby the boundaries of chromosome inversions.

Table 1. Statistics of the male and female genome assemblies.

	**Female**				**Male**			
	Scaffold		Contig		Scaffold		Contig	
	Length (bp)	Number	Length (bp)	Number	Length (bp)	Number	Length (bp)	Number
**Max length**	55,928,569		15,345,010		52,996,205		19,790,879	
**N50**	29,809,544	11	2,733,495	86	29,536,427	11	7,818,465	31
**N60**	27,447,229	14	2,158,000	119	28,537,785	13	5,896,906	43
**N70**	27,285,240	16	1,744,810	159	26,679,644	16	4,380,229	57
**N80**	25,552,712	19	1,179,287	213	25,622,179	19	3,272,325	77
**N90**	24,145,000	22	638,362	299	23,750,881	22	1,716,882	108
**Total_length**	781,489,634		780,969,649		756,758,225		756,629,725	
**Number>=0 bp**		749		1,796		705		823
**Number>=10000 bp**		428		1,346		683		823
**Number>=20000 bp**		228		1,018		683		823
**GC_rate**	0.4		0.4		0.4		0.4	
**BUSCO**	96.5% [S:93.8%, D:2.7%]				96.1% [S:93.7%, D:2.4%]			

Table 2. Predicted protein-coding genes in the assembled male and female genomes.

Evidence	Method /Species	Female						Male					
		Numbers	Average gene length (bp)	Average CDS length (bp)	Average exon per gene	Average exon length (bp)	Average intron length (bp)	Numbers	Average gene length (bp)	Average CDS length (bp)	Average exon per gene	Average exon length (bp)	Average intron length (bp)
De novo	AUGUSTUS	53,315	7,324	968	5.0	193	1,579	31,518	11,076	1,270	7.0	180	1,627
Homolog	*Danio rerio*	21,631	11,497	1,600	8.7	185	1,292	22,569	10,628	15,945	8.2	195	1,260
	Gasterosteus aculeatus	25,581	9,030	1,264	7.2	177	1,263	26,637	8,306	1,226	6.7	184	1,252
	Oryzias latipes	18,480	11,895	1,773	8.9	198	1,275	20,377	10,680	1,778	8.1	221	1,262
	Takifugu rubripes	19,711	10,606	1,418	7.8	182	1,357	22,446	9,820	1,400	7.4	190	1,325
	Tetraodon nigroviridis	17,565	11,949	1,549	9.1	171	1,288	17,973	11,320	1,543	8.7	176	1,262
	Scleropages formosu	31,620	12,486	1,480	8.0	185	1,575	34,334	10,061	1,305	7.0	186	1,463
Total		25,328	12,358.42	1,584	9.4	167.78	1,276	25,262	11,197	1,546	10.0	154	1,357

References

1. Lake P, Bănărescu P and Banarescu P. Zoogeography of Fresh Waters. Volume 3. Distribution and Dispersal of Freshwater Animals in Africa, Pacific

Areas and South America. Journal of the North American Benthological Society. 1996;15:265. doi:10.2307/1467954.

2. Mu X-d, Song H-m, Wang X-j, Yang Y-x, Luo D, Gu D-e, et al. Genetic variability of the Asian arowana, Scleropages formosus, based on mitochondrial DNA genes. Biochemical Systematics and Ecology. 2012;44:141–8. doi:10.1016/j.bse.2012.04.017.

3. Hilton E and Lavoué S. A review of the systematic biology of fossil and living bony-tongue fishes, Osteoglossomorpha (Actinopterygii: Teleostei). Neotropical Ichthyology. 2018;16 doi:10.1590/1982-0224-20180031.

4. Lavoué S. Testing a time hypothesis in the biogeography of the arowana genus Scleropages (Osteoglossidae). Journal of Biogeography. 2015;42 doi:10.1111/jbi.12585.

5. Greenwood PH, Rosen DE, Weitzman SH, Myers GS, History A and York N. Phyletic studies of teleostean fishes, with a provisional classification of living forms. XF2006174447. 1979;131.

6. Yue GH, Chang A, Alfiko Y and Suwanto A. Current Knowledge on the Biology and Aquaculture of the Endangered Asian Arowana. Reviews in Fisheries Science & Aquaculture. 2019;28:1-18. doi:10.1080/23308249.2019.1697641.

7. Yue GH, Ong D, Wong C, Lim L and Orbán L. A strain-specific and a sex-associated STS marker for Asian arowana (Scleropages formosus, Osteoglossidae). Aquaculture Research. 2003;34:951-7. doi:10.1046/j.1365-2109.2003.00949.x.

8. Shen XY, Kwan HY, Thevasagayam NM, Prakki SR, Kuznetsova IS, Ngoh SY, et al. The first transcriptome and genetic linkage map for Asian arowana. Mol Ecol Resour. 2014;14 3:622-35. doi:10.1111/1755-0998.12212.

9. Yue GH. Current status of genome sequencing and its applications in aquaculture. Aquaculture. 2017;468:337–47. doi:10.1016/j.aquaculture.2016.10.036.

10. Austin CM, Tan MH, Croft LJ, Hammer MP and Gan HM. Whole Genome Sequencing of the Asian Arowana (Scleropages formosus) Provides Insights into the Evolution of Ray-Finned Fishes. Genome Biol Evol. 2015;7 10:2885-95. doi:10.1093/gbe/evv186.

11. Bian C, Hu Y, Ravi V, Kuznetsova IS, Shen X, Mu X, et al. The Asian arowana (Scleropages formosus) genome provides new insights into the evolution of an early lineage of teleosts. Sci Rep. 2016;6:24501. doi:10.1038/srep24501.

12. Goodwin S, McPherson JD and McCombie WR. Coming of age: ten years of next-generation sequencing technologies. Nat Rev Genet. 2016;17 6:333-51. doi:10.1038/nrg.2016.49.

13. Jiao WB and Schneeberger K. The impact of third generation genomic technologies on plant genome assembly. Curr Opin Plant Biol. 2017;36:64-70. doi:10.1016/j.pbi.2017.02.002.

14. Chen Y, Chen Y, Shi C, Huang Z, Zhang Y, Li S, et al. SOAPnuke: a MapReduce acceleration-supported software for integrated quality control and preprocessing of high-throughput sequencing data. Gigascience. 2017;7 1:gix120.

15. Salmela L and Rivals E. LoRDEC: accurate and efficient long read error correction. Bioinformatics. 2014.

16. Wenger AM, Peluso P, Rowell WJ, Chang PC, Hall RJ, Concepcion GT, et al. Accurate circular consensus long-read sequencing improves variant detection and assembly of a human genome. Nat Biotechnol. 2019;37 10:1155-62. doi:10.1038/s41587-019-0217-9.

17. Hon T, Mars K, Young G, Tsai YC, Karalius JW, Landolin JM, et al. Highly accurate long-read HiFi sequencing data for five complex genomes. Sci Data. 2020;7 1:399. doi:10.1038/s41597-020-00743-4.

18. Song L, Bian C, Luo Y, Wang L, You X, Li J, et al. Draft genome of the Chinese mitten crab, Eriocheir sinensis. GigaScience,5,1(2016-01-28). 2016;5 1:5.

19. Kajitani R, Yoshimura D, Okuno M, Minakuchi Y, Kagoshima H, Fujiyama A, et al. Platanus-allee is a de novo haplotype assembler enabling a comprehensive access to divergent heterozygous regions. Nature communications. 2019;10 1:1-15.

20. Ye C, Hill CM, Wu S, Ruan J and Ma ZS. DBG2OLC: Efficient Assembly of Large Genomes Using Long Erroneous Reads of the Third Generation Sequencing Technologies. Sci Rep. 2016;6:31900. doi:10.1038/srep31900.

21. Li H. Minimap2: pairwise alignment for nucleotide sequences. Bioinformatics. 2018; 18:18.

22. Vaser R, Sović I, Nagarajan N and Šikić M. Fast and accurate de novo genome assembly from long uncorrected reads. Genome research. 2017;27 5:737-46. doi:10.1101/gr.214270.116.

23. Walker BJ, Abeel T, Shea T, Priest M, Abouelliel A, Sakthikumar S, et al. Pilon: an integrated tool for comprehensive microbial variant detection and genome assembly improvement. PloS one. 2014;9 11:e112963.

24. Boetzer M and Pirovano W. SSPACE-LongRead: scaffolding bacterial draft genomes using long read sequence information. Bmc Bioinformatics. 2014;15.

25. Boetzer M, Henkel CV, Jansen HJ, Butler D and Pirovano W. Scaffolding pre-assembled contigs using SSPACE. Bioinformatics. 2011;27 4:578-9.

26. Wang D, Chen X, Zhang X, Li J, Yi Y, Bian C, et al. Whole Genome Sequencing of the Giant Grouper (Epinephelus lanceolatus) and High-Throughput Screening of Putative Antimicrobial Peptide Genes. Mar Drugs. 2019;17 9 doi:10.3390/md17090503.

27. Smith JJ, Timoshevskaya N, Ye C, Holt C, Keinath MC, Parker HJ, et al. The sea lamprey germline genome provides insights into programmed genome rearrangement and vertebrate evolution. Nat Genet. 2018;50 2:270-7. doi:10.1038/s41588-017-0036-1.

28. Durand NC, Shamim MS, Machol I, Rao SS, Huntley MH, Lander ES, et al. Juicer provides a one-click system for analyzing loop-resolution Hi-C experiments. Cell systems. 2016;3 1:95-8.

29. Dudchenko O, Batra SS, Omer AD, Nyquist SK, Hoeger M, Durand NC, et al. De novo assembly of the Aedes aegypti genome using Hi-C yields chromosome-length scaffolds. Science. 2017;356 6333:92-5.

30. Ruan J and Li H. Fast and accurate long-read assembly with wtdbg2. Nat Methods. 2020;17 2:155-8. doi:10.1038/s41592-019-0669-3.

31. Smit A, Hubley R and Green P. RepeatMasker Open-4.0. 2013-2015< http://www. repeatmasker. org>. 2019.

32. Bao W, Kojima KK and Kohany O. Repbase Update, a database of repetitive elements in eukaryotic genomes. BioMed Central % Journal Article; 2015.

33. Zhao X and Hao W. LTR_FINDER: an efficient tool for the prediction of full-length LTR retrotransposons. Nucleic Acids Research. 2007;35 Web Server issue:W265-8.

34. Benson G. Tandem repeats finder: a program to analyze DNA sequences. Oxford University Press % Journal Article; 1999.

35. Bruna T, Hoff KJ, Lomsadze A, Stanke M and Borodovsky M. BRAKER2: automatic eukaryotic genome annotation with GeneMark-EP+ and AUGUSTUS supported by a protein database. NAR Genom Bioinform. 2021;3 1:lqaa108. doi:10.1093/nargab/lqaa108.

36. Bian C, Hu Y, Ravi V, Kuznetsova IS, Shen X, Mu X, et al. The Asian arowana (Scleropages formosus) genome provides new insights into the evolution of an early lineage of teleosts. Scientific reports. 2016;6 1:1-17.

37. Kanehisa M, Furumichi M, Tanabe M, Sato Y and Morishima K. KEGG: new perspectives on genomes, pathways, diseases and drugs. Nucleic Acids Res. 2017;45 D1:D353-d61. doi:10.1093/nar/gkw1092.

38. Bairoch A, Apweiler R, Wu CH, Barker WC, Boeckmann B, Ferro S, et al. The universal protein resource (UniProt). Nucleic acids research. 2005;33 suppl_1:D154-D9.

39. Jones P, Binns D, Chang HY, Fraser M, Li W, McAnulla C, et al. InterProScan 5: genome-scale protein function classification. Bioinformatics. 2014;30 9:1236-40. doi:10.1093/bioinformatics/btu031.

40. Zdobnov EM and Apweiler R. InterProScan–an integration platform for the signature-recognition methods in InterPro. Bioinformatics. 2001;17 9:847-8.

41. Thomas PD, Campbell MJ, Kejariwal A, Mi H, Karlak B, Daverman R, et al. PANTHER: a library of protein families and subfamilies indexed by function. Genome Res. 2003;13 9:2129-41. doi:10.1101/gr.772403.

42. Bateman A, Coin L, Durbin R, Finn RD, Hollich V, Griffiths-Jones S, et al. The Pfam protein families database. Nucleic acids research. 2004;32 suppl_1:D138-D41.

43. Attwood TK, Croning MDR, Flower DR, Lewis AP, Mabey JE, Scordis P, et al. PRINTS-S: the database formerly known as PRINTS. Nucleic Acids Research. 2000;28 1:225-7. doi:10.1093/nar/28.1.225 %J Nucleic Acids Research.

44. Servant F, Bru C, Carrère S, Courcelle E, Gouzy J, Peyruc D, et al. ProDom: automated clustering of homologous domains. Brief Bioinform. 2002;3 3:246-51. doi:10.1093/bib/3.3.246.

45. Sigrist CJ, Cerutti L, de Castro E, Langendijk-Genevaux PS, Bulliard V, Bairoch A, et al. PROSITE, a protein domain database for functional characterization and annotation. Nucleic Acids Res. 2010;38 Database issue:D161-6. doi:10.1093/nar/gkp885.

46. Letunic I, Doerks T and Bork P. SMART 7: recent updates to the protein domain annotation resource. Nucleic Acids Res. 2012;40 Database issue:D302-5. doi:10.1093/nar/gkr931.

47. Ashburner M, Ball CA, Blake JA, Botstein D, Butler H, Cherry JM, et al. Gene ontology: tool for the unification of biology. Nature genetics. 2000;25 1:25-9.

48. Kim D, Langmead B and Salzberg SL. HISAT: a fast spliced aligner with low memory requirements. Nature methods. 2015;12 4:357-60. doi:10.1038/nmeth.3317.

49. Trapnell C, Hendrickson DG, Sauvageau M, Goff L, Rinn JL and Pachter L. Differential analysis of gene regulation at transcript resolution with RNA-seq. Nature biotechnology. 2013;31 1:46-53. doi:10.1038/nbt.2450.

50. Robinson MD, McCarthy DJ and Smyth GK. edgeR: a Bioconductor package for differential expression analysis of digital gene expression data. Bioinformatics. 2010;26 1:139-40. doi:10.1093/bioinformatics/btp616.

51. Li H and Durbin R. Fast and accurate short read alignment with Burrows-Wheeler transform. Bioinformatics. 2009;25 14:1754-60. doi:10.1093/bioinformatics/btp324.

52. Mckenna A, Hanna M, Banks E, Sivachenko A, Cibulskis K, Kernytsky A, et al. The Genome Analysis Toolkit: a MapReduce framework for analyzing next-generation DNA sequencing data. Genome Research. 2010;20 9:1297-303.

53. Kang HM, Sul JH, Service SK, Zaitlen NA, Kong S-y, Freimer NB, et al. Variance component model to account for sample structure in genome-wide association studies. Nature genetics. 2010;42 4:348-54.

54. Purcell S, Neale B, Todd-Brown K, Thomas L, Ferreira MA, Bender D, et al. PLINK: a tool set for whole-genome association and population-based linkage analyses. The American journal of human genetics. 2007;81 3:559-75.

55. Turner SD. qqman: an R package for visualizing GWAS results using QQ and manhattan plots. Biorxiv. 2014:005165.

56. Marçais G, Delcher AL, Phillippy AM, Coston R, Salzberg SL and Zimin AJPcb. MUMmer4: A fast and versatile genome alignment system. 2018;14 1:e1005944.

57. Li H. Minimap2: pairwise alignment for nucleotide sequences. Bioinformatics. 2018;34 18:3094-100. doi:10.1093/bioinformatics/bty191.

58. Thorvaldsdottir H, Robinson JT and Mesirov JP. Integrative Genomics Viewer (IGV): high-performance genomics data visualization and exploration. Brief Bioinform. 2013;14 2:178-92. doi:10.1093/bib/bbs017.

59. Simao FA, Waterhouse RM, Ioannidis P, Kriventseva EV and Zdobnov EM. BUSCO: assessing genome assembly and annotation completeness with single-copy orthologs. Bioinformatics. 2015;31 19:3210-2. doi:10.1093/bioinformatics/btv351.

60. Dougherty GW, Mizuno K, Nothe-Menchen T, Ikawa Y, Boldt K, Ta-Shma A, et al. CFAP45 deficiency causes situs abnormalities and asthenospermia by disrupting an axonemal adenine nucleotide homeostasis module. Nat Commun. 2020;11 1:5520. doi:10.1038/s41467-020-19113-0.

Manuscript

Click here to access/download
Supplementary Material
Supplementary Material 20220626.docx
