# Supplementary material for: Identification of candidate sex‐specific genomic regions in male and female Asian arowana genomes
Source: Gigascience. 2022 Sep 15;11:giac085. doi: 10.1093/gigascience/giac085 (PMC9475665; doi:10.1093/gigascience/giac085)
Supplement: giac085_Supplemental_File [file giac085_supplemental_file.docx]

**
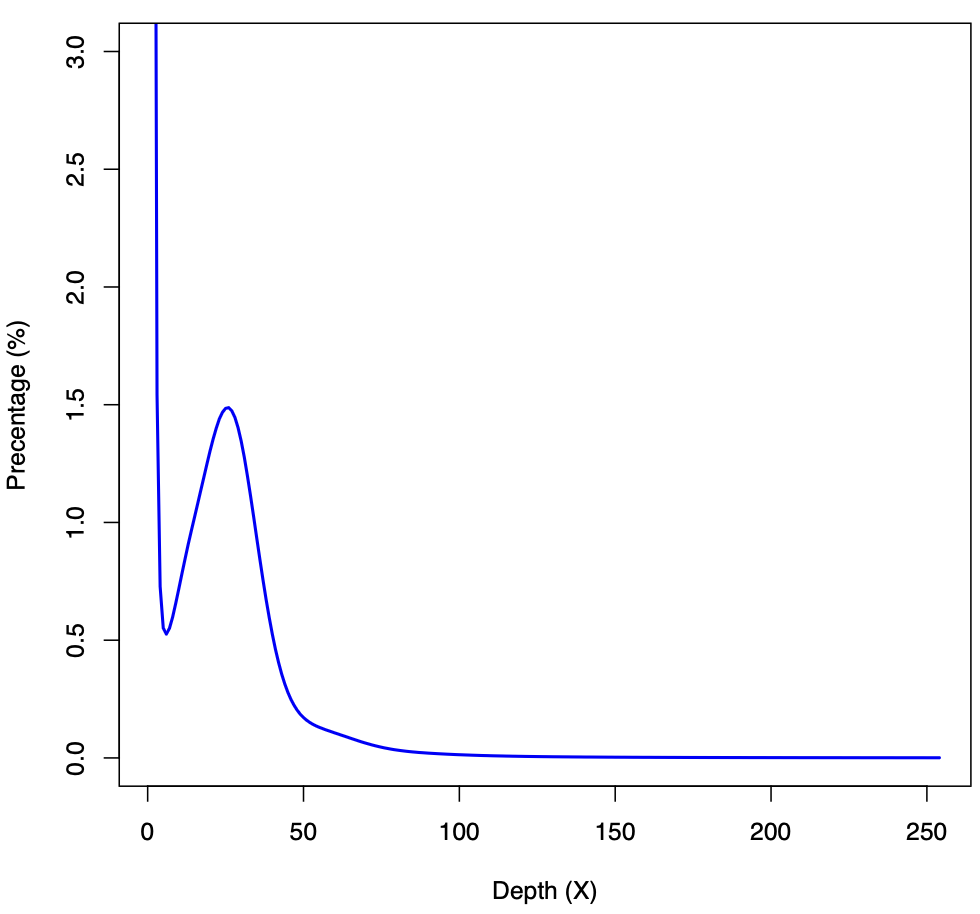
**

**Supplementary Figure 1. 17-kmer analysis for prediction of genome size of the**

**female individual.** Its genome size was estimated at 0.82 Gb. The peak coverage of this curve is 26.

**
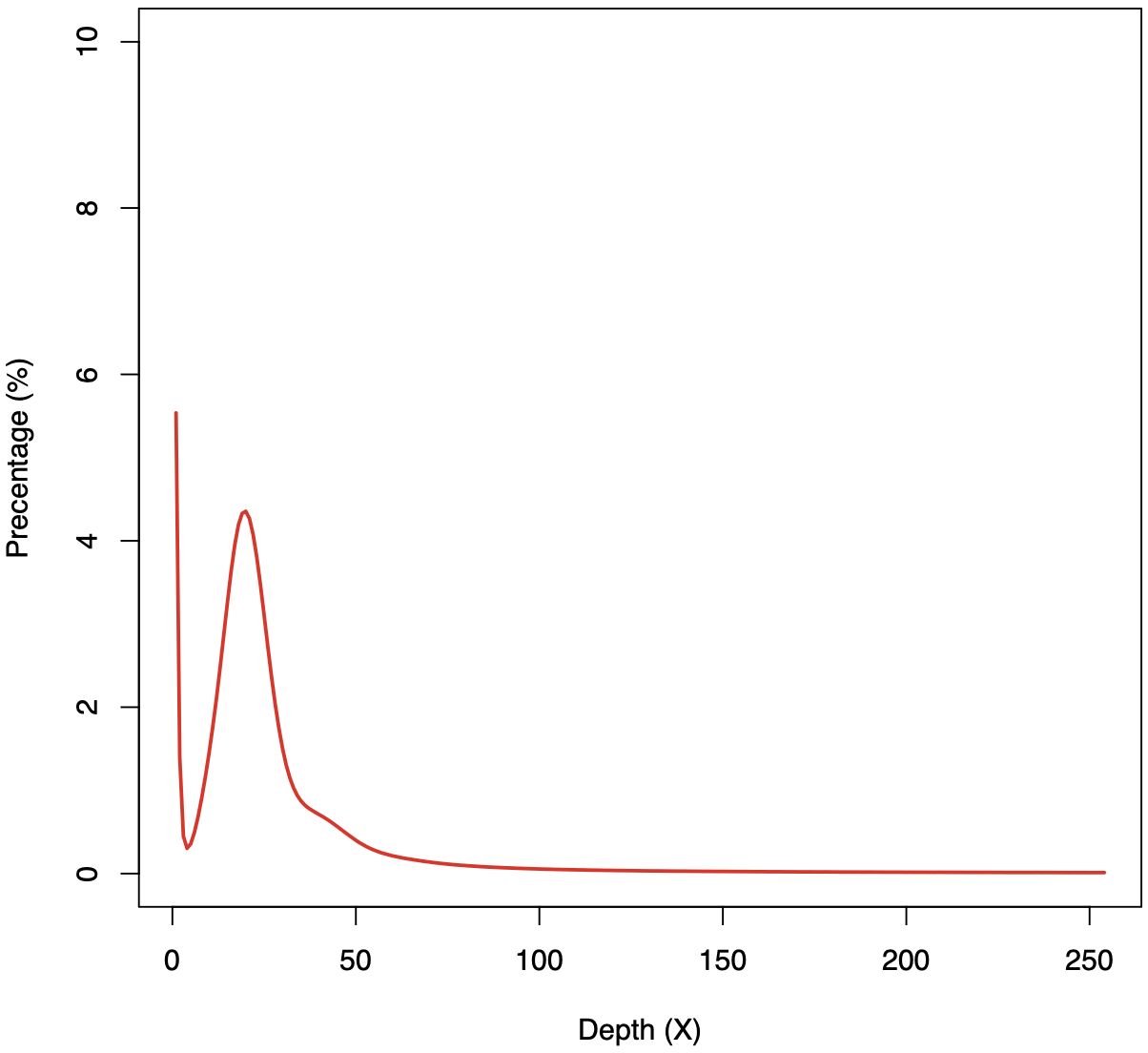
**

**Supplementary Figure 2. 17-kmer analysis for prediction of genome size of the**

**male individual.** Its genome size was estimated at 0.85 Gb. The peak coverage of this curve is 20.


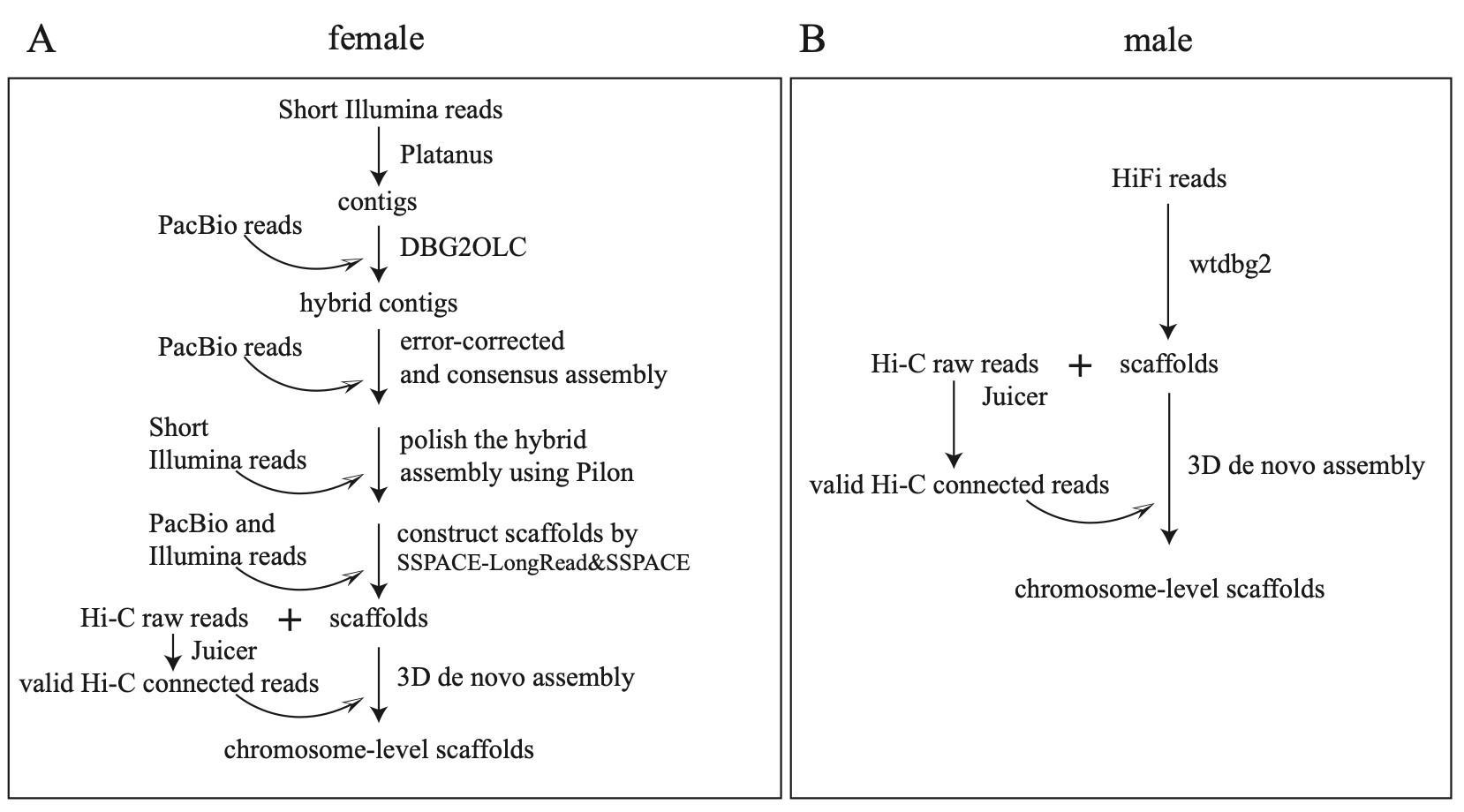


**Supplementary Figure 3. Detailed assembling pipelines of female and male.**

**
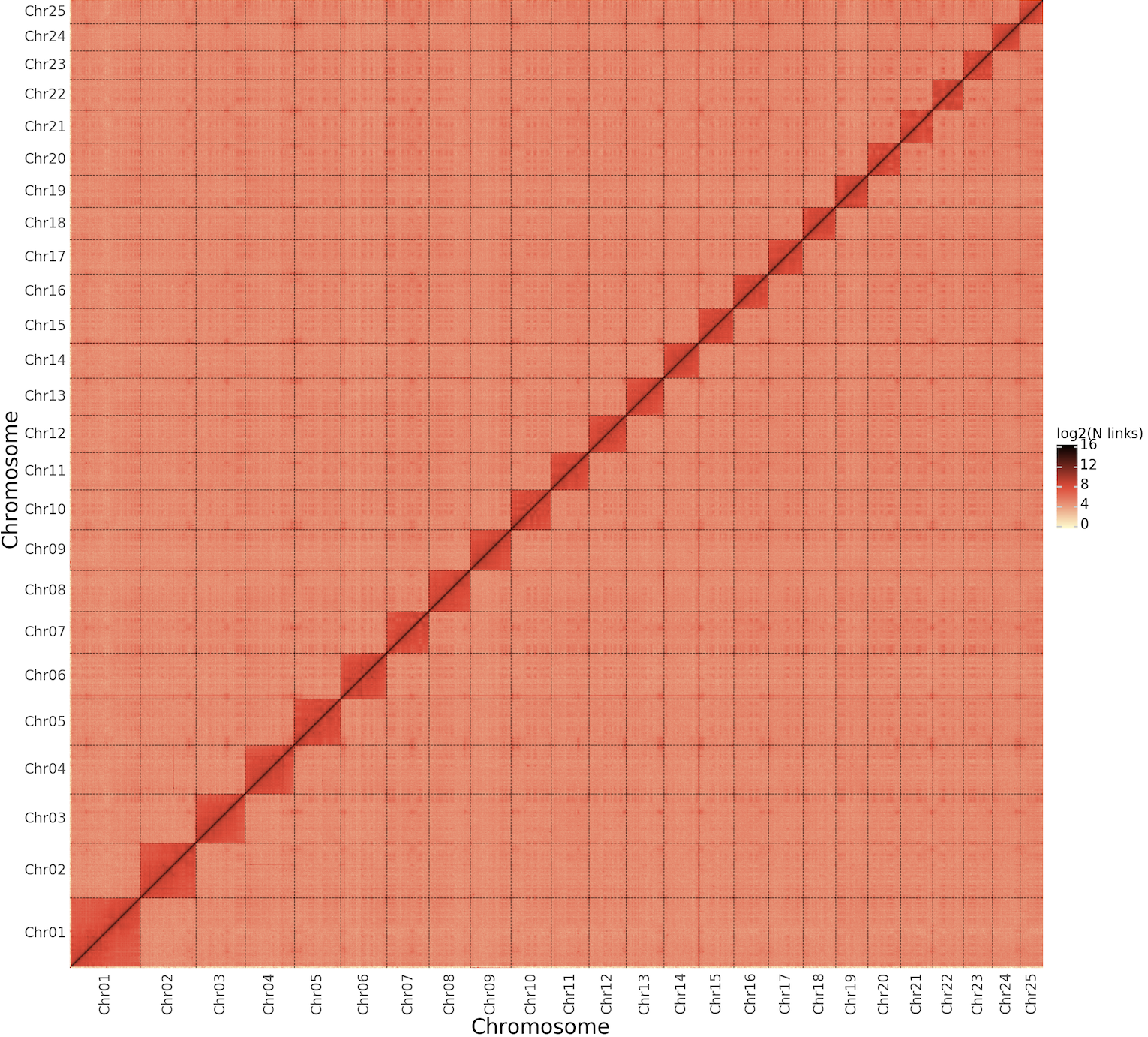
**

**Supplementary Figure 4. Heatmap of the Hi-C result of female individual.**


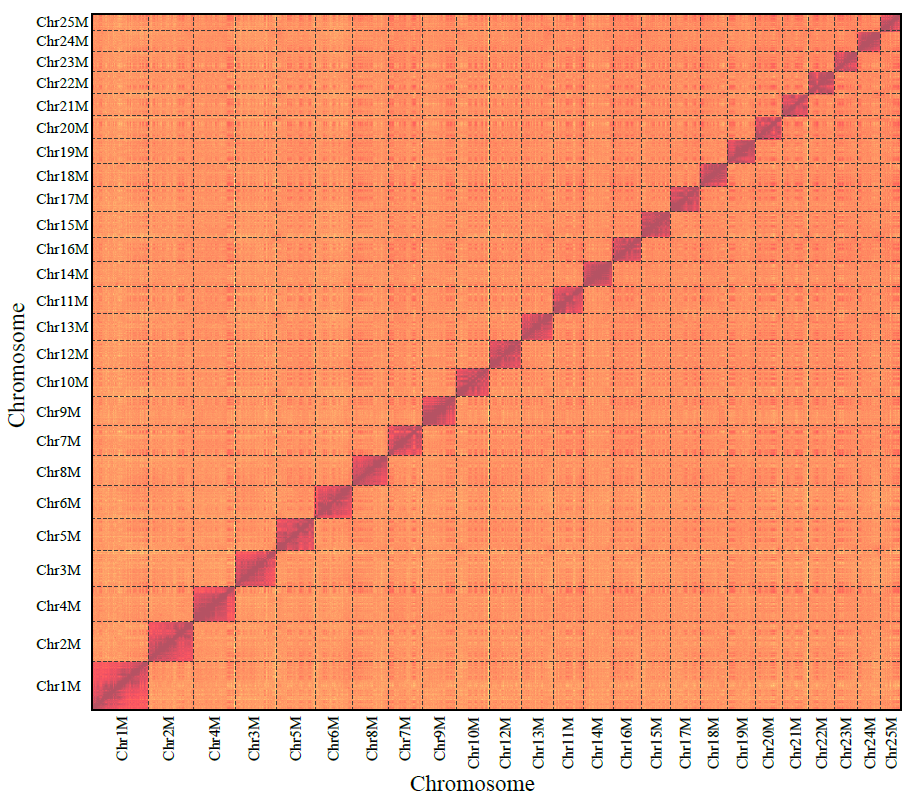


**Supplementary Figure 5. Heatmap of the Hi-C result of male individual.**

**
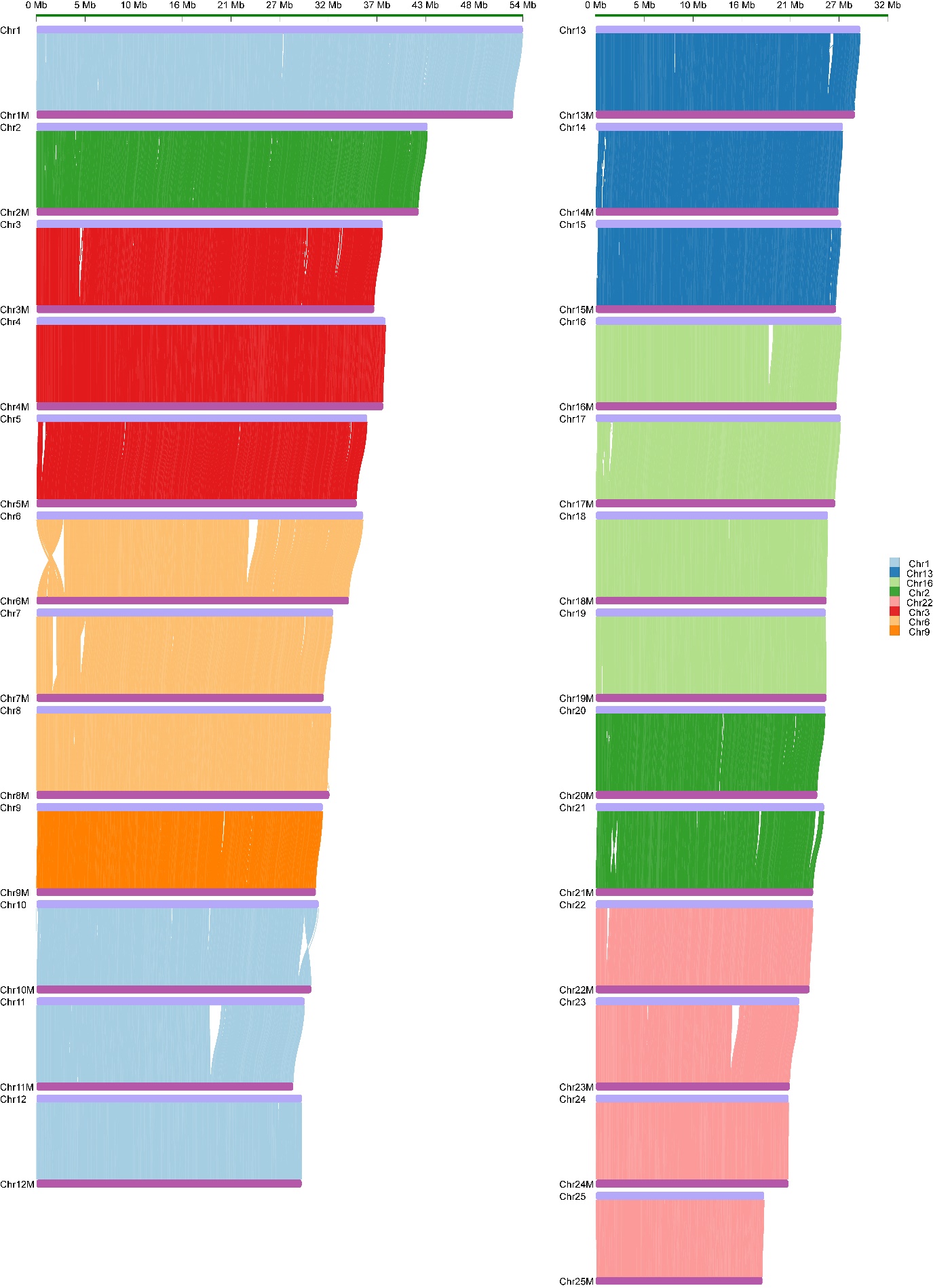
**

**Supplementary Figure 6. Chromosomal alignments of male and female chromosomes.**

**
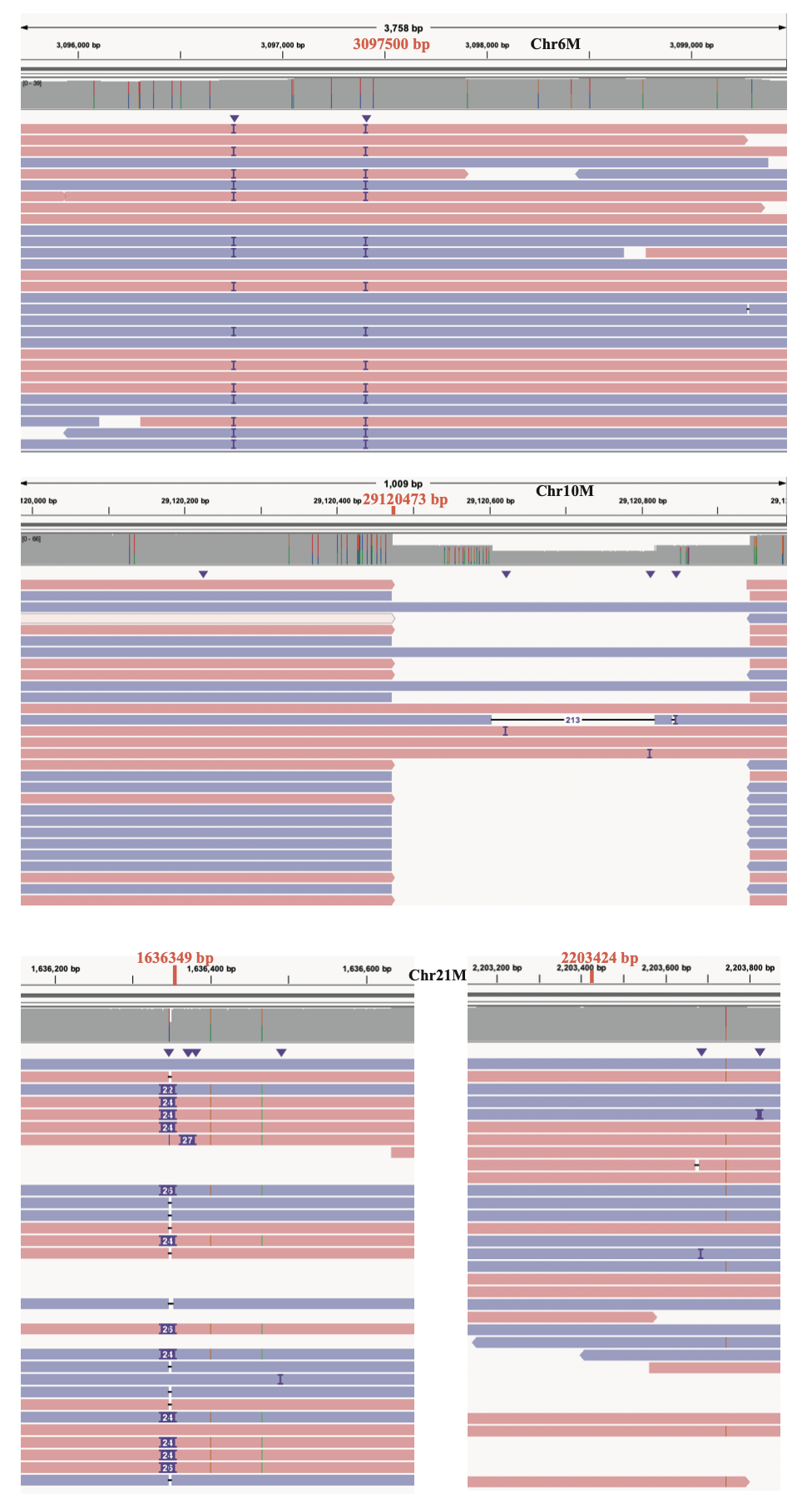
**

**Supplementary Figure 7. Integrated Genome Visualisation screenshot of inversion boundaries of Chr6M, Chr10M and Chr21M chromosomes.** Red numbers were the inversion boundary sites. Blue and red lines represent the mapped Pacbio HiFi reads.

**
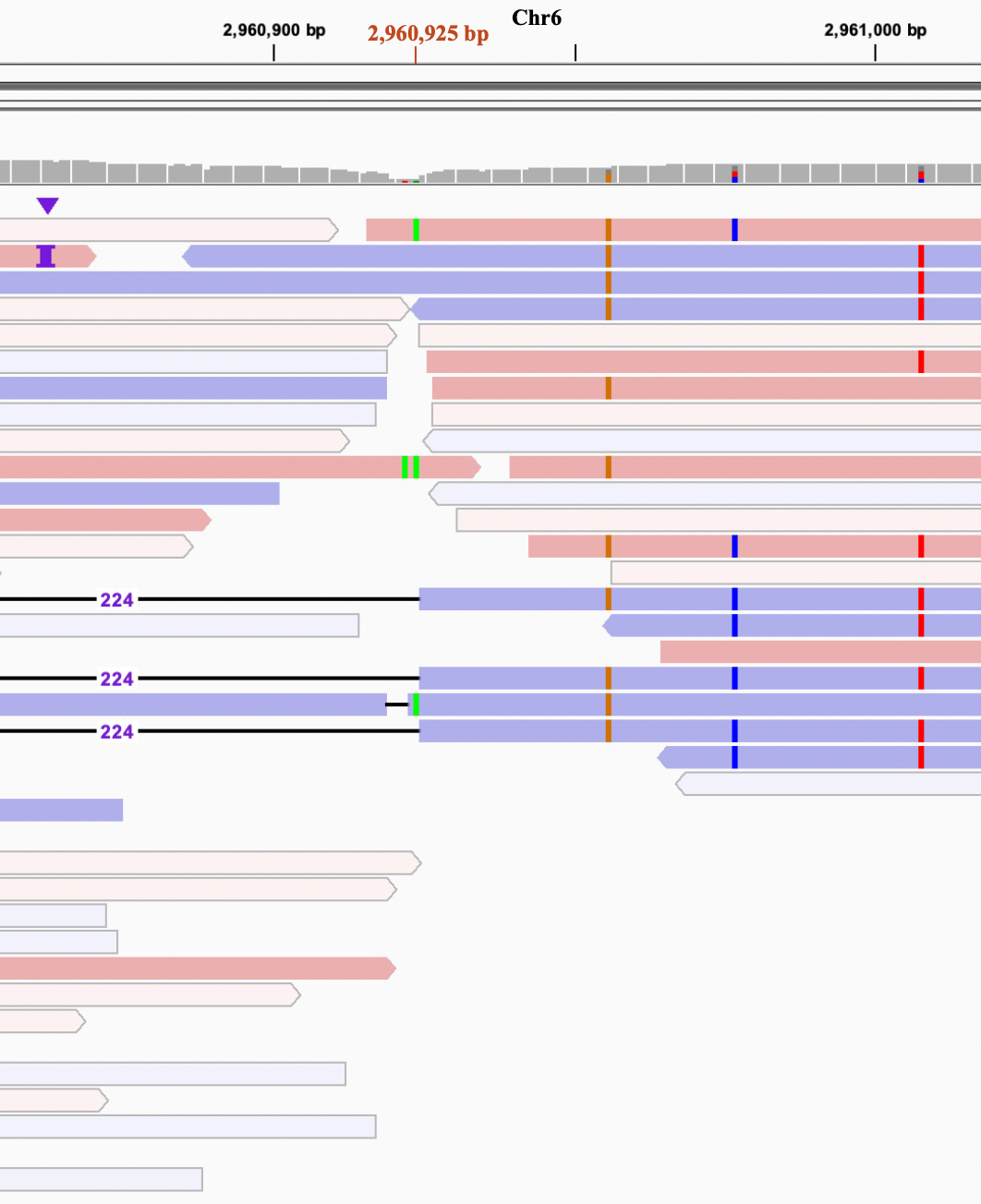
**

**
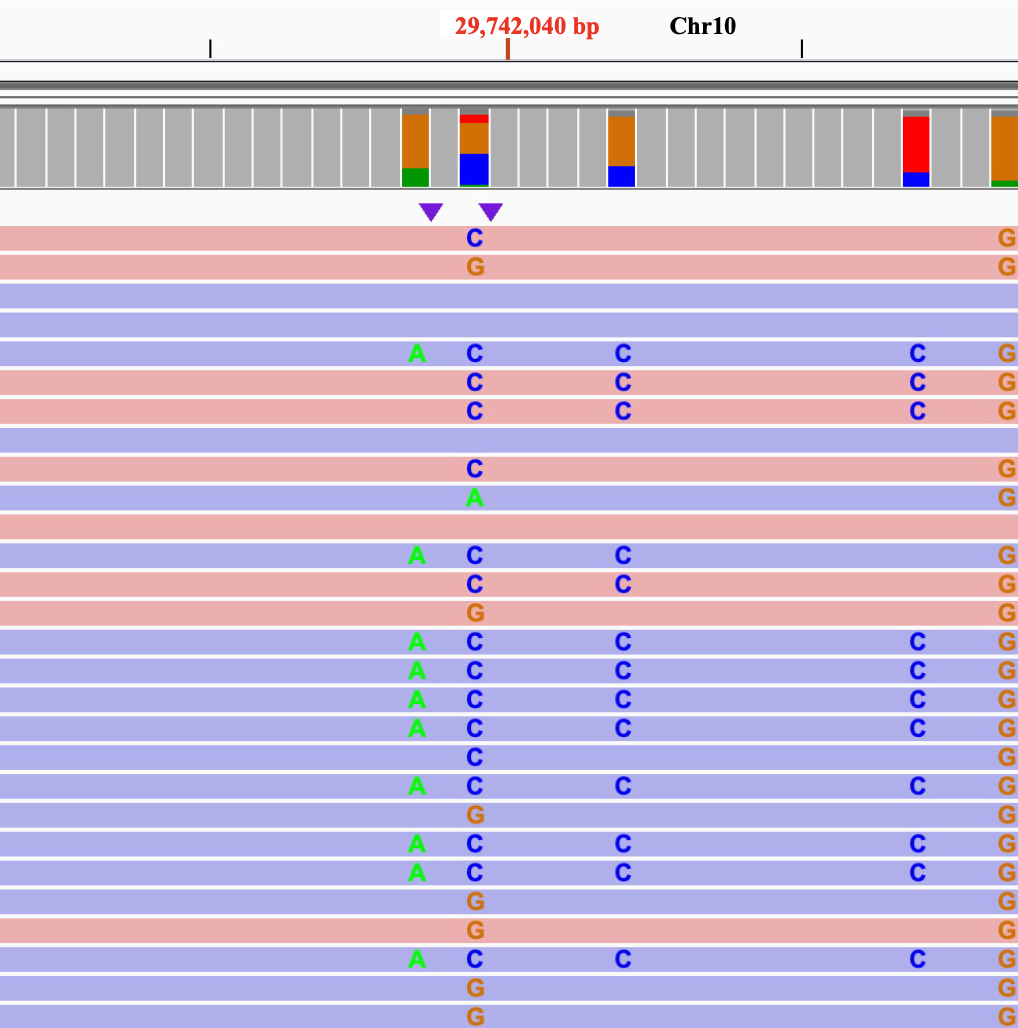
**

**
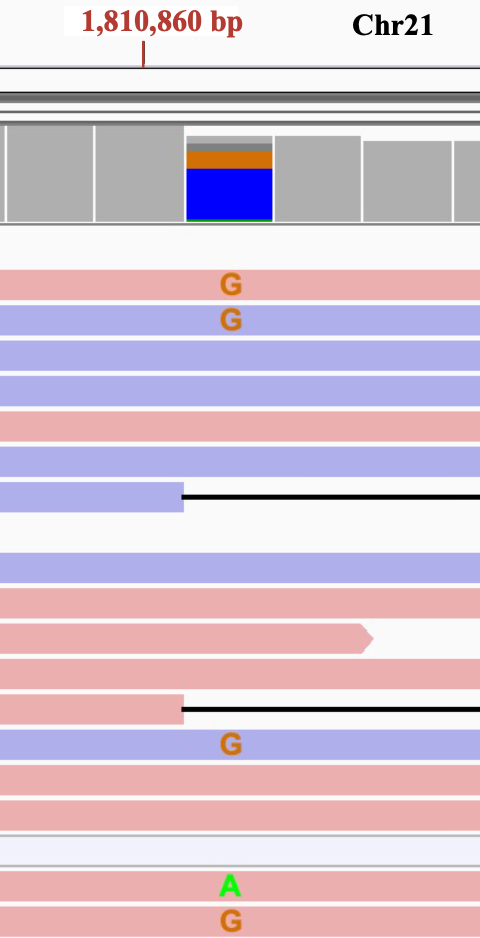

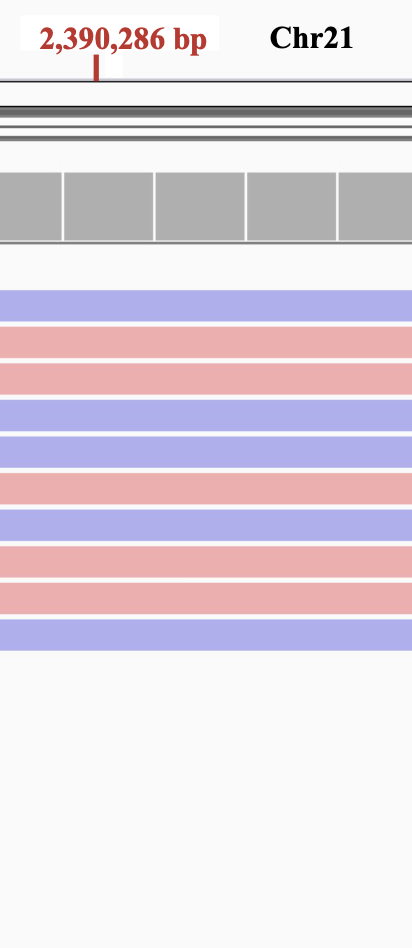
**

**Supplementary Figure 8. Integrated Genome Visualisation screenshot of inversion boundaries of Chr6, Chr10 and Chr21 chromosomes.** Red numbers were the inversion boundary sites. Blue and red lines represent the mapped Pacbio reads.

**Supplementary Table 1. Summary of sequenced reads for male and female genomes.**

| **Sex** | **Sequence platform** | **Insert size** | **Raw bases (Gb)** | **Clean bases (Gb)** |
| --- | --- | --- | --- | --- |
| Female | Pacbio | 20k | 36.0 | 27.4 |
|  | Illumina | 170bp | 24.7 | 19.7 |
|  |  | 500bp | 20.3 | 16.1 |
|  |  | 800bp | 13.8 | 9.6 |
|  |  | 2k | 22.5 | 15.5 |
|  |  | 5k | 10.6 | 6.0 |
|  |  | 10k | 9.9 | 4.7 |
|  |  | 20k | 4.7 | 1.8 |
| Female HiC | Illumina | - | 80.7 | - |
| Male | Pacbio (HiFi) | 20k | 26.5 | - |
| Male HiC | Illumina | - | 179.2 | - |

**Supplementary Table 2.** **Summary of map ratio for the 30 male and female samples.**

| Libraries | Raw bases (Gb) | Clean bases (Gb) | Mapped bases (Gb) | Mapped ratio (%) | Sex |
| --- | --- | --- | --- | --- | --- |
| wHAXPI043368-76 | 22.9 | 20.3 | 19.0 | 83.2 | female |
| wHAXPI047321-15 | 26.8 | 24.7 | 23.3 | 86.8 | female |
| wHAXPI043373-75 | 25.1 | 22.4 | 21.4 | 85.2 | female |
| wHAXPI043374-78 | 24.4 | 22.0 | 20.6 | 84.4 | female |
| wHAXPI043378-80 | 22.1 | 19.8 | 18.5 | 84.0 | female |
| wHAXPI047322-37 | 24.6 | 23.0 | 21.4 | 86.8 | female |
| wHAXPI046607-85 | 24.4 | 22.0 | 20.2 | 82.9 | female |
| wHAXPI046620-84 | 26.2 | 23.7 | 22.0 | 83.9 | female |
| wHAXPI047283-16 | 22.9 | 21.4 | 20.2 | 88.0 | female |
| wHAXPI047286-24 | 25.9 | 22.7 | 22.2 | 85.5 | female |
| wHAXPI047309-11 | 21.4 | 19.9 | 18.8 | 87.9 | female |
| wHAXPI047311-30 | 23.5 | 21.9 | 20.3 | 86.3 | female |
| wHAXPI047315-23 | 29.6 | 26.8 | 25.6 | 86.4 | female |
| wHAXPI047319-32 | 23.1 | 21.7 | 20.0 | 86.7 | female |
| wHAXPI046644-83 | 25.5 | 23.0 | 21.5 | 84.3 | female |
| wHAXPI043365-83 | 21.1 | 18.9 | 18.0 | 85.5 | male |
| wHAXPI043366-84 | 20.6 | 18.4 | 17.3 | 84.2 | male |
| wHAXPI043376-79 | 19.3 | 17.1 | 16.3 | 84.6 | male |
| wHAXPI043381-86 | 20.4 | 18.2 | 17.2 | 84.4 | male |
| wHAXPI043383-77 | 23.9 | 20.9 | 19.6 | 82.1 | male |
| wHAXPI043384-87 | 23.4 | 21.1 | 20.4 | 87.1 | male |
| wHAXPI047290-26 | 23.5 | 20.9 | 20.1 | 85.7 | male |
| wHAXPI047292-17 | 24.2 | 22.4 | 21.2 | 87.5 | male |
| wHAXPI047299-18 | 22.3 | 20.6 | 19.5 | 87.5 | male |
| wHAXPI047304-21 | 25.5 | 23.3 | 22.0 | 86.3 | male |
| wHAXPI047305-13 | 28.0 | 25.6 | 24.0 | 85.5 | male |
| wHAXPI047306-8 | 22.1 | 20.6 | 19.2 | 86.7 | male |
| wHAXPI047307-14 | 28.2 | 25.8 | 24.8 | 87.9 | male |
| wHAXPI047316-12 | 25.3 | 23.3 | 21.9 | 86.7 | male |
| wHAXPI047320-9 | 25.9 | 22.5 | 21.1 | 81.4 | male |

**Supplementary Table 3. Repetitive elements in the assembled genome of female individual.**

| Type | Repbase TEs | | TE proteins | | *De novo* | | Combined TEs | |
| --- | --- | --- | --- | --- | --- | --- | --- | --- |
|  | Length (bp) | % in genome | Length (bp) | % in genome | Length (bp) | % in genome | Length (bp) | % in genome |
| DNA | 42,384,960 | 5.4 | 599,916 | 0.1 | 40,713,941 | 5.2 | 69,425,706 | 8.9 |
| LINE | 32,749,014 | 4.2 | 25,288,682 | 3.2 | 121,407,716 | 15.5 | 129,160,932 | 16.5 |
| SINE | 17,543,728 | 2.2 | 0 | 0.0 | 5,532,654 | 0.7 | 22,222,630 | 2.8 |
| LTR | 11,980,146 | 1.5 | 9,903,737 | 1.3 | 90,664,834 | 11.6 | 93,640,806 | 12.0 |
| Other | 11,678 | 0.0 | 0 | 0.0 | 0 | 0.0 | 11,678 | 0.0 |
| Unknown | 0 | 0.0 | 0 | 0.0 | 1,824,263 | 0.2 | 1,824,263 | 0.2 |
| Total | 94,068,254 | 12.0 | 35,776,743 | 4.6 | 209,111,564 | 26.8 | 217,364,929 | 27.8 |

**Supplementary Table 4. Repetitive elements in the assembled genome of male individual.**

| Type | Repbase TEs | | TE proteins | | *De novo* | | Combined TEs | |
| --- | --- | --- | --- | --- | --- | --- | --- | --- |
|  | Length (bp) | % in genome | Length (bp) | % in genome | Length (bp) | % in genome | Length (bp) | % in genome |
| DNA | 44,417,472 | 5.9 | 5,490,486 | 0.7 | 98,588,225 | 13.0 | 117,797,764 | 15.6 |
| LINE | 36,556,175 | 4.8 | 30,681,753 | 4.1 | 120,032,816 | 15.9 | 134,381,156 | 17.8 |
| SINE | 18,743,350 | 2.5 | 0 | 0.0 | 31,232,786 | 4.1 | 42,410,377 | 5.6 |
| LTR | 14,169,952 | 1.9 | 11,055,236 | 1.5 | 93,815,257 | 12.4 | 98,646,229 | 13.0 |
| Other | 16045 | 0.0 | 0 | 0.0 | 0 | 0.0 | 16,045 | 0.0 |
| Unknown | 0 | 0.0 | 0 | 0.0 | 14,761,747 | 2.0 | 14,761,747 | 2.0 |
| Total | 105,089,618 | 13.9 | 47,124,008 | 6.2 | 246,231,180 | 32.5 | 252,961,575 | 33.4 |

**Supplementary Table 5. Chromosome location of SNPs.**

| Chromosome | Total | Intergenic | CDS | | |  | |
| --- | --- | --- | --- | --- | --- | --- | --- |
|  |  |  | Total | Synonymous | Non-Synonymous | | Intron |
| Chr1 | 653,412 | 398,298 | 17,312 | 10,694 | 6,618 | | 237,802 |
| Chr2 | 522,180 | 316,604 | 12,801 | 7,770 | 5,031 | | 192,775 |
| Chr3 | 472,925 | 284,509 | 22,925 | 6,817 | 16,108 | | 165,491 |
| Chr4 | 434,120 | 252,430 | 23,863 | 6,778 | 17,085 | | 157,827 |
| Chr5 | 447,562 | 271,570 | 9,868 | 6,133 | 3,735 | | 166,124 |
| Chr6 | 461,273 | 281,617 | 13,025 | 7,519 | 5,506 | | 166,631 |
| Chr7 | 345,183 | 201,302 | 11,042 | 6,556 | 4,486 | | 132,839 |
| Chr8 | 397,110 | 243,146 | 11,462 | 6,672 | 4,790 | | 142,502 |
| Chr9 | 383,554 | 232,414 | 10,063 | 5,729 | 4,334 | | 141,077 |
| Chr10 | 360,838 | 225,310 | 10,641 | 6,272 | 4,369 | | 124,887 |
| Chr11 | 342,505 | 208,663 | 8,894 | 5,273 | 3,621 | | 124,948 |
| Chr12 | 349,640 | 219,690 | 9,353 | 5,627 | 3,726 | | 120,597 |
| Chr13 | 346,090 | 233,607 | 8,784 | 5,051 | 3,733 | | 103,699 |
| Chr14 | 311,621 | 208,702 | 7,097 | 4,348 | 2,749 | | 95,822 |
| Chr15 | 296,890 | 186,683 | 7,466 | 4,376 | 3,090 | | 102,741 |
| Chr16 | 290,725 | 169,449 | 9,257 | 5,400 | 3,857 | | 112,019 |
| Chr17 | 306,448 | 182,399 | 7,547 | 4,586 | 2,961 | | 116,502 |
| Chr18 | 302,609 | 187,776 | 6,944 | 4,178 | 2,766 | | 107,889 |
| Chr19 | 332,470 | 195,662 | 9,211 | 5,541 | 3,670 | | 127,597 |
| Chr20 | 295,685 | 172,571 | 9,929 | 5,719 | 4,210 | | 113,185 |
| Chr21 | 277,062 | 163,698 | 10,512 | 5,244 | 5,268 | | 102,852 |
| Chr22 | 273,376 | 162,500 | 7,023 | 4,333 | 2,690 | | 103,853 |
| Chr23 | 255,368 | 158,502 | 7,876 | 4,672 | 3,204 | | 88,990 |
| Chr24 | 242,201 | 143,980 | 5,848 | 3,612 | 2,236 | | 92,373 |
| Chr25 | 217,912 | 130,825 | 6,276 | 3,746 | 2,530 | | 80,811 |
| Total | 8,918,759 | 5,431,907 | 265,019 | 142,646 | 122,373 | | 3,221,833 |

**Supplementary Table 6. Genes in potential sex divergence regions in chromosomes predicted by the GWAS and their expression values (FPKM) in ovary and testis tissues.**

| Gene ID | Chr | Gene symbol | Ovary1 | Ovary2 | Ovary3 | Testis1 | Testis2 | Testis3 |
| --- | --- | --- | --- | --- | --- | --- | --- | --- |
| jg33400.t1 | Chr12 | *ncam2* | 0.03 | 0.04 | 0.01 | 0.14 | 0.03 | 0.04 |
| jg33600.t1 | Chr14 | *cd48* | 0 | 0.02 | 0 | 0 | 0.04 | 0.11 |
| jg43012.t1 | Chr18 | *wscd2* | 0 | 0.04 | 0 | 0.51 | 0.43 | 0.74 |
| jg13369.t1 | Chr19 | *cfap52* | 0.25 | 0.28 | 0.51 | 25.39 | 25.06 | 6.78 |
| jg13247.t1 | Chr19 | *cep95* | 5.50 | 18.40 | 16.74 | 2.75 | 2.81 | 4.34 |
| jg13148.t1 | Chr19 | *ca10* | 0 | 0.024 | 0 | 0.48 | 0.32 | 0.38 |
| jg21466.t1 | Chr19 | *mrtfb* | 0.26 | 1.46 | 1.18 | 2.21 | 3.32 | 2.05 |
| jg11085.t1 | Chr21 | *itga4* | 0.12 | 0.11 | 0.06 | 0.52 | 0.54 | 0.32 |

**Supplementary Table 7. Statistics of the mapped ratio of male and female chromosomes.**

| Chromosome of Female | The mapped regions/chromosome length | Chromosome of male | The mapped regions/chromosome length |
| --- | --- | --- | --- |
| Chr1 | 0.93 | Chr1M | 0.98 |
| Chr2 | 0.96 | Chr2M | 0.98 |
| Chr3 | 0.92 | Chr3M | 0.97 |
| Chr4 | 0.98 | Chr4M | 0.99 |
| Chr5 | 0.94 | Chr5M | 0.98 |
| Chr6 | 0.93 | Chr6M | 0.97 |
| Chr7 | 0.96 | Chr7M | 0.99 |
| Chr8 | 0.97 | Chr8M | 0.98 |
| Chr9 | 0.96 | Chr9M | 0.98 |
| Chr10 | 0.95 | Chr10M | 0.98 |
| Chr11 | 0.93 | Chr11M | 0.97 |
| Chr12 | 0.98 | Chr12M | 0.98 |
| Chr13 | 0.96 | Chr13M | 0.98 |
| Chr14 | 0.96 | Chr14M | 0.98 |
| Chr15 | 0.95 | Chr15M | 0.97 |
| Chr16 | 0.97 | Chr16M | 0.98 |
| Chr17 | 0.96 | Chr17M | 0.98 |
| Chr18 | 0.98 | Chr18M | 0.98 |
| Chr19 | 0.98 | Chr19M | 0.97 |
| Chr20 | 0.94 | Chr20M | 0.98 |
| Chr21 | 0.94 | Chr21M | 0.99 |
| Chr22 | 0.97 | Chr22M | 0.98 |
| Chr23 | 0.94 | Chr23M | 0.98 |
| Chr24 | 0.98 | Chr24M | 0.98 |
| Chr25 | 0.97 | Chr25M | 0.98 |
