# Supplementary material for: Identification of candidate sex‐specific genomic regions in male and female Asian arowana genomes
Source: Gigascience. 2022 Sep 15;11:giac085. doi: 10.1093/gigascience/giac085 (PMC9475665; doi:10.1093/gigascience/giac085)
Supplement: giac085_Response_to_Reviewer_Comments_Original_Submission [file giac085_response_to_reviewer_comments_original_submission.pdf]

Author's Response To Reviewer Comments

Close

Dear editor,
Thanks for your nice advice and the two reviewers' comments. According to your instructive opinions, we sequenced additional PacBio HiFI and Hi-C reads for the male arowana, and then performed a series of new analyses. We also made careful revisions throughout the manuscript. Our revised manuscript (changes are highlighted in yellow) and point-by-point responses are uploaded for your consideration.

Reviewer #1:
Title: Sex-specific genomic region identification in male and female Asian arowana genomes
SUMMARY
This manuscript presents a genomics analysis of a fish species, Scleropages formosus, for which there was only a draft genome previously. The authors present a high-quality female genome assembly and lower quality male genome, plus gene annotation, repeats analysis, BUSCO, transcriptomics, GWAS, and comparative genomics. These data helped identify some candidates for the sex-specific regions which remain elusive.
MAJOR ISSUE
The paper says the annotation files are available at NCBI. However, the NCBI pages say, "No public data is linked to this project." Of course, the assembly and the annotation must be released prior to publication.
Response: Thanks for your instructive comments. Yes, the genome data of both female and male individuals were released from the NCBI. They are accessible at https://www.ncbi.nlm.nih.gov/bioproject/?term=PRJNA810746 and https://www.ncbi.nlm.nih.gov/bioproject/?term=PRJNA810753.
Related annotation files were uploaded to the CNSA: https://ftp.cngb.org/pub/CNSA/data2/CNP0002889/CNS0538329/CNA0047397/. Sequenced reads were also deposited at the CNSA for public availability: https://db.cngb.org/search/organism/113540/.

MINOR MODIFICATIONS
The experiment was well-designed and the presentation is complete. However, some aspects of the presentation should be clarified.
1. The paper must make clear that the sex-specific region was not found. The current title is ambiguous about this, and it led me to believe the region had been found. Line 296 says the discovered differences may cause sex divergence, but the paper should note that the true sex-determining region may not be among the candidates identified here.
Response: You are right. We changed the title to "Identification of candidate sex-specific genomic regions in male and female Asian arowana genomes" (lines 1-2).

2. The male assembly is a short-read assembly that was made chromosome-length using female Hi-C. Thus, long-range connectivity in the male assembly is partially derived from female fish. This is indicated in the Methods, but the analysis should note this limitation. For example, line 239 should specify, "female Hi-C". The analysis of chromosome rearrangements in Figures 2 and S6 could note that some rearrangements may be missing in the "femalized" male assembly.
Response: Thanks for your nice comments. We additionally sequenced the male individual by using PacBio HiFi and Hi-C platforms. Therefore, we obtained a new high-quality male genome assembly. We performed a solid synteny analysis to compare the new chromosome-level male genome assembly to the original female assembly again; interestingly, we identified three same rearrangements as in previous results between male and female individuals. We therefore think that these rearrangements could be truthfully existed with more confidence. More detailed software parameters were added in the revised manuscript, and related read data were uploaded and released for public availability to make our results maximumly reproductive by other researchers.

3. The assembly pipeline needs clarification. The existing description in Methods is technically correct but potentially confusing for some readers. Add sentences or a figure describing the male and female

pipelines from sequencing to assembly. Make clear the hybrid assembly process is not experimental i.e. it carefully follows processes that were previously published. For female, make clear it was polished with PacBio and polished again with Illumina. Describe the DBG2OLC hybrid step in its own sentence. State the value of K (K=17) near the formula that uses it. State how the Illumina data was "filtered."
Response: Thanks for your instructive advice and comments. Yes, we added a figure (as Supplementary Figure 3) to summarize the detailed assembling pipelines for female and male assemblies, making the assembly steps for both female and male genomes more clearly.

We added a sentence to introduce some previous studies that employed the similar hybrid assembly approach as follows in lines 147-149:
This hybrid assembly pipeline was well performed in many previous studies [20, 26, 27].

We revised the formula as follows (lines 133-135): G = N*(L–k-mer + 1)/K_depth, where k-mer length is defined as 17 bp, N is the total number of reads, and K_depth represents the frequency of occurrence more frequently than others.

For the Illumina data filtering, we revised this sentence as follows (line 129):
After filtering adapter sequences and low-quality reads by SOAPnuke v.1.5.6 with detailed parameters: filter -l 10 -q 0.1 -n 0.01 (RRID:SCR_015025) [14].

4. The Abstract says "an improved [female] reference" is presented, but the new assembly was not explicitly compared to the old ones. The text only implies (lines 80-84) that the old ones had shorter scaffolds and more gaps. It would improve the paper to include the assembly comparison. Does the new assembly contain more genes, more repeats, etc.? If such additional analysis is impossible, the abstract needs modification. The Introduction's off-hand remark that this species has been omitted from key studies due to poor quality of the previous assemblies (lines 85-86) must be justified or removed.
Response: Thanks for your nice advice. In fact, we compared the contig N50 values, gene numbers and repeat contents between this new female genome assembly and our previous one; the contig N50 of the new one was about 90 times more contiguous than the previous one. We also predicted about 3,000 more genes in this new female genome, although the repeats are similar in both assemblies. Related sentences were added in lines 250-255, 275-277 and 268-269.

5. Some unscientific language should be changed. The fish should not be subjectively called "famous" and "beautiful" in a science journal. (But perhaps a poem about the fish could be quoted?) Lines 59-60 claim that the fish is a "symbol of lucky … particularly in Asian countries"; the reference to a geography study leaves the claim unsupported so I think the whole sentence should be cut. The word "remarkable" is inappropriate to describe structural variation: lines 32 and 304. The abstract should say these assemblies "may" aid research, not "will" aid research (line 39). The statement that the assembly covers 98% of the genome (line 236) should clarify the comparison is to the genome size estimated from read coverage. The word "significant" must be changed or backed by statistics: line 290.
Response: Thanks for your instructive advice. Yes, we removed these words like “beautiful”, “symbol of lucky”, and ‘famous’ (in the abstract).

We changed ‘remarkable’ and ‘remarkably’ to ‘potential’ and ‘potentially’, and revised ‘will’ to ‘may’ in lines 34, 323, and 40 respectively.

For “98% of the genome”, we revised this sentence in lines 250-252 as follows:
The final assembly of the female individual consisted of 25 chromosomes and covered 765.8 Mb, which accounts for 98.0% of the assembled scaffolds.

We also changed the ‘significant’ to ‘potential’ in line 309.

6. Enhance the figure captions. Why does female chr 3 aligns to male chr 4? Why are alignments grouped by color in Figure S6? What is shown by the middle vertical color bar in Figure S5? What are the areas under the curves in key regions of the coverage plots in Figures S1 and S2? Perhaps insert a zoom-in histogram of the critical region so readers see each integer coverage level.
Response: Thanks for your good advice. Yes, we performed new genome-wide alignment and revised Figure 2 and Supplementary Figure 7, after we sequenced new HiFi and Hi-C data of the male and constructed a new male chromosome-level assembly.

The colors in Figure S6 were randomly selected by the RectChr software. For the Figure S5, color key

indicates log2-transformed transcription values; we added this description in the Supplementary Figure 6 (original Figure S5).

We supplemented the peak coverage of the kmer curves in captions of the Figures S1 and S2. For the integer coverage level of each inversion boundary (also known as breakpoint), we first applied the minimap2 with default parameters to align PacBio reads to the male genome assembly, and then used the Integrative Genomics Viewer software to show the detailed read coverage of the critical regions in Supplementary Figure 8.

RECOMMENDATIONS
This manuscript aligns well with the journal's instruction for authors that says, "One of the aims of a Data Note is to incentivize and more rapidly release data before subsequent detailed analysis has been carried out." However, the authors may want to improve this paper by analyzing some additional aspects of their data.
1. Address the male/female assembly quality difference. The paper says "both genome assemblies are indeed of high quality and completeness" based on BUSCO: line 246. However, the paper has indications of a difference in quality: the male had lower resequencing map rate (line 264) and lower repeat content (line 250). Provide the male contig N50, not just scaffold N50 (line 239). How would results have changed if you assembled the female from Illumina-only, as was done for male? What was gained from the additional resources invested in the female assembly?
Response: Thanks for your good questions and comments. In fact, we additionally sequenced PacBio HiFi and Hi-C data of the male individual, and obtained a new chromosome-level genome assembly for the male arowana. In this way, both male and female genomes are of high quality. We also compared the new male genome assembly with the female assembly, which confirms three same rearrangements as in previous results.

2. When female Hi-C was applied to male contigs, were any contig breaks suggested?
Response: We additionally sequenced Hi-C data of the male individual, and assembled a new chromosome-level genome assembly for the male arowana.

3. Of the intersex chromosome rearrangements found, what genes are nearby?
Response: We revised the Figure 2 and added detailed gene ids and functional descriptions near the inversion boundaries.

Reviewer #2:
This paper describes two new genome assemblies, as well as two experimental datasets, all aimed at understanding sex determination in the Asian arowana. In general, the data and methods used are well suited for addressing this issue.
There are a few issues I would like to ask the authors to comment on:
1. The methods for both genome assemblies (female and male) differ considerably, which might affect their respective qualities. The final results and comparative genomics, however, show that both assemblies are very similar, indicating that both approaches are appropriate. However, there are some peculiarities in the comparison that might reflect misassemblies. Specifically, the genome alignments suggest large rearrangements within several male and female chromosomes (figure 2). Should this be interpreted as the males and females being homozygotic for these rearrangements? How would this work, biologically? Are these chromosomes still compatible? Does for example a female receive both female copies of such chromosomes from the mother fish? As an alternative explanation, are female and male fish not from the same interbreeding population?
Response: Thanks for your instructive questions and comments. We additionally sequenced the male individual by using PacBio Hifi and Hi-C platforms. Therefore, we obtained a new high-quality male genome assembly.

After we performed a new synteny analysis to compare the new chromosome-level male genome assembly to the original female one, we confirmed three same rearrangements as in previous results between male and female individuals. We thus believe that these rearrangements could be truthfully existed.

We also added more detailed software parameters in the revised manuscript, and all read data were uploaded and released for public availability. By the way, both male and female fishes were collected from the same interbreeding population.

For biological interpretations, we consider that this sex-differential variance could be similar to the XY or

ZW sex-chromosomes in higher animals. Because arowana is an ancient teleost, the divergence between male and female specific chromosomes were not thoroughly evolutionary and taken shape. Only several preliminary inversions were identified in some chromosomes.

Ideally, I would like to see (by e.g. alignment) how these rearrangement points are supported by the different sequencing data sets.
Response: Thanks for your advice. Yes, we added detailed alignments (by the IGV software) in Supplementary Figure 8 after construction of the new male assembly with additional sequencing reads.

2. Related: It is not entirely clear if/how the HiC data processing affects the large-scale scaffolding. The HiC data are from females only, but also used for the males. As the large-scale structure is different (figure 2, supplementary figure 6), apparently this does not mean the female structure is imposed on the male data. But I am not convinced that comparing large-scale structure based on the same data is a good idea, in general.
Response: You are right. We additionally sequenced PacBio HiFi and Hi-C data of the male individual, and assembled a new chromosome-level genome assembly for the male arowana. We also compared the new male genome assembly with the female one, and identified three same rearrangements as in previous results. These new data and sex-differences are more convinced.

3. A previous publication on the Arowana genome by these authors (Bian 2016) suggests a ZW/ZZ chromosomal sex determination systems, in which females are the heterogametic sex containing a ZW pair. I see no indication of this in the new assemblies, is this correct?
Response: You are right. In fact, after obtaining high-quality chromosome-level genome assemblies of both male and female arowana, we did not confirm the heterogametic sex in the female genome. Therefore, we did not state this in our revised manuscript.

4. There are some ambiguities in the descriptions of the sequencing data. Illumina read lengths are not always mentioned, and sequencing statistics do not always agree between the text and tables:
GWAS data: supplementary table 2 has 709.2 Gbp raw, 605.7 Gbp mapped; l 263 has approximately 709.1 Gbp raw; but l 123 has 722.0 Gbp raw, 638.5 Gbp clean.
Assembly: methods do not list the 170 bp library (l 103); l 105 has 226.2 Gbp raw female, but supplementary table 1 only 73.4, and l 228 lists 98.1 Gbp clean data; l 108 (male) has 571.2 Gbp, but the table 294.4; l 113 27.4 Gbp clean PacBio are raw in supplementary table 1.
Response: Sorry for these mistakes. We carefully checked the details of sequenced reads and corrected these data throughout the revised manuscript.

5. The transcriptomics data and methods are not described in sufficient detail. For example, which normalization and statistical tests were used (using edgeR)? How many genes were tested, and how many are differentially expressed (supplementary figure 5)? Does the p-value threshold (line 195) take into account some form of multiple testing correction? What are the gene expression values in supplementary table 6 (e.g. per million reads, transcripts…)?
Response: Thanks for your advice and questions. Yes, the edgeR software was applied for normalization and statistical tests. A total of 25,328 genes were used as the input gene number, and 1,693 DEGs were identified for drawing the heatmap (Supplementary Figure 6). The p-value and fold changes were considered to identify the DEGs, although multiple testing correction has not been performed for the DEG identification. If we used the q-value, we would obtain too few DEG number. The gene expression values were calculated as FPKM (fragments per kilobase per million mapped reads). See related description in lines 197-207.

6. Since ovaries and testes are very different tissues, their expression profiles will be very different. This, in turn, invalidates scaling normalization procedures such as the edgeR default, which implicitly assume both conditions to be nearly identical apart from a few differentially expressed genes. In other words, it is not clear how to meaningfully compare apples with oranges using standard bulk RNA-seq bioinformatics.
Response: Thanks for your good questions. In fact, it is widely used for sex transcriptome comparison to map onto a male or female reference genome, because the male and female genomes of a same species are usually very similar in a large scale. Therefore, the mapping ratios of transcriptome reads and the calculation of expression values are not impacted by the different sex genomes.

However, it would be more valuable to compare these expression values of male and female individuals for identification of sex-related genes. For the comparison of apples and oranges, it couldn't be much meaningful, because the apple genome has largely different from the orange's, and these calculated

expression values will be incorrect.

7. Is there any reason to expect gene expression in mature gonads to be informative of sex determination earlier in development?
Response: No. In fact, it is more serious and meaningful to detect sex-determing genes at embryo stages or sex-differentiation stages by sequencing single cell transcriptome or spatial transcriptome. We are going to fulfil this task once a local government grant is available. On the other hand, for the expectation of gene expression in mature gonads, we can discover some sex-related genes for involvement in secretion of sex hormones.

8. The GWAS involves 15+15 samples, which I think is in general low for such a study? Perhaps this is different for a qualitative trait such as sex? Is the statistical power sufficient?
Response: It is a good question. For our GWAS, because the sex is a more confirmed and significant qualitative trait comparing to body size or weight, 15+15 samples are indeed facilitated to perform the GWAS analysis and discover the significant regions. Similarly, in our previous study, we resequenced only 10 casper zebrafish, 10 roy zebrafish and 10 wild-type zebrafish to correctly identify the divergence regions for skin transparent phenotype (Bian et al., 2020).
Bian, C., Chen, W., Ruan, Z., Hu, Z., Huang, Y., Lv, Y., Xu, T., Li, J., Shi, Q., & Ge, W. (2020). Genome and Transcriptome Sequencing of casper and roy Zebrafish Mutants Provides Novel Genetic Clues for Iridophore Loss. Int J Mol Sci, 21(7):2385.

On the other hand, the Asian arowana is one of the most expensive aquarium fishes, with a market value as much as $300,000 per individual (https://biologydictionary.net/arowana/). Since its sex cannot be distinguished by morphological appearance, we killed and dissected all sampled arowana individuals to check their testis or ovary for confirmation of sexual status. By the way, we tried our best to obtain a maximum number of arowana individuals for the resequencing analysis, although we wish to obtain more samples from other countries in our next project once a local government grant is available.

9. The BUSCO methods are not described. Which version, which reference?
Response: Thanks for your advice. We added more details about the BUSCO version 5.22 and Actinopterygii odb10 dataset with related references in line 261.

10. How were the RNA-seq data filtered (l 188)?
Response: We added detailed software and parameters for the RNA-seq data filtering as follows in lines 197-199:

Raw data were cleaned by discarding reads with adaptor sequences, >10% of non-sequenced bases, or >50% of low-quality bases by using SOAPnuke v.1.5.6 (parameters: filter -n 0.01 -l 15 -q 0.4 -G -Q 2, RRID: SCR_015025).

11. l 230: SSPACE-longer -> SSPACE-LongRead
Response: We revised this software name in lines 145 and 246.

12. l 128 genomes were predicted -> genome lengths were predicted
Response: Yes, it is corrected in line 133.

13. l 236/241: What does it mean that the final assembly covers 98.0/96.7% of the genome? Are the rest gaps from unmapped contigs?
Response: Yes, the rest gaps are the unmapped scaffolds (female) and contigs (male). We revised related sentences in lines 250-252 and 259 as follows:
The final assembly of the female individual consisted of 25 chromosomes and covered 765.8 Mb, which accounts for 98.0% of the assembled scaffolds.

Hi-C data were anchored to the draft assembly to form 25 chromosomes (ranging from 18.5 Mb to 53.0 Mb in length) and cover 747.2 Mb, which accounts for approximately 98.7% of the assembled contigs of the male individual.

Close
