# Supplementary material for: Identification of candidate sex‐specific genomic regions in male and female Asian arowana genomes
Source: Gigascience. 2022 Sep 15;11:giac085. doi: 10.1093/gigascience/giac085 (PMC9475665; doi:10.1093/gigascience/giac085)
Supplement: giac085_Response_to_Reviewer_Comments_Revision_1 [file giac085_response_to_reviewer_comments_revision_1.pdf]

Author's Response To Reviewer Comments

Close

I thank the authors for answering my questions and improving the manuscript. Including the new male assembly in the manuscript removes a major issue with the previous version, i.e. that large-scale comparisons between female and male genomes were based on large-scale information for the female genome only.
Response: Many thanks for your positive comments.

I am satisfied with most changes and responses, but would like to highlight two remaining issues:
1) The RNA-seq analysis remains relatively weak. I would suggest leaving it out alltogether. I do not agree with skipping the multiple testing correction for the reason indicated: 'If we used the q-value, we would obtain too few DEG number'. In addition, my comment about 'apples' and 'oranges' does not refer to the male and female genomes - here, I agree with the authors that using one genome for analyzing all transcriptome samples is practical and reasonable. Instead, I referred to comparing two very different tissues using methodology (differential expression analysis) that assumes very similar samples.
Response: Thanks for your suggestion. We agree your comment and removed the DEG related sentences and heatmap in our manuscript.

2) The (now more robustly demonstrated) structural chromosomal differences between the sexes remain surprising, and this needs to be discussed. New Suppl. Fig 8 shows that the structural rearrangement is homozygous in the male sample at least for Chr6 and Chr21 - Chr10 is possibly heterozygous. If the female genome is also homozygous for its structural variation (not shown - this could help!), the sex differences suggest an 'AA/BB' type of determination, instead of ZW/ZZ or XX/XY (assuming these structural differences even play a role in sex determination - which they may or may not). The problem is therefore that the male and female genomes suggest reproductive isolation from each other! How would a male get its two 'A' type chromosomes, if females can only contribute 'B' (and vice versa)? A more plausible explanation would be reproductive isolation because both sexes were sampled from different subspecies or populations, is this the case?
Response: Thanks for your comments. We added the alignments of female structure rearrangement in supplementary figure 8, and found Chr6 and Chr21 may be the heterozygous and Chr10 is possibly homozygous. For AA/BB, ZW/ZZ or XX/XY, we tend to consider that the arowana has not evolved a confirmed sex chromosome, similar to the zebrafish and grouper. There are only some genomic markers that could distinguish the female and male individuals.

For reproductive isolation, we could not consider the structural rearrangement could lead to the reproductive isolation, because genes have not been broken and disappeared. A possible example for no reproductive isolation is reported by ruff genome study [1], Satellite male individual and Faeder male individual have a 4.5 Mb inversion divergence in their genomes. But both of them have no reproductive isolation with female individual. On the other hand, for samples, both female and male are collected from one population.

1. Lamichhaney S, Fan G, Widemo F, Gunnarsson U, Thalmann DS, Hoeppner MP, et al. Structural genomic changes underlie alternative reproductive strategies in the ruff (Philomachus pugnax). Nat Genet. 2016;48 1:84-8. doi:10.1038/ng.3430.

Close
