# Supplementary material for: Identification of candidate sex‐specific genomic regions in male and female Asian arowana genomes
Source: Gigascience. 2022 Sep 15;11:giac085. doi: 10.1093/gigascience/giac085 (PMC9475665; doi:10.1093/gigascience/giac085)
Supplement: giac085_Reviewer_1_Report_Original_Submission — Jason R. Miller, MS -- 3/27/2022 Reviewed [file giac085_reviewer_1_report_original_submission.pdf]

**Reviewer Report**

**Title: Identification of candidate sexâ€�specific genomic regions in male and female Asian arowana genomes**

**Version: Original Submission** **Date:** 3/27/2022

**Reviewer name: Jason R. Miller, MS**

**Reviewer Comments to Author:**

REVIEW
Title: Sexâ€�specific genomic region identification in male and female Asian arowana genomes
SUMMARY
This manuscript presents a genomics analysis of a fish species, Scleropages formosus, for which there was only a draft genome previously. The authors present a high-quality female genome assembly and lower quality male genome, plus gene annotation, repeats analysis, BUSCO, transcriptomics, GWAS, and comparative genomics. These data helped identify some candidates for the sex-specific regions which remain elusive.
MAJOR ISSUE
The paper says the annotation files are available at NCBI. However, the NCBI pages say, "No public data is linked to this project." Of course, the assembly and the annotation must be released prior to publication.
MINOR MODIFICATIONS
The experiment was well-designed and the presentation is complete. However, some aspects of the presentation should be clarified.
1. The paper must make clear that the sex-specific region was not found. The current title is ambiguous about this, and it led me to believe the region had been found. Line 296 says the discovered differences may cause sex divergence, but the paper should note that the true sex-determining region may not be among the candidates identified here.
2. The male assembly is a short-read assembly that was made chromosome-length using female Hi-C. Thus, long-range connectivity in the male assembly is partially derived from female fish. This is indicated in the Methods, but the analysis should note this limitation. For example, line 239 should specify, "female Hi-C". The analysis of chromosome rearrangements in Figures 2 and S6 could note that some rearrangements may be missing in the "femalized" male assembly.
3. The assembly pipeline needs clarification. The existing description in Methods is technically correct but potentially confusing for some readers. Add sentences or a figure describing the male and female pipelines from sequencing to assembly. Make clear the hybrid assembly process is not experimental i.e. it carefully follows processes that were previously published. For female, make clear it was polished with PacBio and polished again with Illumina. Describe the DBG2OLC hybrid step in its own sentence. State the value of K (K=17) near the formula that uses it. State how the Illumina data was "filtered."
4. The Abstract says "an improved [female] reference" is presented, but the new assembly was not explicitly compared to the old ones. The text only implies (lines 80-84) that the old ones had shorter

scaffolds and more gaps. It would improve the paper to include the assembly comparison. Does the new assembly contain more genes, more repeats, etc.? If such additional analysis is impossible, the abstract needs modification. The Introduction's off-hand remark that this species has been omitted from key studies due to poor quality of the previous assemblies (lines 85-86) must be justified or removed.

5. Some unscientific language should be changed. The fish should not be subjectively called "famous" and "beautiful" in a science journal. (But perhaps a poem about the fish could be quoted?) Lines 59-60 claim that the fish is a "symbol of lucky … particularly in Asian countries"; the reference to a geography study leaves the claim unsupported so I think the whole sentence should be cut. The word "remarkable" is inappropriate to describe structural variation: lines 32 and 304. The abstract should say these assemblies "may" aid research, not "will" aid research (line 39). The statement that the assembly covers 98% of the genome (line 236) should clarify the comparison is to the genome size estimated from read coverage. The word "significant" must be changed or backed by statistics: line 290.

6. Enhance the figure captions. Why does female chr 3 aligns to male chr 4? Why are alignments grouped by color in Figure S6? What is shown by the middle vertical color bar in Figure S5? What are the areas under the curves in key regions of the coverage plots in Figures S1 and S2? Perhaps insert a zoom-in histogram of the critical region so readers see each integer coverage level.

RECOMMENDATIONS

This manuscript aligns well with the journal's instruction for authors that says, "One of the aims of a Data Note is to incentivize and more rapidly release data before subsequent detailed analysis has been carried out." However, the authors may want to improve this paper by analyzing some additional aspects of their data.

1. Address the male/female assembly quality difference. The paper says "both genome assemblies are indeed of high quality and completeness" based on BUSCO: line 246. However, the paper has indications of a difference in quality: the male had lower resequencing map rate (line 264) and lower repeat content (line 250). Provide the male contig N50, not just scaffold N50 (line 239). How would results have changed if you assembled the female from Illumina-only, as was done for male? What was gained from the additional resources invested in the female assembly?

2. When female Hi-C was applied to male contigs, were any contig breaks suggested?

3. Of the intersex chromosome rearrangements found, what genes are nearby?

**Level of Interest**

Please indicate how interesting you found the manuscript: Choose an item.

**Quality of Written English**

Please indicate the quality of language in the manuscript: Choose an item.

**Declaration of Competing Interests**

Please complete a declaration of competing interests, considering the following questions:

- Have you in the past five years received reimbursements, fees, funding, or salary from an organisation that may in any way gain or lose financially from the publication of this manuscript, either now or in the future?
- Do you hold any stocks or shares in an organisation that may in any way gain or lose financially from the publication of this manuscript, either now or in the future?
- Do you hold or are you currently applying for any patents relating to the content of the manuscript?
- Have you received reimbursements, fees, funding, or salary from an organization that holds or has applied for patents relating to the content of the manuscript?
- Do you have any other financial competing interests?
- Do you have any non-financial competing interests in relation to this paper?

If you can answer no to all of the above, write 'I declare that I have no competing interests' below. If your reply is yes to any, please give details below.

I declare that I have no competing interests

I agree to the open peer review policy of the journal. I understand that my name will be included on my report to the authors and, if the manuscript is accepted for publication, my named report including any attachments I upload will be posted on the website along with the authors' responses. I agree for my report to be made available under an Open Access Creative Commons CC-BY license (http://creativecommons.org/licenses/by/4.0/). I understand that any comments which I do not wish to be included in my named report can be included as confidential comments to the editors, which will not be published.

Choose an item.

To further support our reviewers, we have joined with Publons, where you can gain additional credit to further highlight your hard work (see: https://publons.com/journal/530/gigascience). On publication of this paper, your review will be automatically added to Publons, you can then choose whether or not to claim your Publons credit. I understand this statement.

Yes Choose an item.
