# Supplementary material for: Identification of candidate sex‐specific genomic regions in male and female Asian arowana genomes
Source: Gigascience. 2022 Sep 15;11:giac085. doi: 10.1093/gigascience/giac085 (PMC9475665; doi:10.1093/gigascience/giac085)
Supplement: giac085_Reviewer_2_Report_Original_Submission — Christiaan Henkel -- 4/4/2022 Reviewed [file giac085_reviewer_2_report_original_submission.pdf]

Reviewer Report

Title: Identification of candidate sexâ€specific genomic regions in male and female Asian arowana genomes

Version: Original Submission **Date:** 4/4/2022

Reviewer name: Christiaan Henkel

Reviewer Comments to Author:

This paper describes two new genome assemblies, as well as two experimental datasets, all aimed at understanding sex determination in the Asian arowana. In general, the data and methods used are well suited for addressing this issue.

There are a few issues I would like to ask the authors to comment on:

1. The methods for both genome assemblies (female and male) differ considerably, which might affect their respective qualities. The final results and comparative genomics, however, show that both assemblies are very similar, indicating that both approaches are appropriate. However, there are some peculiarities in the comparison that might reflect misassemblies. Specifically, the genome alignments suggest large rearrangements within several male and female chromosomes (figure 2). Should this be interpreted as the males and females being homozygotic for these rearrangements? How would this work, biologically? Are these chromosomes still compatible? Does for example a female receive both female copies of such chromosomes from the mother fish? As an alternative explanation, are female and male fish not from the same interbreeding population?

Ideally, I would like to see (by e.g. alignment) how these rearrangement points are supported by the different sequencing data sets.

2. Related: It is not entirely clear if/how the HiC data processing affects the large-scale scaffolding. The HiC data are from females only, but also used for the males. As the large-scale structure is different (figure 2, supplementary figure 6), apparently this does not mean the female structure is imposed on the male data. But I am not convinced that comparing large-scale structure based on the same data is a good idea, in general.

3. A previous publication on the Arowana genome by these authors (Bian 2016) suggests a ZW/ZZ chromosomal sex determination systems, in which females are the heterogametic sex containing a ZW pair. I see no indication of this in the new assemblies, is this correct?

4. There are some ambiguities in the descriptions of the sequencing data. Illumina read lengths are not always mentioned, and sequencing statistics do not always agree between the text and tables:

GWAS data: supplementary table 2 has 709.2 Gbp raw, 605.7 Gbp mapped; l 263 has approximately 709.1 Gbp raw; but l 123 has 722.0 Gbp raw, 638.5 Gbp clean.

Assembly: methods do not list the 170 bp library (l 103); l 105 has 226.2 Gbp raw female, but supplementary table 1 only 73.4, and l 228 lists 98.1 Gbp clean data; l 108 (male) has 571.2 Gbp, but the table 294.4; l 113 27.4 Gbp clean PacBio are raw in supplementary table 1.

5. The transcriptomics data and methods are not described in sufficient detail. For example, which normalization and statistical tests were used (using edgeR)? How many genes were tested, and how

many are differentially expressed (supplementary figure 5)? Does the p-value threshold (line 195) take into account some form of multiple testing correction? What are the gene expression values in supplementary table 6 (e.g. per million reads, transcripts…)?
6. Since ovaries and testes are very different tissues, their expression profiles will be very different. This, in turn, invalidates scaling normalization procedures such as the edgeR default, which implicitly assume both conditions to be nearly identical apart from a few differentially expressed genes. In other words, it is not clear how to meaningfully compare apples with oranges using standard bulk RNA-seq bioinformatics.
7. Is there any reason to expect gene expression in mature gonads to be informative of sex determination earlier in development?
8. The GWAS involves 15+15 samples, which I think is in general low for such a study? Perhaps this is different for a qualitative trait such as sex? Is the statistical power sufficient?
9. The BUSCO methods are not described. Which version, which reference?
10. How were the RNA-seq data filtered (l 188)?
11. l 230: SSPACE-longer -> SSPACE-LongRead
12. l 128 genomes were predicted -> genome lengths were predicted
13. l 236/241: What does it mean that the final assembly covers 98.0/96.7% of the genome? Are the rest gaps from unmapped contigs?

Level of Interest

Please indicate how interesting you found the manuscript: Choose an item.

Quality of Written English

Please indicate the quality of language in the manuscript: Choose an item.

Declaration of Competing Interests

Please complete a declaration of competing interests, considering the following questions:

- Have you in the past five years received reimbursements, fees, funding, or salary from an organisation that may in any way gain or lose financially from the publication of this manuscript, either now or in the future?
- Do you hold any stocks or shares in an organisation that may in any way gain or lose financially from the publication of this manuscript, either now or in the future?
- Do you hold or are you currently applying for any patents relating to the content of the manuscript?
- Have you received reimbursements, fees, funding, or salary from an organization that holds or has applied for patents relating to the content of the manuscript?
- Do you have any other financial competing interests?

- Do you have any non-financial competing interests in relation to this paper?

If you can answer no to all of the above, write 'I declare that I have no competing interests' below. If your reply is yes to any, please give details below.

I declare that I have no competing interests

I agree to the open peer review policy of the journal. I understand that my name will be included on my report to the authors and, if the manuscript is accepted for publication, my named report including any attachments I upload will be posted on the website along with the authors' responses. I agree for my report to be made available under an Open Access Creative Commons CC-BY license (http://creativecommons.org/licenses/by/4.0/). I understand that any comments which I do not wish to be included in my named report can be included as confidential comments to the editors, which will not be published.

Choose an item.

To further support our reviewers, we have joined with Publons, where you can gain additional credit to further highlight your hard work (see: https://publons.com/journal/530/gigascience). On publication of this paper, your review will be automatically added to Publons, you can then choose whether or not to claim your Publons credit. I understand this statement.

Yes Choose an item.
