# Supplementary material for: Identification of candidate sex‐specific genomic regions in male and female Asian arowana genomes
Source: Gigascience. 2022 Sep 15;11:giac085. doi: 10.1093/gigascience/giac085 (PMC9475665; doi:10.1093/gigascience/giac085)
Supplement: giac085_Reviewer_2_Report_Revision_1 — Christiaan Henkel -- 7/7/2022 Reviewed [file giac085_reviewer_2_report_revision_1.pdf]

Reviewer Report

Title: Identification of candidate sexâ€specific genomic regions in male and female Asian arowana genomes

Version: Revision 1 **Date:** 7/7/2022

Reviewer name: Christiaan Henkel

Reviewer Comments to Author:

I thank the authors for answering my questions and improving the manuscript. Including the new male assembly in the manuscript removes a major issue with the previous version, i.e. that large-scale comparisons between female and male genomes were based on large-scale information for the female genome only. I am satisfied with most changes and responses, but would like to highlight two remaining issues:

1) The RNA-seq analysis remains relatively weak. I would suggest leaving it out alltogether. I do not agree with skipping the multiple testing correction for the reason indicated: 'If we used the q-value, we would obtain too few DEG number'. In addition, my comment about 'apples' and 'oranges' does not refer to the male and female genomes - here, I agree with the authors that using one genome for analyzing all transcriptome samples is practical and reasonable. Instead, I referred to comparing two very different tissues using methodology (differential expression analysis) that assumes very similar samples.

2) The (now more robustly demonstrated) structural chromosomal differences between the sexes remain surprising, and this needs to be discussed. New Suppl. Fig 8 shows that the structural rearrangement is homozygous in the male sample at least for Chr6 and Chr21 - Chr10 is possibly heterozygous. If the female genome is also homozygous for its structural variation (not shown - this could help!), the sex differences suggest an 'AA/BB' type of determination, instead of ZW/ZZ or XX/XY (assuming these structural differences even play a role in sex determination - which they may or may not). The problem is therefore that the male and female genomes suggest reproductive isolation from each other! How would a male get its two 'A' type chromosomes, if females can only contribute 'B' (and vice versa)? A more plausible explanation would be reproductive isolation because both sexes were sampled from different subspecies or populations, is this the case?

Level of Interest

Please indicate how interesting you found the manuscript: Choose an item.

Quality of Written English

Please indicate the quality of language in the manuscript: Choose an item.

Declaration of Competing Interests

Please complete a declaration of competing interests, considering the following questions:

- Have you in the past five years received reimbursements, fees, funding, or salary from an organisation that may in any way gain or lose financially from the publication of this manuscript, either now or in the future?
- Do you hold any stocks or shares in an organisation that may in any way gain or lose financially from the publication of this manuscript, either now or in the future?
- Do you hold or are you currently applying for any patents relating to the content of the manuscript?
- Have you received reimbursements, fees, funding, or salary from an organization that holds or has applied for patents relating to the content of the manuscript?
- Do you have any other financial competing interests?
- Do you have any non-financial competing interests in relation to this paper?

If you can answer no to all of the above, write 'I declare that I have no competing interests' below. If your reply is yes to any, please give details below.

I declare that I have no competing interests

I agree to the open peer review policy of the journal. I understand that my name will be included on my report to the authors and, if the manuscript is accepted for publication, my named report including any attachments I upload will be posted on the website along with the authors' responses. I agree for my report to be made available under an Open Access Creative Commons CC-BY license (http://creativecommons.org/licenses/by/4.0/). I understand that any comments which I do not wish to be included in my named report can be included as confidential comments to the editors, which will not be published.

Choose an item.

To further support our reviewers, we have joined with Publons, where you can gain additional credit to further highlight your hard work (see: https://publons.com/journal/530/gigascience). On publication of this paper, your review will be automatically added to Publons, you can then choose whether or not to claim your Publons credit. I understand this statement.

Yes Choose an item.
